# Genetic association with high‐resolution climate data reveals selection footprints in the genomes of barley landraces across the Iberian Peninsula

**DOI:** 10.1111/mec.15009

**Published:** 2019-04-02

**Authors:** Bruno Contreras‐Moreira, Roberto Serrano‐Notivoli, Naheif E. Mohammed, Carlos P. Cantalapiedra, Santiago Beguería, Ana M. Casas, Ernesto Igartua

**Affiliations:** ^1^ Estación Experimental de Aula Dei (EEAD‐CSIC) Zaragoza Spain; ^2^ Fundación ARAID Zaragoza Spain; ^3^ Faculty of Agriculture, Agronomy Department Sohag University Sohag Egypt

**Keywords:** agriculture, agroclimatic indices, barley, genome‐wide association analysis, selection footprint

## Abstract

Landraces are local populations of crop plants adapted to a particular environment. Extant landraces are surviving genetic archives, keeping signatures of the selection processes experienced by them until settling in their current niches. This study intends to establish relationships between genetic diversity of barley (*Hordeum vulgare* L.) landraces collected in Spain and the climate of their collection sites. A high‐resolution climatic data set (5 × 5 km spatial, 1‐day temporal grid) was computed from over 2,000 temperature and 7,000 precipitation stations across peninsular Spain. This data set, spanning the period 1981–2010, was used to derive agroclimatic variables meaningful for cereal production at the collection sites of 135 barley landraces. Variables summarize temperature, precipitation, evapotranspiration, potential vernalization and frost probability at different times of the year and time scales (season and month). SNP genotyping of the landraces was carried out combining Illumina Infinium assays and genotyping‐by‐sequencing, yielding 9,920 biallelic markers (7,479 with position on the barley reference genome). The association of these SNPs with agroclimatic variables was analysed at two levels of genetic diversity, with and without taking into account population structure. The whole data sets and analysis pipelines are documented and available at https://eead-csic-compbio.github.io/barley-agroclimatic-association. We found differential adaptation of the germplasm groups identified to be dominated by reactions to cold temperature and late‐season frost occurrence, as well as to water availability. Several significant associations pointing at specific adaptations to agroclimatic features related to temperature and water availability were observed, and candidate genes underlying some of the main regions are proposed.

## 1 INTRODUCTION

Landraces are populations of crop plants adapted to a particular environment, through a long history of cultivation by local farmers (Zeven, 1998). Landraces are valuable materials for phylogeographic studies (see review in Newton et al., 2010). They are supposed to bear the genetic signatures of adaptation to the environments in which they were developed, including human preferences. These selection footprints can be investigated with genomics tools. The study of patterns in the genomes of extant landraces in relation to the climate of their collection sites can indicate which adaptive processes were responsible for their distribution (Jones et al., 2008). However, extant landraces will provide a partial evolutionary history of crops (Fuller, Willcox, & Allaby, 2011), and the hypotheses emerging from their study should ultimately be put to test against archaeological data. Barley (*Hordeum vulgare* L.) is one of the main cereals, fourth in the World, after maize, rice and wheat. It is a diploid species (*n = *7) with a very large genome (about 5 Gb), and a recently published high quality genome sequence (Mascher et al., 2017). Its adaptive history can shed light on the context of the Neolithic expansion, because barley was part of the Neolithic package of crops that spread over Europe and the Mediterranean basin 10,000 to 7,000 YBP.

Spanish landraces are an appropriate subject to study barley adaptation. On the one hand, the Iberian Peninsula displays a rather wide variety of climates (De Castro, Martín‐Vide, & Brunet, 2005), presenting a diversity of ecological habitats (Manzano‐Piedras, Marcer, Alonso‐Blanco, & Picó, 2014; Myers, Mittermeier, Mittermeier, Fonseca, & Kent, 2000). On the other hand, it has received a wide diversity of barley germplasm since the Neolithic up until the middle Ages (Fischbeck, 2002; Komatsuda et al., 2007). Ancient and later arrivals of plant materials encountered this variety of climates and surely underwent a process of selection and adaptation. First, the germplasm groups arriving in the Peninsula prevailed in areas where they found appropriate niches. These groups then hybridized with each other to some extent in boundary regions, and evolved locally through mutations (Casas et al., 2018) and recombination, therefore producing new alleles and new allelic combinations that may have provided more ground for adaptation and selection. In fact, barley landraces from Spain are far from homogeneous. At least four different germplasm groups were identified (Yahiaoui et al., 2008), pointing at different routes of entry, in a parallel process to the one proposed for wheat (Moragues, García del Moral, Moralejo, & Royo, 2006a, 2006b). Our hypothesis is that the distribution of these groups in the Iberian Peninsula followed routes characterized by yet unknown environmental features, and settled according to suitability to environmental niches.

The Spanish Barley Core Collection (SBCC) was compiled as a balanced representation of the crop cultivated in the country until the second half of the 20th century (Igartua et al., 1998). It has been studied extensively, showing distinct agronomic and genetic features that highlight its interest as research tool for prebreeding and gene mining (Igartua et al., 1998). Some genotypes of this collection demonstrated good agronomic performance in stressed environments (Yahiaoui et al., 2014), even out‐yielding modern cultivars, probably due to their prolonged adaptation to Mediterranean climates.

This work investigates the association of genetic markers with a set of environmental features, including agroclimatic indices (measures or indicators of an aspect of the climate that has specific agricultural significance), chosen based on their relevance for agriculture of winter cereals. With this aim, we used the SBCC and a high‐resolution climatic data set specifically assembled for this purpose. We assessed this relationship at two levels, with and without considering population structure, with two different and complementary objectives. Life history traits like growth cycle duration and morphology of reproductive organs are strongly affected by natural and artificial selection, and can be easily confounded with population structure (Fournier‐Level et al., 2013; Leinonen, Remington, Leppälä, & Savolainen, 2013). Therefore, analyses that remove population structure could also remove genetic variation related to life history traits (Russell et al., 2016), making it difficult to detect some of the genetic factors responsible for adaptation. To circumvent this, we first tested the relationship of the distribution of germplasm groups detected through population structure analysis and their genetic polymorphisms to agroclimatic variables. We expected that these analyses would point at genomic regions that have driven the adaptation and differentiation of germplasm groups reflecting their history of evolution in distinct agroecological areas. Second, we searched for associations taking into account population structure, with the goal of unravelling polymorphisms that appear linked to regions active in adaptation at a finer scale, either within germplasm groups or after local group admixture.

We believe the processes that have shaped the diversity of Spanish barleys are typical of the expansion of crops outside their centres of origin, and that these analyses provide a case in point for the usefulness of environmental association analysis (EAA) to shed light on adaptation processes affecting crop landraces.

## 2 MATERIALS AND METHODS

### 2.1 Spanish barley core collection

This collection (SBCC, http://www.eead.csic.es/barley/index.php?Ing=1) was assembled as a representative set of the barleys cultivated in Spain prior to the introduction of modern cultivars. These landraces had passport data, stating geographic coordinates (longitude, latitude and altitude) of collection sites, and were systematically selected (Igartua et al., 1998) from a set of nearly 2,000 local accessions held at the Spanish National Bank of Phytogenetic Resources (CRF‐INIA). Initially, a set of 159 inbred lines was derived after three generations of selfing starting from single spikes collected from the same number of landraces. This collection was later reduced to 140 inbred lines, due to some seed losses and to the removal of 17 duplicates detected with molecular markers. Considering only mainland accessions (Balearic and Canary islands excluded), a set of 135 landraces remained, comprising 11 two‐rowed types, and 124 six‐rowed ones (Supporting Information Table S1).

### 2.2 Genotyping

Single nucleotide polymorphism (SNP) genotyping was carried out with the Illumina Infinium iSelect 9k SNP chip (Comadran et al., 2012), with 7,864 SNP assays, processed at TraitGenetics GmbH, (Gatersleben, Germany). Additionally, the collection was genotyped at Diversity Arrays Technology (Yarralumla, Australia), with the DArTseq technology (Kilian et al., 2012). This system combines complexity reduction methods with next‐generation sequencing platforms, targeting primarily genic regions. It produces two types of markers, classical SNP and presence/absence variation, also named SilicoDArTs. Only the SNPs were considered in this study. After merging Infinium DArTseq markers, the resulting number of SNPs was 9,920 (6,509 Infinium, 3,411 DArTseq). These were further reduced to 8,457 biallelic loci after removing those with more than 10% missing data (Supporting Information Table S2).

Physical positions of these markers were queried by aligning their sequences to the Barley refseq v1.0 genome assembly (Mascher et al., 2017) with default parameters in barleymap (Cantalapiedra, Boudiar, Casas, Igartua, & Contreras‐Moreira, 2015). The reported end coordinates for each marker were recorded. Markers matching unmapped contigs (chrUn) or with multiple mappings, spanning different chromosomes or more than 200 kb apart, were discarded. After quality checks, 7,479 markers (5,261 Infinium, 2,218 DArTseq) were assigned unique physical positions, and their respective genetic positions (in cM) taken from the closest marker in the POPSEQ2017 map (Beier et al., 2017). The genetic positions of markers SCRI_RS_224392, SCRI_RS_187102, SCRI_RS_167463, BOPA2_12_30895, and BOPA2_12_30894 were interpolated based on their physical distances. Map positions are available in Supporting Information Table S3.

### 2.3 Calculation of agroclimatic variables

The raw climatic data set was provided by the Spanish meteorological agency (AEMET). Daily data from 2,087 observatories of temperature and 6,952 of precipitation, evenly distributed over mainland Spain, were used (Supporting Information Figure S1). The data set spanned the most recent standard period of the World Meteorological Organization (WMO, 1981 to 2010), and all the observatories provided more than 10 years of data. A thorough reconstruction procedure was applied to the original precipitation and temperature data by using the reddprec r package (Serrano‐Notivoli, de Luis, & Beguería, 2017), and following the methodology described in Serrano‐Notivoli, de Luis, Saz, and Beguería (2017). This included: (a) an exhaustive quality control to remove anomalous or suspect data; and (b) the imputation of new values to all the missing data, to have serially‐complete data series covering the whole study period. This method, originally developed for daily precipitation, was adapted to daily temperature data by applying the quality control through the rclimdex v.1.1 software, developed by the WMO (Zhang & Yang, 2004). The reconstructed data series were used to create, using again the reddprec r package, three gridded data sets of daily maximum and minimum temperature and precipitation in the 1981–2010 period, covering the Iberian Peninsula with a spatial resolution of 5 × 5 km. An example of the daily grids is provided in Supporting Information Figure S2.

Daily gridded data were then used to compute a set of 147 *agroclimatic variables* (ACV) related to the development of winter cereals, defined in Table 1. Some of them (monthly and seasonal *pcp*, *tmed*, *tmax* and *tmin*) were derived by temporal aggregation of the daily grids of precipitation, mean, maximum and minimum temperature. Daily data were also used to compute other variables such as the thermal amplitude, *tamp*, which is the (monthly, seasonal) mean daily difference between *tmax* and *tmin*; the number of frost days, *frost*, or days where *tmin* *<*0°C; the late‐season frost probability, *pfrost*, which is the average first day in the year where the probability of *tmin* *<*0°C is lower or equal to 10%, that is, corresponding to a mean return period of one in 10 years. Daily data was also used for computing monthly potential vernalization, *verna*, which accounts for the required exposure to cold temperatures for winter cereal to start flowering. Vernalization was computed at the daily level based on the maximum and minimum temperatures, assuming that temperature describes a sine curve during the day, as done in the CERES‐Wheat model (Ritchie, 1991), with temperature thresholds modified for barley as in Ciudad (2002), following Dr Roger B. Austin (personal communication). The number of vernalization days was computed assuming that vernalization becomes effective at 0°C, then increases linearly until 4°C, and decreases linearly between 8 and 15°C. No interactions between daily temperature and cumulative degree days, or length of the photoperiod were considered as these need to be calibrated for each cultivar, so we called this variable “potential vernalization” as it depends only on climate. In addition, the mean number of days since an average sowing day, estimated as 15 November, required to accumulate 10, 20, 30 and 40 potential vernalization days, *verna_Nd*, were also computed: 15 November was considered as a typical sowing date for all Spain, according to the authors’ experience, and to the data reported by Supit and Wagner (1999), who found that 59% of barley sowings all over Spain had occurred by 10 November. We added five more days to account for seed imbibition, as vernalization acts on active tissues. Reference evapotranspiration data, according to the Penman‐Monteith equation as explained in the FAO56 manual (Allen, Pereira, Raes, & Smith, 1998), *ET_o_*, were obtained from a gridded data set for Spain (Tomas‐Burguera, Vicente‐Serrano, Grimalt, & Beguería, 2017; Vicente‐Serrano et al., 2017). The original data was down‐sampled from the initial resolution of 1.1 × 1.1 to 5 × 5 km by averaging the values, and the original weekly resolution was aggregated to monthly values. The time period 1981–2010 was selected in accordance to the rest of the climatic data. In addition to monthly, seasonal and annual mean values, *ET_o_* was further used to compute the climatic water balance, *bal*, as the difference between the cumulative precipitation and the cumulative *ET_o_*. The complete climatic data set is available in Supporting Information Table S4.

**Table 1:** List of agroclimatic and geographic variables used in this study. One hundred and forty‐seven variables were initially used, from which 20 were selected as most representative and least redundant via cluster analysis

Acronym	Variable description and unit	Scale of aggregation
pcp	Average cumulative precipitation (mm)	Season, month
tmed	Average daily mean temperature (°C)	Season, month
tmax	Average daily max temperature (°C)	Season, month
tmin	Average daily min temperature (°C)	Season, month
tamp	Average daily thermal amplitude (°C)	Season, month
frost	Average number of frost days (—)	Season, month
pfrost	Average first day in the year where P(tmin <0) ≤0.10	Annual
verna	Average potential vernalization (days) (—)	Month
verna_*N*d	Average number of days since 15th November to reach *n = *10, 20, 30 and 40 vernalization days (—)	Annual
ET_0_	Average cumulative reference evapotranspiration (mm)	Annual, season, month
bal	Climatic water balance (pcp—eto) (mm)	Annual, season, month
lon	Longitude, in UTM zone 30N projection (km)	—
lat	Latitude, in UTM zone 30N projection (km)	—
alt	Elevation (m above mean sea level)	—
dummy	Random data with spatial coherence (—)	—

For each climatic variable with the exception of *pfrost* and *verna_Nd*, monthly, seasonal and annual averages were calculated based on daily data. Monthly values are indicated after the variable acronym with suffixes *_jan* to *_dec*, while seasonal and annual values are denoted by *_spr* (spring, covering from March to April), *_aut* (September to November), *_win* (December to February) and *_annual*. Summer aggregates and the months between July and October are not expected to have influence on barley growth and, hence, were excluded from further analysis. Additionally, three geographical variables were included: *lon*, *lat* and *alt*, which stand for latitude, longitude and elevation. Latitude and longitude were extracted directly from the grid structure, and elevation data was obtained from the GTOPO30 digital elevation model developed by USGS (LP DAAC, 1996).

In addition to the environmental variables, 12 dummy variables were generated and included in the data set. Dummy variables are randomly generated synthetic variables that are incorporated into the analysis in order to test the robustness of the results with respect to type I errors (false positives), since it is known in advance that there is no true relationship between these variables and the dependent variable. Thus, an inflated (unexpectedly high) number of false positives with the dummy variables would raise a warning against the results obtained regarding the true independent variables. In our case the dummy variables were spatially correlated random fields (grids) generated by unconditional Gaussian simulation. The use of spatially correlated random fields instead of pure random (uncorrelated) variables corrects for the inflation of type I errors when this effect is not considered in the analysis of spatial environmental variables (Beguería & Pueyo, 2009). We computed 12 dummy variables by the unconditional Gaussian simulation algorithm using the gstat r package (Pebesma, 2004; and example code in Beguería, 2010), and extracted the values at the landrace collection sites. The degree of spatial correlation in the resulting grids is controlled by a semi‐variogram model, which is used for computing the interpolation weights and, depending on its parameterization, can vary from a totally random spatial field to a smoothly varying one (Cressie, 1993). In this case, the parameters of the semi‐variogram model used were chosen to ensure a degree of spatial smoothing similar to that of the climatic variables (Supporting Information Figure S3).

### 2.4 Selection of agroclimatic variables

**Figure 1:**
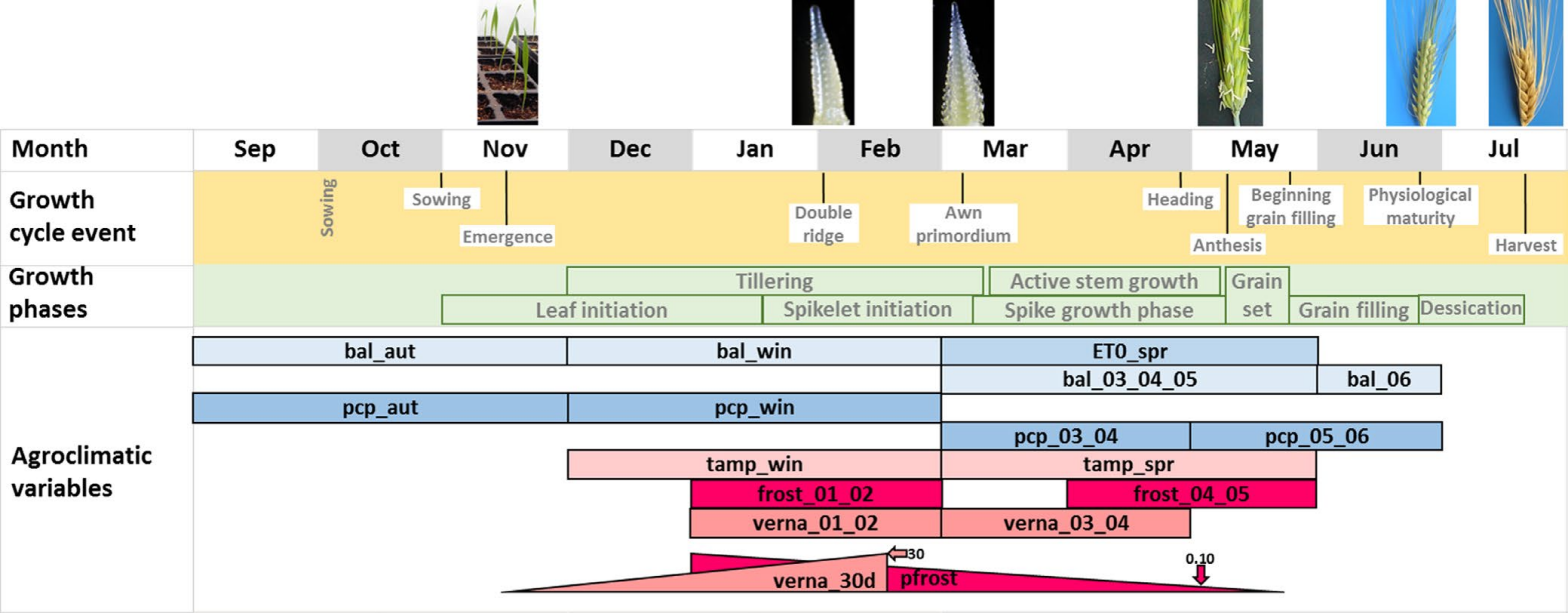
Diagram illustrating the agroclimatic variables selected for this study, matched to the milestones and phases of growth of the barley plant. Latitude, longitude and altitude are not represented. Dates correspond to an average autumn sowing in the Iberian Peninsula. Phases and milestones adapted from Slafer and Rawson (1994), and Sreenivasulu and Schnurbusch (2012)

Exploratory analysis of the agroclimatic variables revealed substantial covariance, as shown in Supporting Information Figure S4. Consequently, the variables were subjected to hierarchical cluster analysis in order to detect groups of similar variables. The Ward's D2 algorithm (Murtagh & Legendre, 2014) implemented in the r function hclust (R Core Team, 2017) was applied to the Euclidean distance matrix of the variables scaled and centred using the r function scale (R Core Team, 2017). The resulting dendrogram was then cut into 10 clusters (Supporting Information Figure S5) and one or, at most, two variables representative for each group were then selected, considering periods that matched the growth phases and the occurrence of the main growth milestones of barley, as described in Slafer and Rawson (1994) and Sreenivasulu and Schnurbusch (2012). The duration and dates for these phases and events were estimated by the authors, assuming an average autumn sowing for the Iberian Peninsula. The following 20 variables that were kept for further analyses: lon, lat, alt, pcp_aut, pcp_win, pcp_mar_apr, pcp_may_jun, ET_0__spr, bal_aut, bal_win, bal_mar_apr_may, bal_jun, tamp_win, tamp_spr, verna30d, verna_jan_feb, verna_mar_apr, frost_jan_feb, frost_apr_may, and pfrost (Figure 1). In some cases, multi‐month variables were computed by summing the values of the corresponding months, which belonged in the same cluster (for instance, pcp_mar_apr corresponds to the aggregated precipitation of March and April). Examples of these variables are shown in Supporting Information Figure S6.

Additionally, all the variables analyzed were subjected to a principal component analysis. The r function prcomp (R Core Team, 2017) was applied to the covariance matrix of the variables, after scaling and centering as explained above. The first three principal components (PC1‐3), which account for 55%, 17% and 10% of the variance, respectively, were extracted and treated as environmental variables for further analysis. The first principal component of the environmental variables (PC1) was positively correlated to vernalization, number of frost days, late frost probability and altitude, and negatively correlated to winter and spring temperature (Supporting Information Figure S7). The second component (PC2) was positively correlated to autumn, winter and spring precipitation and climatic water balance, and negatively correlated with the temperature amplitude in autumn, winter and spring (Supporting Information Figure S8). The third component was positively correlated with spring temperature amplitude and with the winter climatic water balance, and negatively correlated with the winter potential evapotranspiration and precipitation and water balance in June (Supporting Information Figure S9). Spatial distribution of PC1 presented high values on the mountain ranges and in the northern half of the Iberian Peninsula. PC2 had high values on the northern and north‐western rims of the Iberian Peninsula, as well as some other areas of Atlantic influence. PC3 distribution clearly identified the influence of the Mediterranean in the winter and spring climatology of the Iberian Peninsula (Supporting Information Figure S10).

### 2.5 Genome‐wide association between biallelic SNPs and agroclimatic variables

This step was performed with two different software packages that use different approaches, to minimize false positives. Seventeen selected agroclimatic variables, three geographic variables, plus 12 dummy variables, were standardized and formatted to be used as input environfile for software bayenv2 (https://gcbias.org/bayenv, version tguenther‐bayenv2_public‐8e4039f64d61, Günther & Coop, 2013), and software lfmm_v1.5 (Frichot, Schoville, Bouchard, & François, 2013). For both analyses, we treated each accession as being sampled from a different subpopulation, as in Russell et al. (2016).

In bayenv2, matrices of covariance between SNP genotypes were computed, to account for the background similarity among landraces. Instead of using all SNPs, a set of 711 nonredundant markers with linkage disequilibrium *r* ^2^ < 0.2 (computed on a window of five neighbors at each side) and unique genetic positions was shortlisted for this task (Supporting Information Table S5), as recommended by the software developer. Ten runs of bayenv2, with different random seeds and 100 K iterations each, were performed and the average final matrix computed, named *SBCCmatrix_nr_mean.txt*. Supporting Information Figure S11 shows that this matrix reproduces the known population structure of these barleys. This matrix was used for the conventional bayenv2 analysis (*covariance model*). An identity matrix was also formatted to model a null covariance matrix and named *SBCCmatrix_null.txt*, to be used for the analysis disregarding population structure (*null model*).

A snpsfile containing allele counts across 135 barley landraces was formatted, where each SNP is represented by two lines in the file, with the counts of allele 1 on the first line and the counts for allele 2 on the second. The resulting file was named *SBCC_9K_SNPs.tsv* and used for association mapping with a custom Perl script that parallelized bayenv2 jobs with the following parameters: *‐t ‐i SBCC_9K_SNPs.tsv ‐p 135 ‐e SBCC_environfile.tsv ‐n 21 ‐m SBCCmatrix_nr_mean.txt ‐k 100000 ‐c*. Five replicates per model (null and covariance), with different random seed each and 100 K iterations, were ran and the median Bayes factors (BF) and Spearman correlations (rho) were computed for each marker (Supporting Information Figure S12). For both models, we report the consensus set of SNPs within the top 1% of Bayes factors distribution that were also in the top 1% of absolute correlations in each of the five runs, for the combined distribution of results for the 20 agroclimatic variables. Additionally, we report thresholds based on the distribution of BF values calculated for the 12 dummy variables, i.e., a true null distribution. The threshold was set at percentile 99.99 of this distribution.

A previous version of lfmm was seen to be highly sensitive to the presence of missing data. Therefore, missing data were imputed using r package limkin, which provides an imputation routine particularly suited to homozygous individuals (Xu, Wu, Gonda, & Wu, 2015). After imputation and filtering for MAF >0.05, the remaining markers were stored in data set SBCC_9K_LFMM.imputed.tr.tsv (*n* = 6,128). The program was run five times with 50 K cycles, and the same number for the burn‐in period. Several sets of latent factors (‐*K*) were tested, from four (as this is the optimal number of subpopulations detected by structure) to eight. We determined that the optimum value, according to the profiles of the histograms of combined adjusted *p*‐values was *K* = 6. The correlation *z*‐scores obtained from the five independent runs were combined using the Fisher‐Stouffer approach, recommended by the authors, and a FDR threshold (*Q* = 0.01) was calculated for each variable.

### 2.6 Population differentiation

Genotypes were classified into genetic clusters using the admixture model of the software package structure v.2.3.4 (Falush, Stephens, & Pritchard, 2003). Data for 8,457 polymorphic SNPs were used to run structure six times, setting the number of populations (*K*) from 1 to 6. For each run, burn‐in time and replication numbers were set to 10,000 and 20,000 Monte Carlo Markov Chain (MCMC) iterations, respectively. Evanno's ∆*K* (Evanno, Regnaut, & Goudet, 2005), as implemented in Structure Harvester (Earl & vonHoldt, 2012), was used to estimate the optimal number of subpopulations (Supporting Information Figure S13). Then, the program was run one more time using a burn‐in period of 100,000 and 100,000 MCMC iterations to estimate membership probability. Additionally, genotypes were classified using factorial analysis with darwin 6.0.4 (Perrier & Jacquemoud‐Collet, 2006), producing similar results as structure (Supporting Information Figure S14).

**Figure 2:**
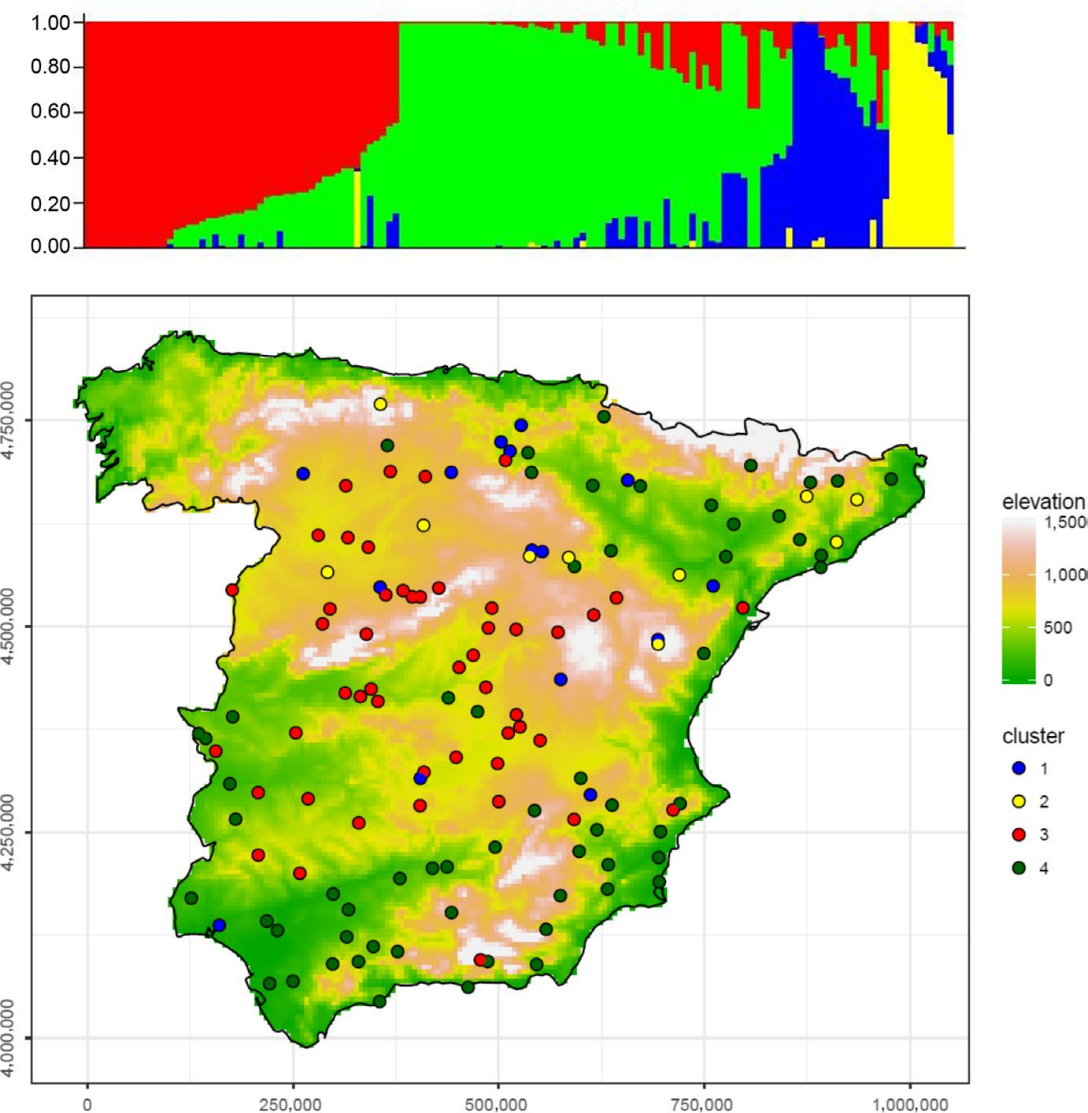
Top: group membership probabilities resulting from the Structure analysis run with a 100 K burn‐in and 100 K MCMC iterations, for *K* = 4 subpopulations. Cluster 1, blue, 6‐rowed barleys closer to European cultivars; cluster 2, yellow, 2‐rowed barleys; cluster 3, red, 6‐rowed barleys; cluster 4, green, six‐rowed barleys. Bottom: geographic distribution of the four subpopulations over elevation in mainland Spain (colour coded as in the top graph), represented in a UTM‐30N projection, axes in metres

Following the population differentiation analyses (seeSupporting Information File 1: SBCC_landraces), landraces were allocated to four clusters (Figure 2, Supporting Information Figures S13 and S14). Expected heterozygosity per locus (H) and differentiation between populations (Fst) per marker were calculated with arlequin 3.5 (Excoffier & Lischer, 2010). bayenv2 and baypass v2.1 (Gautier, 2015) were used to compute XtX, a statistic analogous to Fst that can identify loci that are more differentiated than expected under pure drift among populations (Günther & Coop, 2013). XtX, is more robust than Fst regarding differences in population sizes and independence from underlying genetic structure, as it explicitly accounts for the covariance structure among populations’ allele frequencies (Günther & Coop, 2013). With bayenv2, three replicates were performed and the average XtX value taken for each SNP marker. The parameters were*: ‐X ‐t ‐i SBCC_9K_subpops.tsv ‐p 4 ‐m SBCC_nr_subpops_matrix_mean.txt ‐k 200000 ‐e envfile.dummy ‐n 1 ‐c *(Supporting Information Figure S15). baypass extends on the model used by bayenv2, generating a set of theoretically neutral SNPs to help in the inference of significance thresholds for XtX. For this purpose, markers with less than 10% missing data and MAF ≥0.05 from the 9920_SNPs_SBCC_50K.tsv file were selected (*n* = 8,457). Linkage disequilibrium between neighbouring markers was calculated in r using package ldcorsv (Mangin et al., 2012). We report r_S_^2^, which incorporates into the calculation the information about the origins of each individual, i.e., the values of the *Q* matrix produced by structure. This calculation corrects for biases induced by population structure. For each SNP, we calculated r_S_^2^ values with four SNPs to each side, and the average value is reported. Heterozygosities, XtX and r_S_^2^ are reported for each single SNP, and also in 4 cM wide sliding windows, calculated with a purpose‐made Perl script.

The association of population differentiation with agroclimatic variables was explored further using redundancy analysis. This technique is widely used to test whether the variation in one set of (independent) variables explains the variation in another set of (dependent) variables. We followed an approach similar to the one reported by Leamy et al. (2016). The genetic differences among the SBCC lines, assessed by the four vectors of probabilities of belonging to the four genetic groups (*Q*) identified by the structure analysis were considered as the dependent variables. The complete set of variables, or the reduced set of 17 selected agroclimatic variables (excluding latitude, longitude, altitude and the dummy variables) comprised the independent sets. An independent assessment of the impact of the environment and geography on genetic differentiation among the landraces was assessed by comparing two partial RDA models. One included (besides the matrix with the *Q* values) all 17 agroclimatic variables, and the other the three geographic variables (latitude, longitude, altitude), in each case adjusted for the other set. By comparing the two models, the common and independent contributions of agroclimatic and geographic effects to the distribution of the genetic groups could be estimated, following the same procedure performed by Lasky et al. (2015). RDA was performed with the vegan package in R (Dixon, 2003). We used a permutational anova‐like test on redundancy‐analysis fitted data (function anova.cca) to test the significance of the effect of agroclimatic and geographic variables on the distribution of the four genetic groups.

## 3 RESULTS

### 3.1 Germplasm groups

Four groups of accessions (comprising 15, 10, 48 and 62 individuals) were identified by structure analysis (Figure 2, Supporting Information Table S1). These groups, with minor variations, corresponded to the populations already identified by Yahiaoui et al. (2008) using SSR markers. Group 1 included 15 six‐rowed accessions, related to European winter and spring barleys; group 2 consisted of 10 two‐rowed barleys, rather close to spring European 2‐rowed types; groups 3 (48 accessions) and 4 (62 accessions), all six‐rowed types except one two‐rowed in group 3, were widely distributed over the entire Peninsula (Figure 2), predominantly in inland northern‐central regions (group 3) and southern‐coastal regions (group 4). The first two groups are closer to other European cultivars, whereas the last two are genetically more distant from European cultivars, as pointed out in Yahiaoui et al. (2008). Fst, a measure of the differences of allelic frequencies between populations, was calculated for the four germplasm groups (Table 2). Differentiation between groups was minimum between groups 3 and 4, and maximum between these and group 2 (2‐rowed accessions). The Fst values between group 1 and the rest were intermediate, indicating a central position between them. H, averaged over all SNPs was rather low at the three predominantly 6‐rowed groups (1, 3, 4), and higher at the 2‐rowed group (2).

**Table 2:** Measure of population differentiation (Fst) between the four barley germplasm groups identified (for codes, see text). Diagonal, average diversity values (heterozygosities)

	1	2	3	4
1	0.217	0.242	0.246	0.227
2		0.331	0.378	0.352
3			0.196	0.186
4				0.224

**Figure 3:**
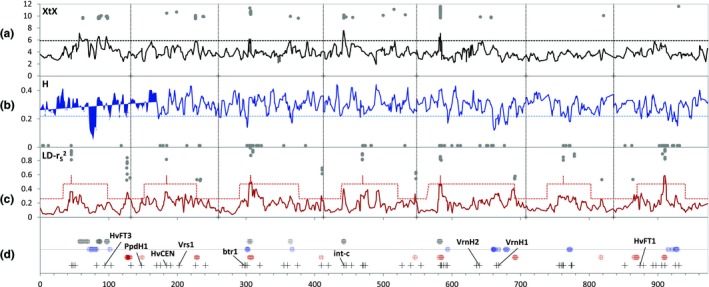
Genome wide diversity and genetic differentiation among germplasm groups of Spanish barleys, presented both as 4 cM sliding windows (lines), and as single SNPs (grey dots) with values beyond significance thresholds (XtX) or beyond the 1 (H) or 99 percentiles (LD). (a) 4 cM sliding windows for XtX values calculated for the four germplasm groups with baypass (solid black line), the black dashed line indicates the 95 percentile value for the 4 cM window scores; single SNP values above the baypass threshold, 9.56, are marked with grey dots; (b) heterozygosity (H) values for all accessions in 4 cM sliding windows (solid blue line), with a reference line drawn at percentile 5 (dashed blue line); single SNP values marked with grey dots indicate values below percentile 1; (c) linkage disequilibrium corrected for population structure (r_S_^2^) averaged for groups of nine contiguous SNPs, in 4 cM windows (solid red line), with a reference line (dashed red) drawn at the 95 percentile calculated separately at the three chromosomal zones described by Mascher et al. (2017), in which vertical spikes indicate the positions of centromeres, and single SNP values above percentile 99 are indicated with grey dots; (d) conceptual summary of graphs a–c, vertical scale offset, displaying positions of XtX and r_S_^2 ^sliding window values above percentile 95 and heterozygosity values below percentile 5. In graph (d), colour‐coded dots (enlarged for better visualization) correspond to positions in which 4 cM sliding windows values exceed those thresholds. Crosses at the bottom of this graph indicate the positions of well‐known flowering time and domestication genes, according to the updated popseq map (Beier et al., 2017), and listed in Supporting Information

Another measure of population differentiation, XtX, was used to search for patterns of genetic differentiation possibly related with the presence of selection footprints. baypass provided a significance threshold for XtX at 9.56 (i.e., XtX values above it indicate population differentiation above what could be expected for neutral markers). Heterozygosity and LD were examined in high XtX areas, to search for regions that hinted at the presence of selection footprints. The values for XtX calculated with baypass were lower in general than those calculated with bayenv2. As baypass values seem more conservative, and allow the calculation of a significance threshold, we will present only those. Moreover, the XtX scores calculated with both programs gave close results (*r* = 0.83, Supporting Information Figure S16). Peaks of the 4 cM sliding windows scan mark the regions with largest allelic differences across populations, indicating the most likely regions around genes that may have acted as drivers of differentiation between the groups (Figure 3). In barley, genes that govern growth cycle duration (known as flowering time genes), and spike‐type usually diverge among populations (Muñoz‐Amatriaín et al., 2014). Although it is no proof of association, it is worth noting that some of these genes fall within the main regions distinguishing the germplasm groups (Figure 3, Supporting Information Table S6). The rightmost XtX peak on 1HL (85.16–98.40 cM, 497–522 Mb) contains the *HvFT3 (PpdH2)* gene (514 Mb), among others. There were significant XtX values on the long arm of chromosome 2H. The most conspicuous, at 94 cM (226 cumulative cM, ccM), 697–700 Mb, also presented some high LD values (at 698 Mb), and low heterozygosity. Also in 2H, there was a cluster of high LD values around the position of gene *HvCEN* (51.81 cM, 184 ccM, 523 Mb, Supporting Information Tables S6, S7), accompanied by moderately high XtX values, although not significant. Chromosome 3H showed one of the main selection footprints, at 46.61–47.20 cM (306.3–306.9 ccM, 238–411 Mb in Figure 3, Supporting Information Figure S17, respectively) covered most of the pericentromeric region and part of both chromosomal arms, with high XtX, LD and heterozygosity values. This footprint was caused by the contrast between all four groups, except groups 3 and 4, which were quite similar (Supporting Information Figure S18a). A few more XtX significant values were present at the end of 3HL. On 4H, a clear XtX signal was visible at 29.72 cM, 16–19 Mb, coincident with the position of spike‐type gene *int‐c*, among other genes. The highest XtX peak was in chromosome 5H, in a very wide region (33.84–34.43 cM, 582.5–583.1 ccM, Figure 3, 72–348 Mb, Supporting Information Figure S17) containing flowering time gene *HvTFL1* (322 Mb, Supporting Information Table S6), caused by a sharp contrast between group 4 and the rest (Supporting Information Figure S18b). The highest XtX values together with high heterozygosity and LD values occurred flanking the centromere, and extending into both arms (Supporting Information Table S7, Figure S17). Other significant XtX values and high LD values were scattered on the distal part of the long arm.

### 3.2 Agroclimatic variables related to germplasm group differentiation

**Figure 4:**
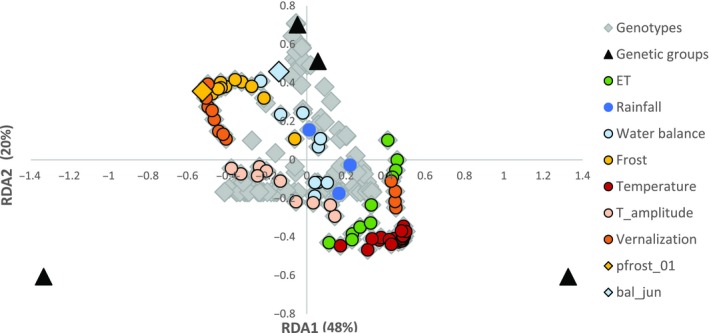
Triplot of the first two axes of a redundancy analysis with 103 agroclimatic variables (geographic variables longitude, latitude and altitude removed), showing genotypes, variables and germplasm groups. Variables are colour‐coded according to each category. The two variables which explained most variance in a multiple regression analysis are indicated with diamond icons

The redundancy analysis calculated with all the agroclimatic variables distributed the four genetic groups in a triangle shape (Figure 4) over the triplot representing the first two axes. The first axis separated the two main six‐rowed groups (3 and 4), and was related to temperature variables, with colder places on the left and warmer places on the right. The second axis separated groups 3 and 4 from groups 1 and 2. This axis was related to water availability variables, with groups 1 and 2 occurring in regions that are more humid. The analysis with the reduced (17) set of agroclimatic variables produced a very similar result (available in https://eead-csic-compbio.github.io/barley-agroclimatic-association/HOWTORDA.html). The whole set of agroclimatic variables explained close to 80% of the distribution of the germplasm groups, although this result was not significant, given the high number of variables involved. The reduced set of 17 variables explained a significant 37% (28% adjusted *R* ^2^, *p* < 0.001) of the distribution of genetic groups, of which 22% (21% adjusted *R* ^2^) was in common with spatial (or geographic) variables (latitude, longitude, altitude), and 7% adjusted *R* ^2^, still significant (*p* = 0.005), was unique (Supporting Information Figure S19). The spatial variables explained no unique variance after including the agroclimatic variables in the model. The most significant variables were selected via multiple regression. Only two variables entered into the model before the first dummy variable. These variables were pfrost and bal_jun, the first one related to temperature, the second one to water availability during the grain filling period. Together, they explained 24% of the genetic groups’ distribution, with a 4% of unique variance, still significant (*p* = 0.002). Spatial variables explained uniquely a nonsignificant (*p* = 0.058) 1.6% of the variance (Supporting Information Figure S19).

In a different and complementary approach, we ran a bayenv2 analysis for agroclimatic variables without taking into account population structure (null model). Under this model (Table 3), we found 905 marker‐variable associations above the selected BF and rho thresholds (top 1% for both parameters). Overall, variables related to frost showed the largest number of associations, followed by variables related to geography (longitude, latitude, altitude), vernalization and, variables related to water availability (Table 3, Supporting Information Figure S20). The sum of number of frost days in the months of January and February (frost_jan_feb), and the Julian date in which the probability of frost becomes lower than 10% (pfrost) were clearly the two variables with the highest number of SNPs associated. We are aware that many of these associations are false positives, caused by population structure, but the main purpose of this part of the study is to find the environmental drivers of population differentiation. The history of cultivation of barley in Spain points at climate adaptation, more than adaptation to different agricultural systems, as drivers of its geographic distribution, because most Spanish barleys were predominantly sown in autumn, in dryland conditions.

**Table 3:** Relationship of agroclimatic variables with molecular markers. Number of SNPs associated to agroclimatic variables above the combined BF and rho threshold (see full explanation in the Materials and Methods section) for the bayenv2 null model. Also shown, Pearson correlation coefficients between BF and genetic differentiation between the four germplasm groups (XtX, calculated with baypass). The standard deviation for the correlation coefficients with the 12 dummy variables is also shown

Variables	#Associations	r BF‐XtX
dummies (12)	5	0.027 ± 0.08
alt	74	0.256
bal_aut	0	–0.066
bal_jun	34	0.114
bal_mar_apr_may	0	0.180
bal_win	3	–0.051
ET_0__spr	12	0.264
frost_apr_may	11	0.238
frost_jan_feb	193	0.192
pfrost	233	0.295
lat	70	0.103
lon	78	0.015
pcp_aut	9	−0.018
pcp_mar_apr	0	−0.061
pcp_may_jun	17	0.145
pcp_win	11	−0.023
tamp_spr	0	0.071
tamp_win	0	0.033
verna_30d	40	0.268
verna_jan_feb	85	0.299
verna_mar_apr	30	0.263

To gain more insight in this direction, we compared population differentiation (XtX) values at SNP level with the BF resulting from the bayenv2 null analyses. The expectation was that the correlation between these two sets of variables would highlight the environmental traits most likely related with genetic group distribution. Pearson correlations between BF and XtX values were low overall, due to the occurrence of large number of SNPs with BF close to zero. The correlation coefficients for 12 dummy variables provided a baseline for comparison, varying between −0.07 and 0.20. For the null model (Table 3), nonzero correlation scores were mostly driven by coincidences of high BF values with some XtX peaks, at the same genomic regions for several agroclimatic variables. This was partially expected, due to collinearity between agroclimatic variables. Correlation coefficients between BF and XtX had values clearly above the dummy variables average, mostly for variables related to temperature (vernalization, frost) and water availability. It is remarkable that latitude and longitude, which presented a large number of SNPs with large BF, were not much more related to XtX than the set of dummy variables and, therefore, were probably not related to population differentiation.

**Figure 5:**
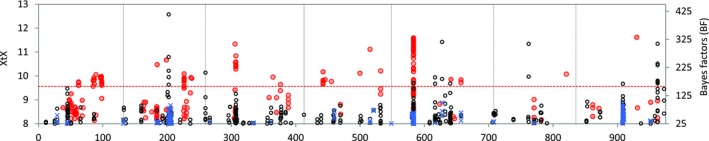
Association between XtX scores (population differentiation) and BF for selected agroclimatic variables. Red circles indicate XtX scores among germplasm groups (only values above eight are shown for better visualization); the dashed red line establishes the XtX significance threshold, at 9.56. Black circles indicate BF (only values above 25, for better visualization) for three frost agroclimatic variables, and three vernalization variables (blue times signs)

There was coincidence of position between some XtX peaks and accumulation of large BFs for agroclimatic variables, particularly for frost, which were the only variables with BF values over 100 (Figure 5), and vernalization. Some regions presented markers with large BF scores for frost variables, at cM 70 on 2H (202.5 ccM), and at 616, 627, 762, 908, and 962 ccM, but did not show significant XtX values. Two regions, however, presented the largest number of markers with large BF values for frost variables, and the highest number of significant XtX values, on 3H (47 cM, 307 ccM), and 5H (34 cM, 583 ccM), indicating the highest probability for harbouring genes relevant for population differentiation due to a differential response to temperature. At 92 cM on 5H (640 cM), there was another coincidence of significant XtX with large BF for frost variables.

### 3.3 Genomic regions associated with distribution of agroclimatic variables

**Figure 6:**
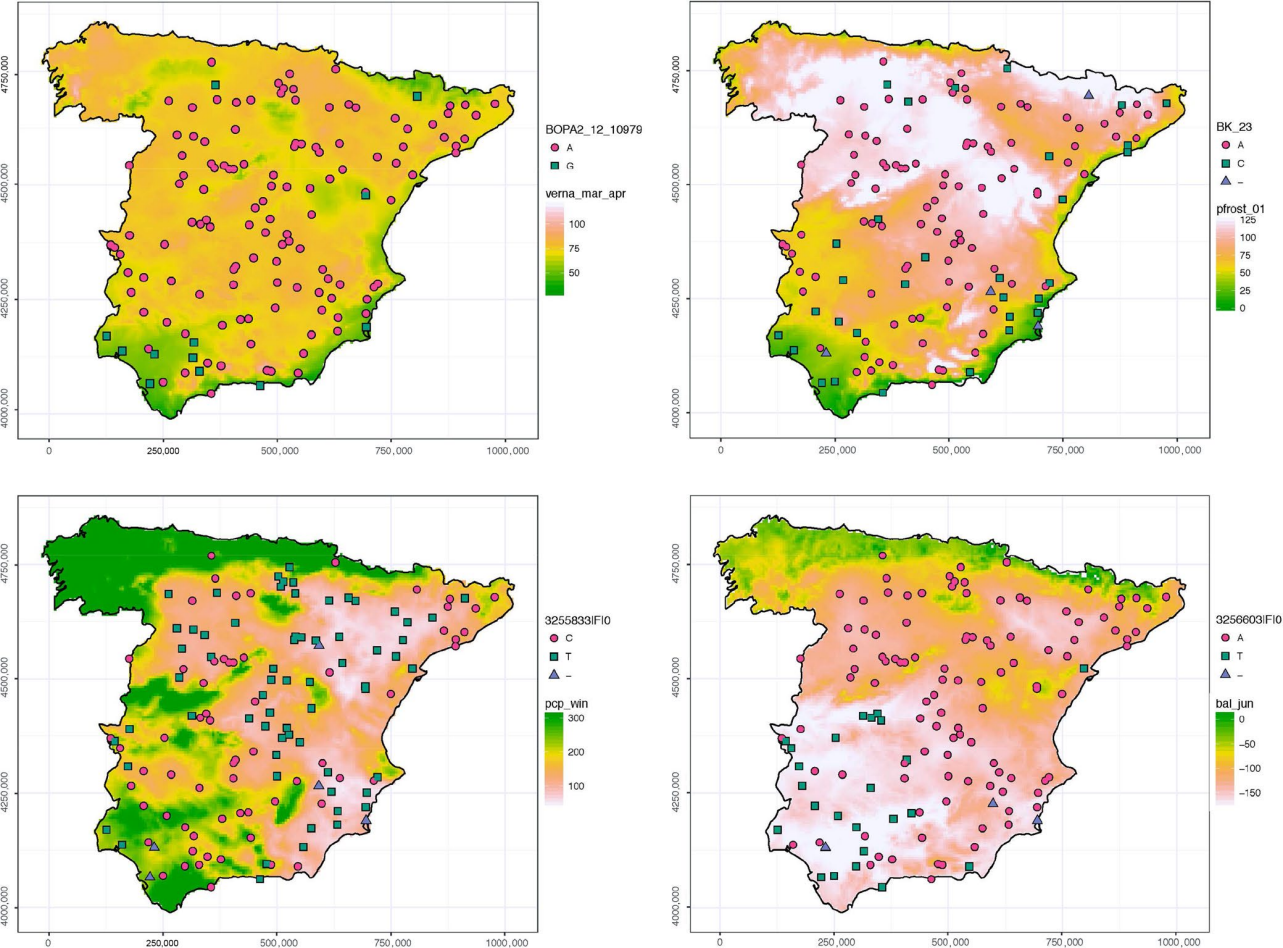
Maps of four agroclimatic variables related to vernalization (verna_mar_apr), late frost hazard (pfrost), hydric status (pcp_win) and drought (bal_jun). Four SNPs are shown as squares, circles and triangles placed in the original locations where landraces were collected

The bayenv2 covariance model and lfmm analyses found fewer associations than the null bayenv2 model, as expected (Supporting Information Figure S21), and more associations with latitude and longitude than with any other variable (Supporting Information Table S8). There was good agreement overall between the results of the two analyses. Correlation coefficients between the BFs and the −log of the *p*‐values produced by lfmm varied between 0.48 and 0.73 for each variable (average of 0.63). In these analyses, removing population covariance also entailed removing associations of the agroclimatic variables most related to germplasm group adaptation, to a larger extent than for geographic variables, particularly longitude and latitude. Associations of agroclimatic variables were reduced from 673 (Table 3) to 57 (Table 4), whereas associations with geographic variables fell from 232 (Table 3) to 54 (Supporting Information Table S8). There was only one association per PC, meaning that most of the variation explained by PCs was removed together with population structure. This fact further supports that population differentiation in Spanish barleys related to agroclimatic variables and adaptation more than to spatial divergence. In these analyses, frost‐, vernalization‐ and water‐related variables appeared related to a similar degree with genomic regions (Table 4). Thirty‐six SNPs presented 57 associations to agroclimatic variables, using the stringent bayenv2 and lfmm thresholds according to the threshold combining BF and rho values. An even more stringent threshold was calculated for the bayenv analyses, by taking as threshold the 99.99 percentile of the distribution of BF scores for the 12 dummy variables. This BF score was close to 10, coinciding with the lower limit for a “strong” evidence indicated by Bayes factors, according to the scale of Jeffreys (1961). Markers presented in Table 4 were significant in at least one analysis, and were beyond percentile 99 for the test statistic score for the other one. Twenty‐three genomic regions were identified, 10 for variables related to water availability (including PC2 and PC3), 11 to temperature (frost, vernalization, temperature amplitude), and two to both. A stretch of 1 Mb of the barley genome, to each side of each significant SNP, was examined to search for potential candidate genes consistent with the associations detected (Table 4), using the application barleymap (Cantalapiedra et al., 2015). In all cases but one, LD decayed to background levels along the 1 Mb region. A variable number of high confidence genes per region was found. It is speculative to point at candidates without further experimental proof. It is remarkable, however, that the two SNPs with highest associations to frost_jan_feb at 127 cM on 7H fell within and adjacent to genes HORVU7Hr1G118240 and HORVU7Hr1G118260, respectively, which encode polyamine oxidases. It is noteworthy to mention that this region was uniquely related to frost variables, and nothing else. There were associations of markers BK_23 and BOPA1_2208‐279 with frost and vernalization variables (Figure 6). Also, two more markers in the same region were the only ones associated with altitude (which could be a surrogate for temperature). These two markers are within the well‐known cluster of cold acclimation *CBF* genes, specifically inside *CBF4*.

**Table 4:** Reference genome search for the markers with highest BF factors (bayenv) and lowest *p*‐values (lfmm) within each genomic region associated with an agroclimatic variable (other than longitude and latitude)

Marker	chr	cM	Position (bp)	Agroclimatic variable	BF bayenv	−log10(P) lfmm	Neighbour genes			Gene_hit (horvu)	Gene_description
							HC	LC	Un		
3255550|F|0	1H	114.08	533,885,730	pcp_may_jun	7.5	**5.62**	54	60	0	—	—
3255919|F|0	2H	0.6	1,839,535	frost_jan_feb	**13.2**	4.28	54	49	0	—	—
				pfrost	**11.7**	4.15					
3256699|F|0	2H	31.37	46,833,247	frost_jan_feb	**10.9**	3.77	31	16	2	2Hr1G018380	Protein WEAK CHLOROPLAST MOVEMENT UNDER BLUE LIGHT 1
				pcp_may_jun	5.5	**5.23**					
3261758|F|0	2H	31.37	46,833,484	pcp_may_jun	**14.3**	**5.25**	31	16	2	2Hr1G018380	Protein WEAK CHLOROPLAST MOVEMENT UNDER BLUE LIGHT 1
SCRI_RS_144592	2H	49.43	114,309,420	bal_jun	14.1	**6.50**	10	8	1	2Hr1G030810	F‐box only protein 13
3262943|F|0	2H	49.43	114,710,786	bal_jun	10.0	**7.28**	12	11	1	—	—
				ET_0__spr	2.6	**5.33**					
BOPA1_946‐2500	2H	49.43	115,322,116	bal_jun	**13.0**	**7.43**	11	15	1	2Hr1G030870	Sucrose synthase 4
				pcp_may_jun	7.2	**5.32**					
BOPA1_2601‐171	2H	49.43	118,999,843	bal_jun	**15.7**	4.73	14	13	1	2Hr1G031310	Protein TIFY 3B
BOPA1_ConsensusGBS0033‐1	2H	63.77	636,292,482	bal_jun	**11.2**	**5.74**	27	14	0	2Hr1G089020	Disease resistance protein
				pcp_may_jun	**9.3**	4.53					
				ET_0__spr	4.3	**5.19**					
BOPA1_1613‐291	2H	104.48	723,245,814	bal_mar_apr_may	25.1	**5.36**	36	28	0	2Hr1G111600	Adenine nucleotide alpha hydrolases‐like superfamily protein
				ET_0__spr	17.6	**6.51**					
3256997|F|0	2H	108.11	727,985,335	pcp_may_jun	**9.5**	2.40	37	26	0	—	—
3261860|F|0	2H	121.46	751,637,636	tamp_spr	**15.4**	**7.29**	79	26	6	2Hr1G121480	Unknown function
BOPA1_1283‐332	2H	121.46	751,886,865	tamp_spr	**35.6**	**7.06**	77	29	6	2Hr1G121990	Calreticulin 1b
SCRI_RS_123364	2H	127.47	763,961,511	frost_jan_feb	**10.5**	3.68	87	72	6	2Hr1G126610	Transportin 1
										2Hr1G126620	undescribed protein
SCRI_RS_186444	3H	22.58	27,751,234	verna_jan_feb	**13.9**	3.72	53	48	1	3Hr1G012800	Disease resistance protein (CC‐NBS‐LRR class) family
3260736|F|0	3H	32.86	32,635,613	ET_0__spr	23.4	**6.25**	33	27	0	3Hr1G014280	DNA/RNA‐binding protein KIN17
				PC1	6.0	**5.24**					
3255833|F|0^a^	3H	47.2	468,265,740	pcp_aut	**10.1**	4.25	15	9	0	3Hr1G061550	undescribed protein
				pcp_win	**18.1**	4.37					
BOPA1_1977‐1385	3H	47.2	469,771,904	pcp_win	**12.4**	4.40	12	10	0	3Hr1G061690	Protein DEHYDRATION‐INDUCED 19 homolog 3
SCRI_RS_220192	3H	58.51	535,469,596	pcp_win	**14.1**	3.21	20	13	0	3Hr1G070850	NAD‐dependent malic enzyme 2
										3Hr1G070860	Protein cereblon
SCRI_RS_225540	3H	58.51	536,074,314	pcp_win	**9.4**	4.10	13	10	0	3Hr1G070960	Golgin−84
BOPA2_12_30399	3H	59.11	544,458,340	bal_win	7.2	**5.98**	21	36	1	3Hr1G072270	Coffea canephora DH200 = 94 genomic scaffold, scaffold_93
				pcp_win	3.6	**4.61**					
				PC3	5.6	**5.50**					
BOPA1_4025‐300	3H	97.75	624,282,646	bal_jun	**21.1**	4.33	37	18	2	3Hr1G088270	Cathepsin B‐like cysteine proteinase
BOPA1_6841‐637	4H	89.39	599,525,496	frost_jan_feb	**20.5**	2.75	41	38	1	4Hr1G075950	Ubiquitin‐fold modifier‐conjugating enzyme 1
3256603|F|0^a^	5H	59.53	529,391,379	bal_jun	**23.8**	2.58	24	19	0	—	—
BOPA1_2208‐279	5H	87.29	560,732,601	verna_30d	6.4	**5.72**	20	11	0	5Hr1G080450	C‐repeat‐binding factor 4
				verna_jan_feb	6.9	**5.38**					
				verna_mar_apr	5.8	**5.67**					
BK_23^a^	5H	87.29	560,732,648	pfrost	7.6	**5.26**	20	11	0	5Hr1G080450	C‐repeat‐binding factor 4
				verna_30d	8.7	**5.71**					
				verna_jan_feb	7.2	**5.38**					
				verna_mar_apr	7.7	**5.68**					
BOPA1_1628‐410	6H	23.49	16,749,790	frost_apr_may	2.8	**4.44**	75	56	1	6Hr1G009400	60S ribosomal protein L13−1
3254663|F|0	6H	54.48	396,127,093	frost_jan_feb	**30.2**	4.23	24	13	0	6Hr1G059780	F‐box/RNI‐like superfamily protein
SCRI_RS_226361	7H	2.41	5,168,993	bal_aut	**13.3**	**4.66**	64	57	1	7Hr1G002810	RNase H family protein
				pcp_aut	**12.3**	3.92					
				PC2	**36.3**	**4.83**					
BOPA2_12_10979^a^	7H	43.38	48,001,348	verna_30d	**12.8**	2.71	28	32	0	7Hr1G027120	Isoprenyl transferase
				verna_mar_apr	**12.8**	2.93					
SCRI_RS_126437	7H	116.35	638,089,288	verna_30d	**83.1**	5.15	28	45	1	7Hr1G113760	Transcription initiation factor TFIID subunit 5
				verna_mar_apr	**80.2**	5.04					
				verna_jan_feb	93.8	**6.39**					
BOPA2_12_31241	7H	116.35	638,447,284	verna_jan_feb	44.4	**5.48**	27	46	1	—	—
SCRI_RS_179554	7H	116.35	638,449,816	verna_jan_feb	35.7	**5.40**	26	46	1	—	—
BOPA1_8758‐564	7H	120.53	642,239,530	pcp_aut	**16.4**	5.68	61	38	0	7Hr1G115780	30S ribosomal protein S5
BOPA1_5595‐297	7H	127.15	647,000,345	frost_jan_feb	**21.2**	4.93	45	32	0	7Hr1G118230	Protein MEMO1
3257624|F|0	7H	127.15	647,041,453	frost_jan_feb	**16.8**	5.11	47	31	0	7Hr1G118240	Polyamine oxidase 1

Note

Highlighted, significant values with the stringent thresholds for each method. Markers reported had significant values for at least one of the analyses, and were beyond percentile 99 for the distribution of the other. The search encompassed a window of 1 Mb at each side of each marker. The number of neighbour gene models found in these windows is broken down into number of high confidence, low confidence and unclassified genes. Gene hits are provided when the SNP falls within a gene. Accessions of gene models and descriptions are provided in the two rightmost columns.

a The geographic distributions of these markers are presented in Figure 6.

## 4 DISCUSSION

Recent studies have attempted to reveal associations between environmental traits and genetic polymorphisms. Although the statistical techniques used were developed with a focus on natural populations (Günther & Coop, 2013; Günther, Lampei, Barilar, & Schmid, 2016), they are useful to detect meaningful associations in crops as well, using landraces (Abebe, Naz, & Léon, 2015; Lasky et al., 2015; Russell et al., 2016; Zhong et al., 2017). These studies, and the present one, are a case in point for the usefulness of EAA approaches to study crop adaptation. The scarcity of studies in this area could be due to the lack of collections of germplasm with appropriate geographic coverage, reliable passport data, and undisputed genetic lineage. The SBCC is an excellent material in this respect. The apparently limited geographic scope of the collection is compensated by the wide range of climatic conditions occurring in the Iberian Peninsula, and the wide genetic diversity at play.

The climate variables were derived from an extremely fine grid of weather stations and for a period of 30 years. This density of data is almost unprecedented in this kind of studies, and allows for a precise estimation of relevant agroclimatic variables at the places of collection of the accessions. The use of daily data, instead of more common monthly or seasonal values, allowed for the computation of relevant agroclimatic indices that cannot be derived from coarser data. These include variables highly relevant in the seasonal development of barley, such as vernalization indices or variables related to the risk of frost occurrence.

In the null model analysis, i.e., without considering genetic covariance among individuals (population structure), the associations found point to genomic regions truly related to adaptation to agroclimatic variables, but also detect false positives. The patterns of relationships observed, however, offer insights on which agroclimatic features were the main drivers of genetic differentiation of these barley germplasm groups. The highest number of associations were related to temperature, followed by water‐related variables. This result indicates the paramount importance of temperature adaptation in the dissemination of germplasm groups arriving and settling in the Iberian Peninsula. Variables related to the frequency of frosts, both in the winter and early spring, gave the highest associations to genetic markers which, simultaneously, presented the highest relationship with population differentiation, followed by variables describing vernalization potential. Therefore, winter temperatures and, to a lesser extent, water availability, played main roles in the distribution of barley germplasm groups arriving in the Iberian Peninsula.

Barley cultivars (like wheat) are roughly divided in spring and winter types. Winter barleys must combine the presence of gene *VrnH2* with an appropriate *VrnH1* allele (von Zitzewitz et al., 2005), to induce a vernalization requirement. Previously, we found a different *VrnH1 *allele in each of the two largest germplasm groups of Spanish barleys (Casao, Igartua, et al., 2011). Eighty four percent of Spanish landraces are winter‐types, with a vernalization requirement (Casao, Karsai, et al., 2011), that differs according to the *VrnH1* allele present (Casao, Karsai, et al., 2011). Therefore, a role of *VrnH1* as the driver of genetic group differentiation related to winter temperatures was expected. However, we did not find signals of population differentiation or association to agroclimatic variables around this gene (Figure 3). The only distinctive feature was the presence of some of the lowest values for H across the genome. This would be consistent with the existence of selection pressure towards winter alleles in *VrnH1* that could predate group differentiation.

From these analyses, it is not realistic to pinpoint the exact location of the genes responsible for these adaptations of the germplasm groups. It is also worth mentioning that geographical features like latitude and longitude presented a high number of associations with agroclimatic variables but, unlike frost and vernalization variables, had very low to negligible correlations with population differentiation. This distinction is important. Genetic diversity of landraces is expected to show some degree of spatial autocorrelation due to population movements and gene flow. If initial arrivals of the crop, or later movements within the country followed roughly East–West or North–South directions, associations of some markers with latitude and longitude are not surprising. Nevertheless, the fact that these associations were poorly related with population differentiation means that they bear little meaning as drivers of population adaptation.

In the models including population covariance, there were similar number of significant associations with variables related to water availability and temperature (Table 4), besides another 22 related to longitude, latitude, and altitude. This indicates that the evolution that occurred locally, once the different groups arrived in the Peninsula, was influenced as much by low temperature‐related variables as by water availability variables. Therefore, this germplasm is a potential source of genes and alleles for adaptation to water and temperature features. Barley first arrived in the area around 7,500 YBP (Zapata, Peña‐Chocarro, Pérez‐Jordá, & Stika, 2004), meaning that there has been enough time for admixture, local evolution and adaptation of barley. The fact that barley is essentially self‐fertilizing does not rule out the relevance of hybridization as a factor to promote gene shuffling. Most experimental evidence of outcrossing rates in cultivated barley yields estimates of around 1%, depending on floral morphology and environmental conditions (Abdel‐Ghani, Parzies, Omary, & Geiger, 2004, and references therein). Indeed, there is enough evidence confirming that hybridization events have been important in the evolution of many crops, even self‐fertilizing ones (Fuller et al., 2011). Intermediate *Q* values (probabilities of memberships to germplasm groups) of some or the barley accessions (Supporting Information Table S1) strongly indicate the occurrence of partial admixture in Spanish barleys.

Two regions stood out as harbouring the most SNPs showing signs of recent selection footprints. Both regions, on 3H (238–411 Mb) and 5H (72–348 Mb) were narrow in genetic distance, but large in physical distance. The region on 5H apparently overlaps with one of the regions highlighted by Fang et al. (2014) as the most important for population differentiation and environmental adaptation in wild barley. The regions on 5H are actually not the same in both studies. As stated by Fang et al. (2014), their 5H region goes from 47 to 52 cM, in their map, pointing at markers that correspond to 371–448 Mb in the current reference genome, and is located to the right of the centromere, according to the sequence published by Mascher et al. (2017). Therefore, it falls just to the right of our high XtX region. However, looking closer at the results of Fang et al. (2014), their high Fst region on 5H includes another three markers with high Fst values, located at 38.78–41.45 cM (50–107 Mb in the current genome). This last region, to the left of the centromere, overlaps with our high XtX region. The maximum XtX values of the whole genome are located between 72 and 348 Mb, coinciding with some of the largest BF values for frost variables, between 66–354 Mb. This region on 5H actually covers about 41% of the chromosome in physical distance and, under closer inspection (Supporting Information Table S7), two regions are visible, one to each side of the proximal/pericentromeric region. Regarding the other region highlighted by Fang et al. (2014) on 2H, it spanned from 427 to 550 Mb, according to the current reference genome. In that area, there was a high LD signal in our data, coincident with moderately high XtX values, but below the significance threshold (Supporting Information Table S7). One possibility to explain the occurrence of these high LD regions differing among populations would be a suppressed recombination due to inversions that capture locally adapted alleles when two populations are hybridizing (Kirkpatrick & Barton, 2006).

A possible picture, compatible with all these previous data, is the occurrence of genetic differentiation driven by environmental adaptation of barley stocks moving along barley paths of distribution. The study by Muñoz‐Amatriaín et al. (2014) detected high genetic differentiation among barley germplasm groups, representing the whole world, at the 5H region mentioned in the previous paragraph. In this region of 5H, there is co‐location of population differentiation and association of markers to agroclimatic variables. We cannot conclude that these two facts are causally related, due to the aforementioned lack of protection against false positives. The commonalities found, however, are striking, and confirm the interest of this genomic region for further research.

The number of SNPs associated to agroclimatic variables was low, only 36, i.e., 0.5% of all SNPs with position. This low number is probably caused by the stringent statistical thresholds used, and is in line with the expectations that only a small portion of the genome will be associated with adaptation to climate (Meirmans, 2015). An examination of the genes present in the reference genome near the markers significant under the population covariance model found a high number of gene models, from which it is difficult to pinpoint potential candidates. A few of the genes harbouring SNPs shown in Table 4 had also prior information linking them to effects related to responses to the agroclimatic variables associated. These genes will be discussed in more detail.

Markers, BOPA1_946‐2500 and BOPA1_1977‐1385, associated to water availability variables, occur within genes that could be linked to stress responses, such as sucrose synthase 4 and Protein dehydration‐induced 19 homolog. Marker BOPA2_12_10979 corresponds to a gene coding for an isoprenyl transferase, and was associated to variables describing vernalization potential. This kind of gene has been connected to water stress responses (Pei, Ghassemian, Kwak, McCourt, & Schroeder, 1998) and oxidative stress in plants (Grassmann, Hippeli, & Elstner, 2002). Marker BOPA1_4025‐300 occurs within a gene coding for a cathepsin B‐like cysteine proteinase. These enzymes have been reported to increase their expression in barley in response to cold treatment (Martínez, Rubio‐Somoza, Carbonero, & Díaz, 2003), and to provide frost protection in wheat (Talanova, Titov, Topchieva, & Frolova, 2012), although the association in our study occurred with a variable related to water availability.

Two marker‐agroclimatic associations deserve further comment. A distal region on the long arm of 7H, around 127 cM, appeared related to frost variables under both the null and the covariance models. The marker with the largest BF for number of days of frost in January–February was actually within a gene coding for a polyamine oxidase. Polyamines have been frequently reported as part of the response of *Arabidopsis thaliana* to abiotic stresses and, in particular, to cold tolerance (Cuevas et al., 2008). The diverse roles of several polyamines in response to cold stress in crop species, particularly in winter cereals, was revealed in a number of studies (reviewed by Pecchioni et al., 2014). Wang, Dinler, Vignjevic, Jacobsen, and Wollenweber (2015) also found a role for polyamines in wheat, not only in cold stress responses, but also in heat stress responses, as already pointed out previously by Goyal and Asthir (2010). Further experimental evidence is needed to confirm whether or not they have any role in barley adaptation.

Finally, BOPA1_2208‐279 and BK_23 also fall within a potential candidate gene. These markers are placed within *CBF4*, the last of the well‐known cluster of *CBF* genes on chromosome 5H, identified as the responsible for the most important frost tolerance QTL in barley, *FrH2* (Francia et al., 2007, 2016). Additionally, markers 3258527 and SCRI_RS_224251 were the only ones associated with altitude, which could be a proxy for frost variables. They are located just 600 bp and 7 Mb away from *CBF4*, so they probably are part of the same signal. *CBF*s and their regulon are major determinants of low‐temperature tolerance. CBFs are transcription factors that regulate suites of genes during drought and low temperature stresses. Their evolution was involved in Pooideae adaptation to cold climates (Sandve & Fjellheim, 2010). Specifically, members of the CBF3/4‐subfamilies are thought to play roles in Pooideae adaptation to freezing stress (Li et al., 2012). Their role in frost tolerance has been related to different temperatures of threshold induction, different expression levels (Galiba, Vágújfalvi, Li, Soltész, & Dubcovsky, 2009; Knox et al., 2010) and to copy number variation (Francia et al., 2016; Tondelli, Francia, Barabaschi, Pasquariello, & Pecchioni, 2011). CNV occurrence is highly dynamic at this locus and, therefore, it is not surprising that it appears linked to altitude and frost occurrence in a collection of landrace germplasm. The identification of associations of markers in *CBF4* as associated to vernalization and frost variables is a case in point that validates the EAA strategy followed.

These findings reveal new information about the environmental drivers of genetic diversification of barley. Winter temperatures, affecting frost occurrence and vernalization potential, are behind the genetic differences between the groups of Spanish barley landraces. The relevance of this conclusion exceeds the local interest, as the Iberian Peninsula is the endpoint of the routes of Neolithic expansion that encompassed the entire Mediterranean basin. In some cases, we were able to pinpoint new candidate genes associated to specific environmental conditions that open further avenues for research on cereal crops adaptation. Given the genetic closeness of wheat and barley, and the parallel history of expansion of these crops, the occurrence of similar processes in wheat deserves investigation. This knowledge, after further experimental validation and allele mining, will be applicable to prebreeding and breeding of barley.

## AUTHOR CONTRIBUTION

E.I., A.M.C., S.B., B.C.M., designed the study. RSN, SB, collected the climatic data, prepared the climate database, and derived the agroclimatic variables. A.M.C., N.E.M., carried out the genetic diversity analyses. B.C.M., C.P.C., performed the Bayenv, LFMM and baypass analyses. All authors performed statistical analyses linking geographic and genetic data. A.M.C., C.P.C., B.C.M., curated the genotypic data and prepared genotypic databases. A.M.C. secured funding. A.M.C., E.I., R.S.N., S.B., B.C.M., wrote the manuscript. All authors edited and approved the manuscript.

## DATA AVAILABILITY

Climate data, genotype and marker information are provided in the supplementary excel file. All the data files and scripts used for the selection of climate variables and the association analyses are described in R markdown documents, available at https://eead-csic-compbio.github.io/barley-agroclimatic-association, https://doi.org/10.5281/zenodo.1886991.

## References

[mec15009-bib-0001] Abdel‐Ghani, A. H. , Parzies, H. K. , Omary, A. , & Geiger, H. H. (2004). Estimating the outcrossing rate of barley landraces and wild barley populations collected from ecologically different regions of Jordan. Theoretical and Applied Genetics, 109, 588–595. 10.1007/s00122-004-1657-1 15083273

[mec15009-bib-0002] Abebe, T. D. , Naz, A. A. , & Léon, J. (2015). Landscape genomics reveal signatures of local adaptation in barley (*Hordeum vulgare* L.). Frontiers Plant Science, 6, 813 10.3389/fpls.2015.00813 PMC4591487 26483825

[mec15009-bib-0003] Allen, R. G. , Pereira, L. S. , Raes, D. , & Smith, M. (1998). Crop evapotranspiration – Guidelines for computing crop water requirements – FAO irrigation and drainage paper 56. Rome, Italy: FAO. ISBN 92‐5‐104219‐5.

[mec15009-bib-0004] Beguería, S. (2010). Generating spatially correlated random fields with r. Retrieved from http://santiago.begueria.es/2010/10/generating-spatially-correlated-random-fields-with-r/

[mec15009-bib-0005] Beguería, S. , & Pueyo, Y. (2009). A comparison of simultaneous autoregressive and generalized least squares models for dealing with spatial autocorrelation. Global Ecology and Biogeography, 18, 273–279. 10.1111/j.1466-8238.2009.00446.x

[mec15009-bib-0006] Beier, S. , Himmelbach, A. , Colmsee, C. , Zhang, X.‐Q. , Barrero, R. A. , Zhang, Q. , … Mascher, M. (2017). Construction of a map‐based reference genome sequence for barley, *Hordeum vulgare* L. Scientific Data, 4, 170044 10.1038/sdata.2017.44 28448065 PMC5407242

[mec15009-bib-0007] Cantalapiedra, C. P. , Boudiar, R. , Casas, A. M. , Igartua, E. , & Contreras‐Moreira, B. (2015). BARLEYMAP: Physical and genetic mapping of nucleotide sequences and annotation of surrounding loci in barley. Molecular Breeding, 35, 13 10.1007/s11032-015-0253-1

[mec15009-bib-0008] Casao, M. C. , Igartua, E. , Karsai, I. , Lasa, J. M. , Gracia, M. P. , & Casas, A. M. (2011). Expression analysis of vernalization and day‐length response genes in barley (*Hordeum vulgare *L.) indicates that *VRNH2* is a repressor of *PPDH2* (*HvFT3*) under long days. Journal of Experimental Botany, 62, 1939–1949. 10.1093/jxb/erq382 21131547 PMC3060678

[mec15009-bib-0009] Casao, M. C. , Karsai, I. , Igartua, E. , Gracia, M. P. , Veisz, O. , & Casas, A. M. (2011). Adaptation of barley to mild winters: A role for PPDH2. BMC Plant Biology, 11, 164 10.1186/1471-2229-11-164 22098798 PMC3226555

[mec15009-bib-0010] Casas, A. M. , Contreras‐Moreira, B. , Cantalapiedra, C. P. , Sakuma, S. , Gracia, M. P. , Moralejo, M. , … Igartua, E. (2018). Resequencing the *Vrs1* gene in Spanish barley landraces revealed reversion of six‐rowed to two‐rowed spike. Molecular Breeding, 38, 51 10.1007/s11032-018-0816-z

[mec15009-bib-0011] Ciudad, F. J. (2002). Análisis y modelización de las respuestas a vernalización y fotoperiodo en cebada (Hordeum vulgare L.). PhD thesis, University of Valladolid. Retrieved from https://www.educacion.es/teseo/mostrarRef.do?ref=269529

[mec15009-bib-0012] Comadran, J. , Kilian, B. , Russell, J. , Ramsay, L. , Stein, N. , Ganal, M. , … Waugh, R. (2012). Natural variation in a homolog of *Antirrhinum CENTRORADIALIS* contributed to spring growth habit and environmental adaptation in cultivated barley. Nature Genetics, 44, 1388–1392. 10.1038/ng.2447 23160098

[mec15009-bib-0013] Cressie, N. A. C. (1993). Statistics for spatial data. Wiley series in probability and mathematical statistics (p. 900). New York, NY: John Wiley & Sons Inc..

[mec15009-bib-0014] Cuevas, J. C. , Lopez‐Cobollo, R. , Alcazar, R. , Zarza, X. , Koncz, C. , Altabella, T. , … Ferrando, A. (2008). Putrescine is involved in Arabidopsis freezing tolerance and cold acclimation by regulating abscisic acid levels in response to low temperature. Plant Physiology, 148, 1094–1105. 10.1104/pp.108.122945 18701673 PMC2556839

[mec15009-bib-0015] De Castro, M. J. , Martín‐Vide, M. , & Brunet, M. (2005). The climate of Spain: Past, present and scenarios for the 21st century In: Impacts of climatic change in spain (pp.207–218). A preliminary assessment of the impacts in Spain due to the effects of climate change. ECCE Project-Final report Ministerio de Medio Ambiente, Madrid, Spain Publicaciones Ministerio de Medio Ambiente.

[mec15009-bib-0016] Dixon, P. (2003). VEGAN, a package of R functions for community ecology. Journal of Vegetation Science, 14, 927–930. 10.1111/j.1654-1103.2003.tb02228.x

[mec15009-bib-0017] Earl, D. A. , & vonHoldt, B. M. (2012). STRUCTURE HARVESTER: A website and program for visualizing STRUCTURE output and implementing the Evanno method. Conservation Genetics Resources, 4, 359–361. 10.1007/s12686-011-9548-7

[mec15009-bib-0018] Evanno, G. , Regnaut, S. , & Goudet, J. (2005). Detecting the number of clusters of individuals using the software STRUCTURE: A simulation study. Molecular Ecology, 14, 2611–2620. 10.1111/j.1365-294X.2005.02553.x 15969739

[mec15009-bib-0019] Excoffier, L. , & Lischer, H. E. L. (2010). Arlequin suite ver 3.5: A new series of programs to perform population genetics analyses under Linux and Windows. Molecular Ecology Resources, 10, 564–567. 10.1111/j.1755-0998.2010.02847.x 21565059

[mec15009-bib-0020] Falush, D. , Stephens, M. , & Pritchard, J. K. (2003). Inference of population structure using multilocus genotype data: Linked loci and correlated allele frequencies. Genetics, 164, 1567–1587. 12930761 10.1093/genetics/164.4.1567 PMC1462648

[mec15009-bib-0021] Fang, Z. , Gonzales, A. M. , Clegg, M. T. , Smith, K. P. , Muehlbauer, G. J. , Steffenson, B. J. , & Morrell, P. L. (2014). Two genomic regions contribute disproportionately to geographic differentiation in wild barley. G3 (Bethesda), G3(4), 1193–1203. 10.1534/g3.114.010561 PMC4455769 24760390

[mec15009-bib-0022] Fischbeck, G. (2002). Contribution of barley to agriculture: A brief overview In Slafer G. A., Molina‐Cano J. L., Savin R., Araus J. L., & Romagosa I. (Eds.), Barley science. Recent advances from molecular biology to agronomy of yield and quality (pp. 1–14). New York, NY: Food Products Press.

[mec15009-bib-0023] Fournier‐Level, A. , Wilczek, A. M. , Cooper, M. D. , Roe, J. L. , Anderson, J. , Eaton, D. , … Schmitt, J. (2013). Paths to selection on life history loci in different natural environments across the native range of *Arabidopsis thaliana* . Molecular Ecology, 22, 3552–3566. 10.1111/mec.12285 23506537

[mec15009-bib-0024] Francia, E. , Barabaschi, D. , Tondelli, A. , Laidò, G. , Rizza, F. , Stanca, A. M. , … Pecchioni, N. (2007). Fine mapping of a *HvCBF* gene cluster at the frost resistance locus *Fr‐H2* in barley. Theoretical and Applied Genetics, 115, 1083–1091. 10.1007/s00122-007-0634-x 17763839

[mec15009-bib-0025] Francia, E. , Morcia, C. , Pasquariello, M. , Mazzamurro, V. , Milc, J. A. , Rizza, F. , … Pecchioni, N. (2016). Copy number variation at the *HvCBF4–HvCBF2* genomic segment is a major component of frost resistance in barley. Plant Molecular Biology, 92, 161–175. 10.1007/s11103-016-0505-4 27338258

[mec15009-bib-0026] Frichot, E. , Schoville, S. D. , Bouchard, G. , & François, O. (2013). Testing for associations between loci and environmental gradients using latent factor mixed models. Molecular Biology and Evolution, 30, 1687–1699. 10.1093/molbev/mst063 23543094 PMC3684853

[mec15009-bib-0027] Fuller, D. Q. , Willcox, G. , & Allaby, R. G. (2011). Cultivation and domestication had multiple origins: Arguments against the core area hypothesis for the origins of agriculture in the Near East. World Archaeology, 43, 628–652. 10.1080/00438243.2011.624747

[mec15009-bib-0028] Galiba, G. , Vágújfalvi, A. , Li, C. , Soltész, A. , & Dubcovsky, J. (2009). Regulatory genes involved in the determination of frost tolerance in temperate cereals. Plant Science, 176, 12–2012. 10.1016/j.plantsci.2008.09.016

[mec15009-bib-0029] Gautier, M. (2015). Genome‐wide scan for adaptive divergence and association with population‐specific covariates. Genetics, 201, 1555–1579. 10.1534/genetics.115.181453 26482796 PMC4676524

[mec15009-bib-0030] Goyal, M. , & Asthir, B. (2010). Polyamine catabolism influences antioxidative defense mechanism in shoots and roots of five wheat genotypes under high temperature stress. Plant Growth Regulation, 60, 13–25. 10.1007/s10725-009-9414-8

[mec15009-bib-0031] Grassmann, J. , Hippeli, S. , & Elstner, E. F. (2002). Plant’s defence and its benefits for animals and medicine: Role of phenolics and terpenoids in avoiding oxygen stress. Plant Physiology and Biochemistry, 40, 471–478. 10.1016/S0981-9428(02)01395-5

[mec15009-bib-0032] Günther, T. , & Coop, G. (2013). Robust identification of local adaptation from allele frequencies. Genetics, 195, 205–220. 10.1534/genetics.113.152462 23821598 PMC3761302

[mec15009-bib-0033] Günther, T. , Lampei, C. , Barilar, I. , & Schmid, K. J. (2016). Genomic and phenotypic differentiation of *Arabidopsis thaliana* along altitudinal gradients in the North Italian Alps. Molecular Ecology, 25, 3574–3592. 10.1111/mec.13705 27220345

[mec15009-bib-0035] Igartua, E. , Gracia, M. P. , Lasa, J. M. , Medina, B. , Molina‐Cano, J. L. , Montoya, J. L. , & Romagosa, I. (1998). The Spanish barley core collection. Genetic Resources and Crop Evolution, 45, 475–482. 10.1023/A:1008662515059

[mec15009-bib-0037] Jeffreys, H. (1961). Theory of probability, 3rd ed Oxford, UK: Oxford University Press, Clarendon Press.

[mec15009-bib-0038] Jones, H. , Lister, D. L. , Bower, M. A. , Leigh, F. J. , Smith, L. M. , & Jones, M. K. (2008). Approaches and constraints of using existing landrace and extant plant material to understand agricultural spread in prehistory. Plant Genetic Resources: Characterization and Utilization, 6, 98–112. 10.1017/S1479262108993138

[mec15009-bib-0039] Kilian, A. , Wenzl, P. , Huttner, E. , Carling, J. , Xia, L. , Blois, H. , … Uszynski, G. (2012). Diversity arrays technology: A generic genome profiling technology on open platforms InPompanon F. & Bonin A. (Eds.), Data production and analysis in population genomics. methods in molecular biology (Methods and Protocols), (vol. 888, pp. 67–89). Totowa, NJ: Humana Press. 10.1007/978-1-61779-870-2_5 22665276

[mec15009-bib-0040] Kirkpatrick, M. , & Barton, N. (2006). Chromosome inversions, local adaptation and speciation. Genetics, 173, 419–434. 10.1534/genetics.105.047985 16204214 PMC1461441

[mec15009-bib-0041] Knox, A. K. , Dhillon, T. , Cheng, H. , Tondelli, A. , Pecchioni, N. , & Stockinger, E. J. (2010). *CBF* gene copy number variation at *Frost Resistance‐2* Is associated with levels of freezing tolerance in temperate‐climate cereals. Theoretical and Applied Genetics, 121, 21–35. 10.1007/s00122-010-1288-7 20213518

[mec15009-bib-0042] Komatsuda, T. , Pourkheirandish, M. , He, C. , Azhaguvel, P. , Kanamori, H. , Perovic, D. , … Yano, M. (2007). Six‐rowed barley originated from a mutation in a homeodomain‐leucine zipper I‐class homeobox gene. Proceedings of the National Academy of Sciences of United States of America, 104, 1424–1429. 10.1073/pnas.0608580104 PMC1783110 17220272

[mec15009-bib-0043] Lasky, J. R. , Upadhyaya, H. D. , Ramu, P. , Deshpande, S. , Hash, C. T. , Bonnette, J. , … Morris, G. P. (2015). Genome‐environment associations in sorghum landraces predict adaptive traits. Science Advances, 1, e1400218 10.1126/sciadv.1400218 26601206 PMC4646766

[mec15009-bib-0044] Leamy, L. J. , Lee, C. R. , Song, Q. , Mujacic, I. , Luo, Y. , Chen, C. Y. , … Song, B. H. (2016). Environmental versus geographical effects on genomic variation in wild soybean (*Glycine soja*) across its native range in northeast Asia. Ecology and Evolution, 6, 6332–6344. 10.1002/ece3.2351 27648247 PMC5016653

[mec15009-bib-0045] Leinonen, P. H. , Remington, D. L. , Leppälä, J. , & Savolainen, O. (2013). Genetic basis of local adaptation and flowering time variation in *Arabidopsis lyrata* . Molecular Ecology, 22, 709–723. 10.1111/j.1365-294X.2012.05678.x 22724431

[mec15009-bib-0046] Li, C. , Rudi, H. , Stockinger, E. J. , Cheng, H. , Cao, M. , Fox, S. E. , … Sandve, S. R. (2012). Comparative analyses reveal potential uses of *Brachypodium distachyon* as a model for cold stress responses in temperate grasses. BMC Plant Biology, 12, 65 10.1186/1471-2229-12-65 22569006 PMC3487962

[mec15009-bib-0047] LP DAAC (1996). GTOPO30. NASA EOSDIS Land Processes DAAC, USGS Earth Resources Observation and Science (EROS) Center, Sioux Falls, South Dakota. Retrieved from https://lpdaac.usgs.gov

[mec15009-bib-0048] Mangin, B. , Siberchicot, A. , Nicolas, S. , Doligez, A. , This, P. , & Cierco‐Ayrolles, C. (2012). Novel measures of linkage disequilibrium that correct the bias due to population structure and relatedness. Heredity, 108, 285–291. 10.1038/hdy.2011.73 21878986 PMC3282397

[mec15009-bib-0049] Manzano‐Piedras, E. , Marcer, A. , Alonso‐Blanco, C. , & Picó, F. X. (2014). Deciphering the adjustment between environment and life history in annuals: Lessons from a geographically‐ explicit approach in *Arabidopsis thaliana* . PLoS ONE, 9, e87836 10.1371/journal.pone.0087836 24498381 PMC3912251

[mec15009-bib-0050] Martínez, M. , Rubio‐Somoza, I. , Carbonero, P. , & Díaz, I. (2003). A cathepsin B‐like cysteine protease gene from *Hordeum vulgare* (gene *CatB*) induced by GA in aleurone cells is under circadian control in leaves. Journal of Experimental Botany, 54, 951–959. 10.1093/jxb/erg099 12598566

[mec15009-bib-0051] Mascher, M. , Gundlach, H. , Himmelbach, A. , Beier, S. , Twardziok, S. O. , Wicker, T. , … Stein, N. (2017). A chromosome conformation capture ordered sequence of the barley genome. Nature, 544, 427–433. 10.1038/nature22043 28447635

[mec15009-bib-0052] Meirmans, P. G. (2015). Seven common mistakes in population genetics and how to avoid them. Molecular Ecology, 24, 3223–3231. 10.1111/mec.13243 25974103

[mec15009-bib-0053] Moragues, M. , García del Moral, L. F. , Moralejo, M. , & Royo, C. (2006a). Yield formation strategies of durum wheat landraces with distinct pattern of dispersal within the Mediterranean basin I: Yield components. Field Crops Research, 95, 194–205. 10.1016/j.fcr.2005.02.009

[mec15009-bib-0054] Moragues, M. , García del Moral, L. F. , Moralejo, M. , & Royo, C. (2006b). Yield formation strategies of durum wheat landraces with distinct pattern of dispersal within the Mediterranean basin: II. Biomass production and allocation. Field Crops Research, 95, 182–193. 10.1016/j.fcr.2005.02.008

[mec15009-bib-0055] Muñoz‐Amatriaín, M. , Cuesta‐Marcos, A. , Endelman, J. B. , Comadran, J. , Bonman, J. M. , Bockelman, H. E. , … Muehlbauer, G. J. (2014). The USDA barley core collection: Genetic diversity, population structure, and potential for genome‐wide association studies. PLoS ONE, 9, e94688 10.1371/journal.pone.0094688 24732668 PMC3986206

[mec15009-bib-0056] Murtagh, F. , & Legendre, P. (2014). Ward's hierarchical agglomerative clustering method: Which algorithms implement Ward's criterion? Journal of Classification, 31, 274–295. 10.1007/s00357-014-9161-z

[mec15009-bib-0057] Myers, N. , Mittermeier, R. , Mittermeier, C. , da Fonseca, G. , & Kent, J. (2000). Biodiversity hotspots for conservation priorities. Nature, 403, 853–858. 10.1038/35002501 10706275

[mec15009-bib-0058] Newton, A. C. , Akar, T. , Baresel, J. P. , Bebeli, P. J. , Bettencourt, E. , Bladenopoulos, K. V. , … Vaz Patto, M. C. (2010). Cereal landraces for sustainable agriculture. A review. Agronomy for Sustainable Development, 30, 237–269. 10.1051/agro/2009032

[mec15009-bib-0059] Pebesma, E. J. (2004). Multivariable geostatistics in S: The gstat package. Computers & Geosciences, 30, 683–691. 10.1016/j.cageo.2004.03.012

[mec15009-bib-0060] Pecchioni, N. , Kosová, K. , Vítámvás, P. , Prášil, I. T. , Milc, J. A. , Francia, E. , … Galiba, G. (2014). Genomics of low‐temperature tolerance for an increased sustainability of wheat and barley production In Tuberosa R., Graner A., Frison E. (Eds.), Genomics of Plant Genetic Resources (pp. 149–183). Dordrecht, The Netherlands: Springer 10.1007/978-94-007-7575-6_6

[mec15009-bib-0061] Pei, Z.‐M. , Ghassemian, M. , Kwak, C. M. , McCourt, P. , & Schroeder, J. I. (1998). Role of farnesyltransferase in ABA regulation of guard cell anion channels and plant water loss. Science, 282, 287–290. 10.1126/science.282.5387.287 9765153

[mec15009-bib-0062] Perrier, X. , & Jacquemoud‐Collet, J. P. (2006). DARwin software. Retrieved from http://darwin.cirad.fr/

[mec15009-bib-0064] R Core Team (2017). R: A language and environment for statistical computing. Vienna, Austria: R Foundation for Statistical Computing https://www.R-project.org/

[mec15009-bib-0065] Ritchie, J. T. (1991). Wheat phasic development In Hanks T., & Ritchie J. T. (Eds.), Modeling plant and soil systems (pp. 31–54). Agronomy monograph, 31. Madison, WI: ASA, CSSSA, SSSA.

[mec15009-bib-0066] Russell, J. , Mascher, M. , Dawson, I. K. , Kyriakidis, S. , Calixto, C. , Freund, F. , … Waugh, R. (2016). Exome sequencing of geographically diverse barley landraces and wild relatives gives insights into environmental adaptation. Nature Genetics, 48, 1024–1030. 10.1038/ng.3612 27428750

[mec15009-bib-0067] Sandve, S. R. , & Fjellheim, S. (2010). Did gene family expansions during the Eocene‐Oligocene boundary climate cooling play a role in Pooideae adaptation to cool climates? Molecular Ecology, 19, 2075–2088. 10.1111/j.1365-294X.2010.04629.x 20406386

[mec15009-bib-0068] Serrano‐Notivoli, R. , de Luis, M. , & Beguería, S. (2017). An R package for daily precipitation climate series reconstruction. Environmental Modelling and Software, 89, 190–195. 10.1016/j.envsoft.2016.11.005

[mec15009-bib-0069] Serrano‐Notivoli, R. , de Luis, M. , Saz, M. A. , & Beguería, S. (2017). Spatially based reconstruction of daily precipitation instrumental data series. Climate Research, 73, 167–186. 10.3354/cr01476

[mec15009-bib-0070] Slafer, G. A. , & Rawson, H. M. (1994). Sensitivity of wheat phasic development to major environmental factors: A re‐examination of some assumptions made by physiologists and modellers. Functional Plant Biology, 21, 393–426. 10.1071/PP9940393

[mec15009-bib-0071] Sreenivasulu, N. , & Schnurbusch, T. (2012). A genetic playground for enhancing grain number in cereals. Trends in Plant Science, 17, 91–101. 10.1016/j.tplants.2011.11.003 22197176

[mec15009-bib-0072] Supit, I. , & Wagner, W. (1999). Analysis of yield, sowing and flowering dates of barley of field survey results in Spain. Agricultural Systems, 59, 107–122. 10.1016/S0308-521X(98)00083-3

[mec15009-bib-0073] Talanova, V. V. , Titov, A. F. , Topchieva, L. V. , & Frolova, S. A. (2012). Effects of abscisic acid treatment on the expression of cysteine proteinase gene and enzyme inhibitor during wheat cold adaptation. Russian Journal of Plant Physiology, 59, 581–585. 10.1134/S1021443712040140

[mec15009-bib-0074] Tomas‐Burguera, M. , Vicente‐Serrano, S. M. , Grimalt, M. , & Beguería, S. (2017). Accuracy of reference evapotranspiration (ET_o_) estimates under data scarcity scenarios in the Iberian Peninsula. Agricultural Water Management, 182, 103–116. 10.1016/j.agwat.2016.12.013

[mec15009-bib-0075] Tondelli, A. , Francia, E. , Barabaschi, D. , Pasquariello, M. , & Pecchioni, N. (2011). Inside the CBF locus in Poaceae. Plant Science, 180, 39–45. 10.1016/j.plantsci.2010.08.012 21421345

[mec15009-bib-0076] Vicente‐Serrano, S. M. , Tomas‐Burguera, M. , Beguería, S. , Reig, F. , Latorre, B. , Peña‐Gallardo, M. , … González‐Hidalgo, J. C. (2017). A high resolution dataset of drought indices for Spain. Data, 2, 22 10.3390/data2030022

[mec15009-bib-0077] von Zitzewitz, J. , Szűcs, P. , Dubcovsky, J. , Yan, L. , Francia, E. , Pecchioni, N. , … Skinner, J. S. (2005). Molecular and structural characterization of barley vernalization genes. Plant Molecular Biology, 59, 449–467. 10.1007/s11103-005-0351-2 16235110

[mec15009-bib-0078] Wang, X. , Dinler, B. S. , Vignjevic, M. , Jacobsen, S. , & Wollenweber, B. (2015). Physiological and proteome studies of responses to heat stress during grain filling in contrasting wheat cultivars. Plant Science, 230, 33–50. 10.1016/j.plantsci.2014.10.009 25480006

[mec15009-bib-0079] Xu, Y. , Wu, Y. , Gonda, M. G. , & Wu, J. (2015). A linkage based imputation method for missing SNP markers in association mapping. Journal of Applied Bioinformatics & Computational Biology, 4, 1 10.4172/2329-9533.1000115

[mec15009-bib-0080] Yahiaoui, S. , Cuesta‐Marcos, A. , Gracia, M. P. , Medina, B. , Lasa, J. M. , Casas, A. M. , … Igartua, E. (2014). Spanish barley landraces outperform modern cultivars at low‐productivity sites. Plant Breeding, 133, 218–226. 10.1111/pbr.12148

[mec15009-bib-0081] Yahiaoui, S. , Igartua, E. , Moralejo, M. , Ramsay, L. , Molina‐Cano, J. l. , Ciudad, F. J. , … Casas, A. M. (2008). Patterns of genetic and eco‐geographical diversity in Spanish barleys. Theoretical and Applied Genetics, 116, 271–282. 10.1007/s00122-007-0665-3 18026712

[mec15009-bib-0082] Zapata, L. , Peña‐Chocarro, L. , Pérez‐Jordá, G. , & Stika, H. P. (2004). Early neolithic agriculture in the Iberian Peninsula. Journal of World Prehistory, 18, 283–325. 10.1007/s10963-004-5621-4

[mec15009-bib-0083] Zeven, A. C. (1998). Landraces: A review of definitions and classifications. Euphytica, 104, 127–139. 10.1023/A:1018683119237

[mec15009-bib-0084] Zhang, X. , & Yang, F. (2004). RClimDex user manual. Retrieved from http://etccdi.pacificclimate.org/software.shtml

[mec15009-bib-0085] Zhong, L. , Yang, Q. , Yan, X. , Yu, C. , Su, L. , Zhang, X. , & Zhu, Y. (2017). Signatures of soft sweeps across the Dt1 locus underlying determinate growth habit in soya bean [*Glycine max* (L.) Merr.]. Molecular Ecology, 26, 4686–4699. 10.1111/mec.14209 28627128

