# Supplementary material for: Genetic association with high‐resolution climate data reveals selection footprints in the genomes of barley landraces across the Iberian Peninsula
Source: Mol Ecol. 2019 Apr 2;28(8):1994–2012. doi: 10.1111/mec.15009 (PMC6563438; doi:10.1111/mec.15009)
Supplement: Supplementary file 1 [file MEC-28-1994-s001.pdf]

Title: Genetic association with high-resolution climate data reveals selection footprints in the genomes of barley landraces across the Iberian Peninsula

Authors: Bruno Contreras-Moreira, Roberto Serrano-Notivoli, Naheif E. Mohamed, Carlos P. Cantalapiedra, Santiago Beguería, Ana M. Casas, Ernesto Igartua

Content:

- Figure S1. Map of observatories of temperature and precipitation that provided daily data from 1981 to 2010.
- Figure S2. Example of the gridded daily dataset of precipitation and temperature…
- Figure S3. Examples of random Gaussian fields used in the analysis as dummy variables…
- Figure S4. Correlation plot of agroclimatic variables...
- Figure S5. Dendrogram corresponding to the hierarchical cluster analysis of agroclimatic variables, with indication of 10 clusters chosen for variable selection.
- Figure S6. Examples of maps of the agroclimatic variables...
- Figure S7. Variables' loadings in the first component of the principal component analysis of the agroclimatic variables.
- Figure S8. Variables' loadings in the second component of the principal component analysis of the agroclimatic variables.
- Figure S9. Variables' loadings in the third component of the principal component analysis of the agroclimatic variables.
- Figure S10. Maps of the first three PCA components of the agroclimatic variables…
- Figure S11. Dendrogram of landraces derived from a median covariance matrix computed by Bayenv2 from 711 SNP markers…
- Figure S12. Distribution of median Bayes Factors (BF, top) and Spearman correlation coefficients (bottom)...
- Figure S13. Structure analysis of 135 barley landraces…
- Figure S14. Plot of the two first coordinates of a factorial analysis run with 8457 markers and 135 barley landraces, with software DARwin 6.0.4…
- Figure S15. Distribution of XtX estimates produced by 3 replicates of Bayenv2...
- Figure S16. Plot of XtX estimates produced by Bayenv2 and BayPass.
- Figure S17. Plots of the seven barley chromosomes, displaying LD, heterozygosity, XtX…

- Figure S18. Graphical genotypes for the regions of chromosomes 3H and 5H, identified as two of the main possible selection footprint between germplasm groups…
- Figure S19. Proportion of total variation (adjusted R2) among accessions for germplasm group distribution explained in RDA by agro-climatic variables or spatial structure…
- Figure S20. Manhattan plots showing median Bayes Factors (BF) estimated…in 135 barley landraces and 20 agroclimatic variables. A null (identity) covariance matrix was used during these simulations…
- Figure S21. Manhattan plots showing median Bayes Factors (BF) estimated...in 135 barley landraces and 20 agroclimatic variables. Population structure was captured in a covariance matrix.

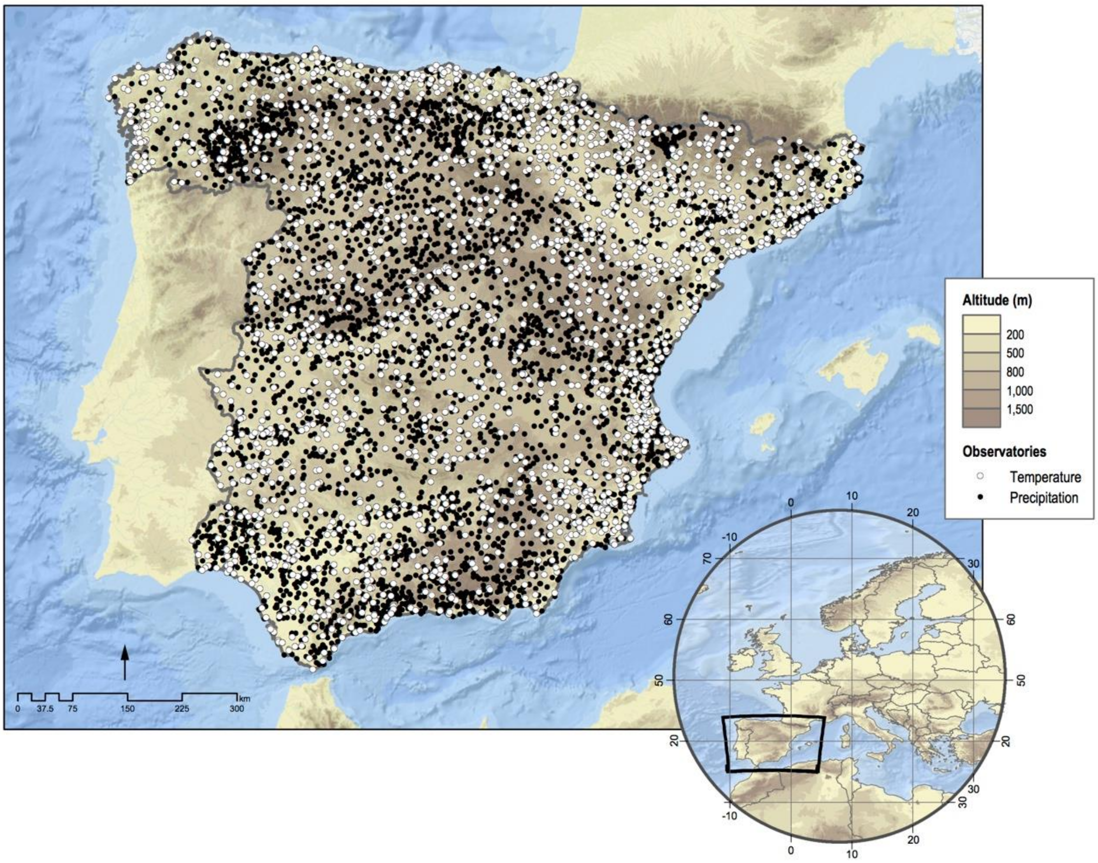

Figure S1. Map of observatories of temperature (n=2,087) and precipitation (n=6,952) which provided daily data from 1981 to 2010.

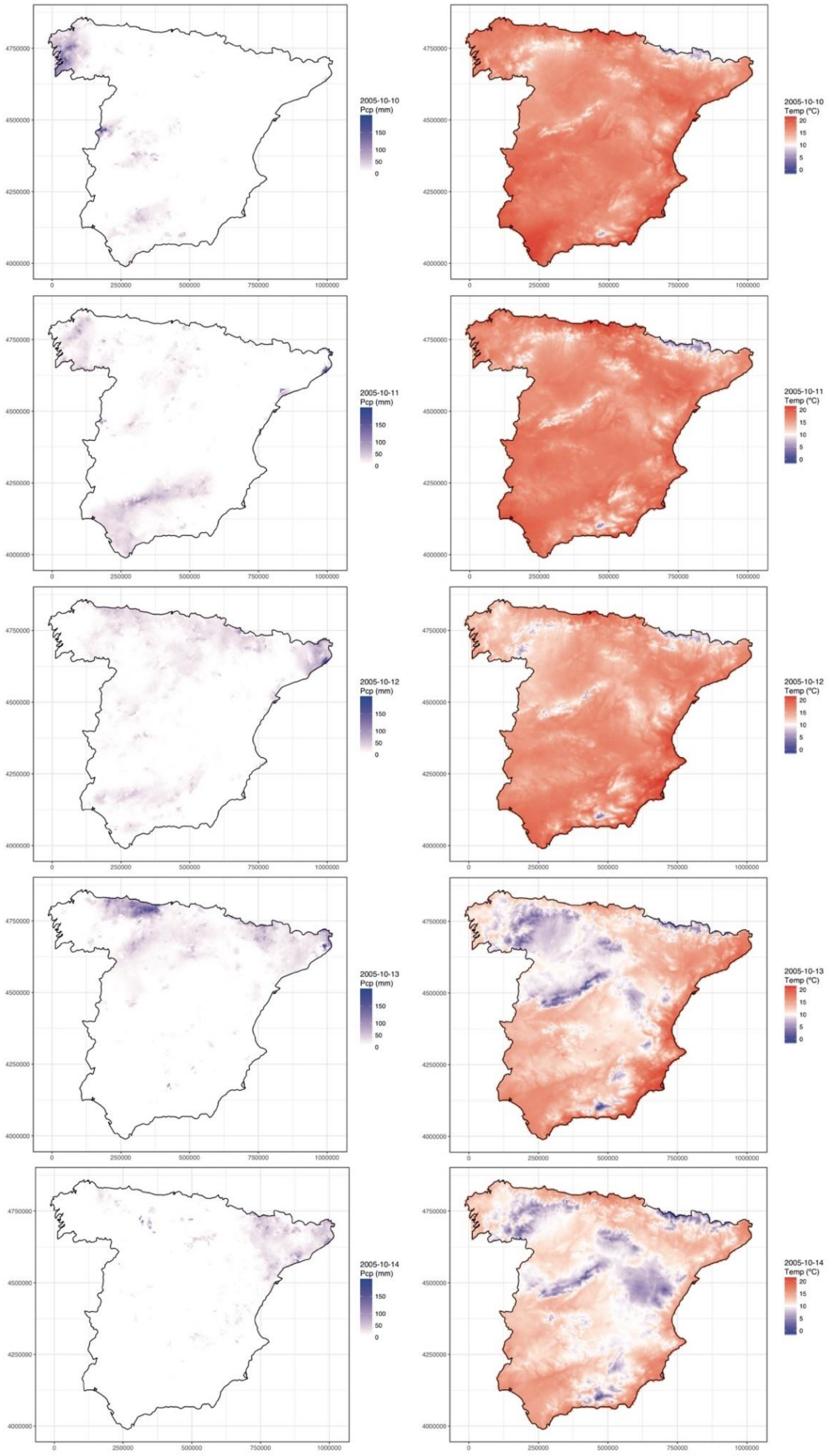

Figure S2. Example of the gridded daily dataset of precipitation and temperature, represented in a UTM-30N projection, axis in m.

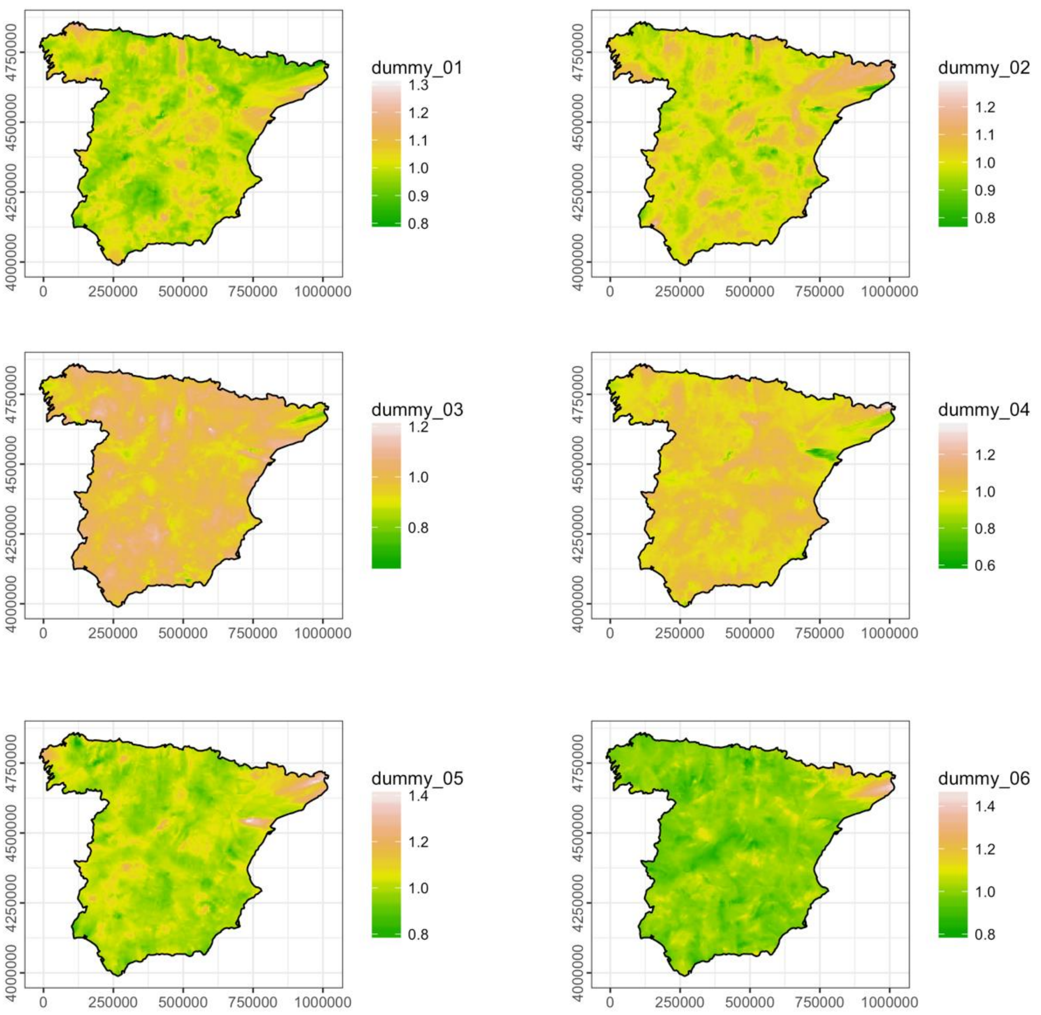

Figure S3. Examples of random Gaussian fields used in the analysis as dummy variables, represented in a UTM-30N projection, axis in m. The simulated fields were generated using the `gstat` R package (Pebesma, 2004), with the following parameters: model=Exponential; partial sill= 0.025; range=5, nmax=20.

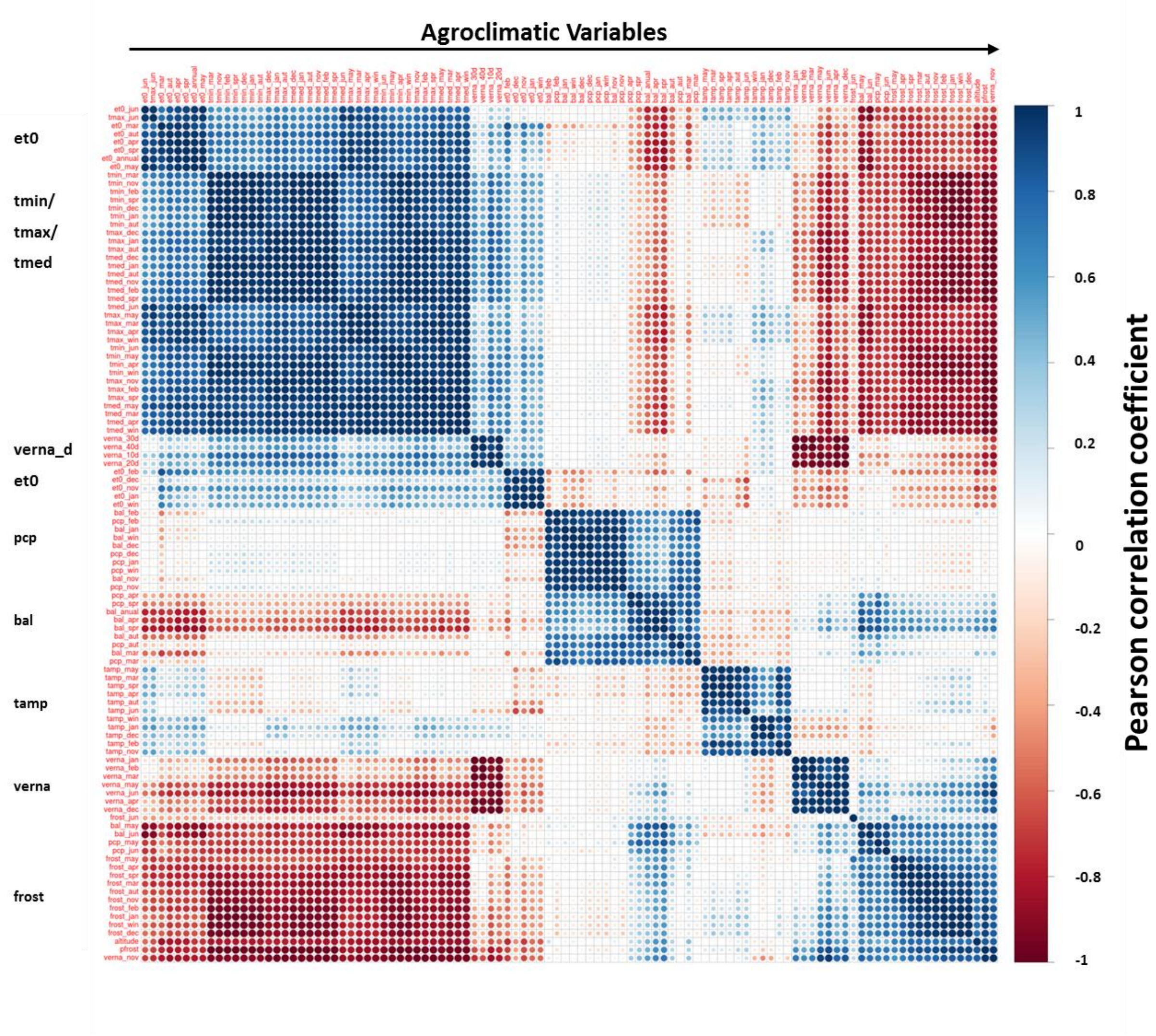

Figure S4. Correlation plot of agroclimatic variables obtained with R package `corrplot` (Wei and Simko, 2016), using hierarchical Ward.D2 clustering for variable sorting.

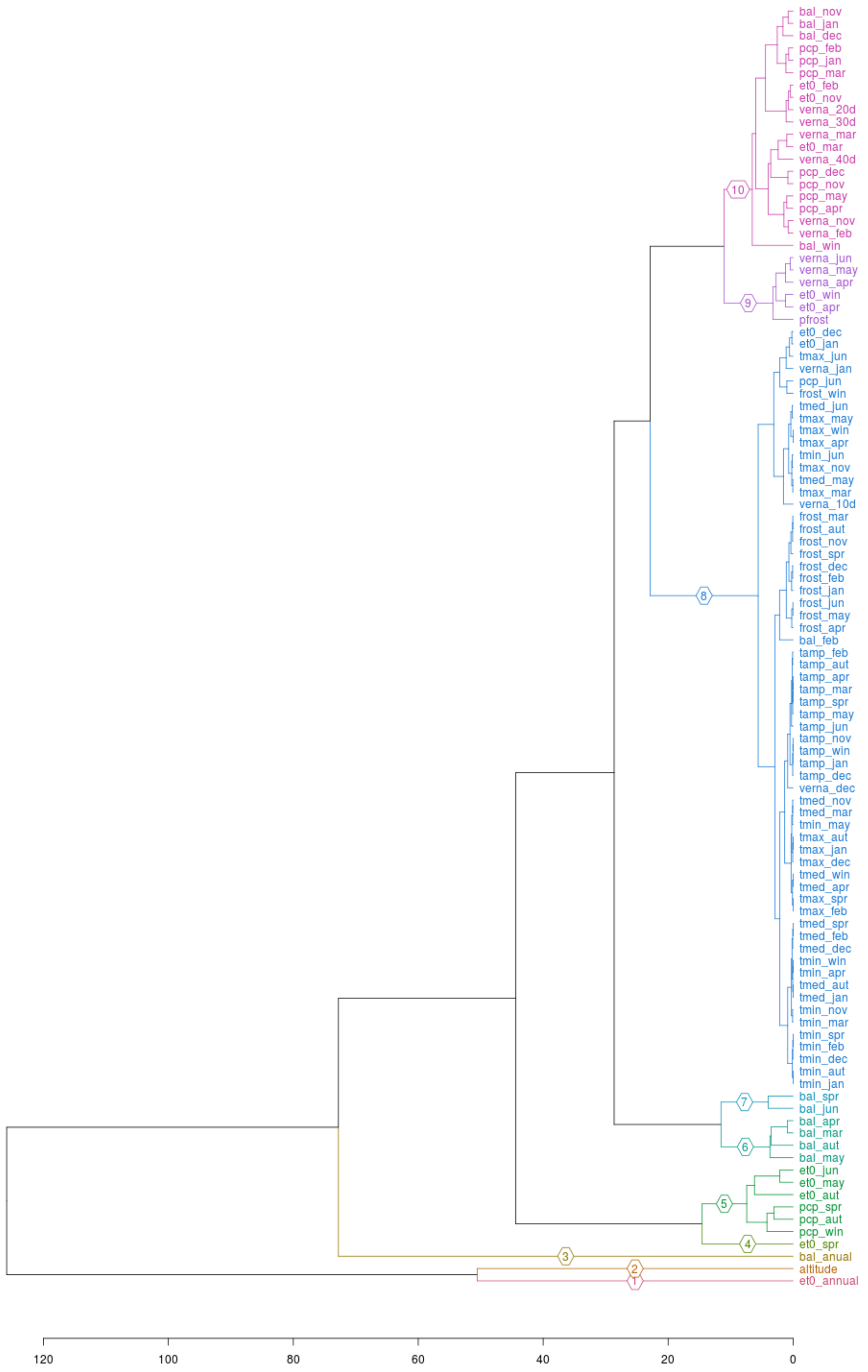

Figure S5. Dendrogram corresponding to the hierarchical cluster analysis of agro-climatic variables, with indication of 10 clusters chosen for variable selection.

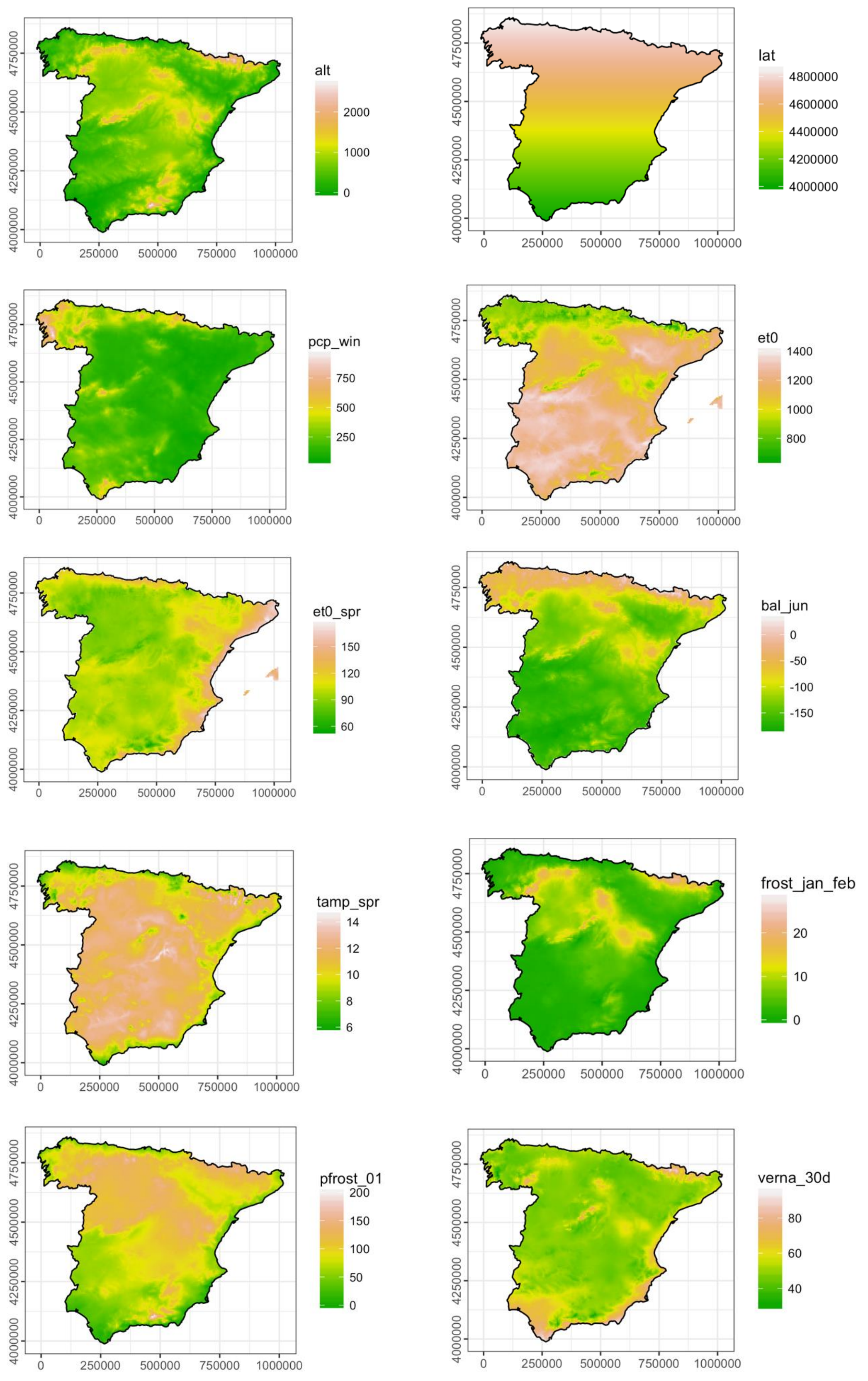

Figure S6. Examples of maps of the agroclimatic variables computed from the daily grids, and used in the analysis, represented in a UTM-30N projection, axis in m.

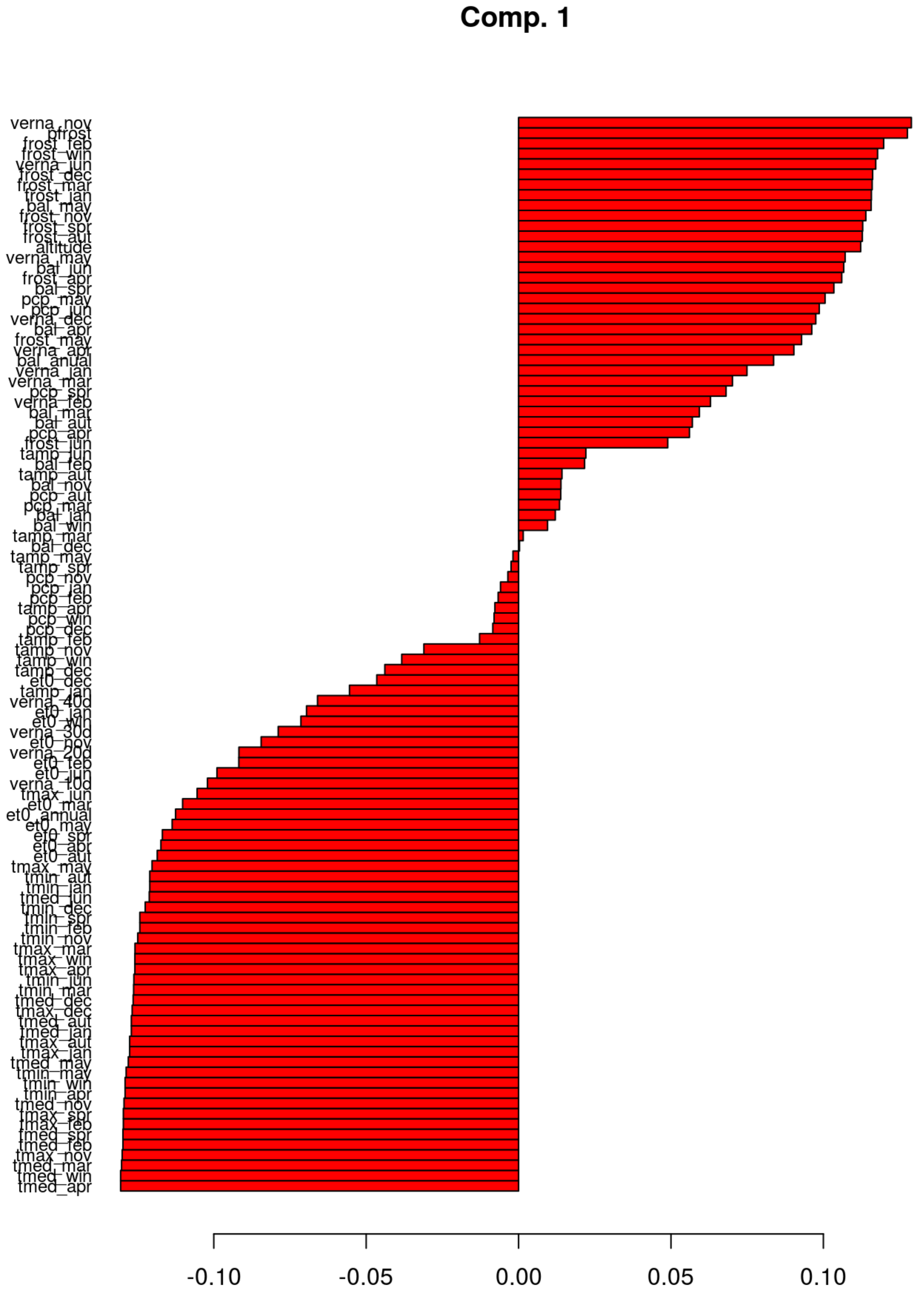

Figure S7. Variables' loadings in the first component of the principal component analysis of the agroclimatic variables.

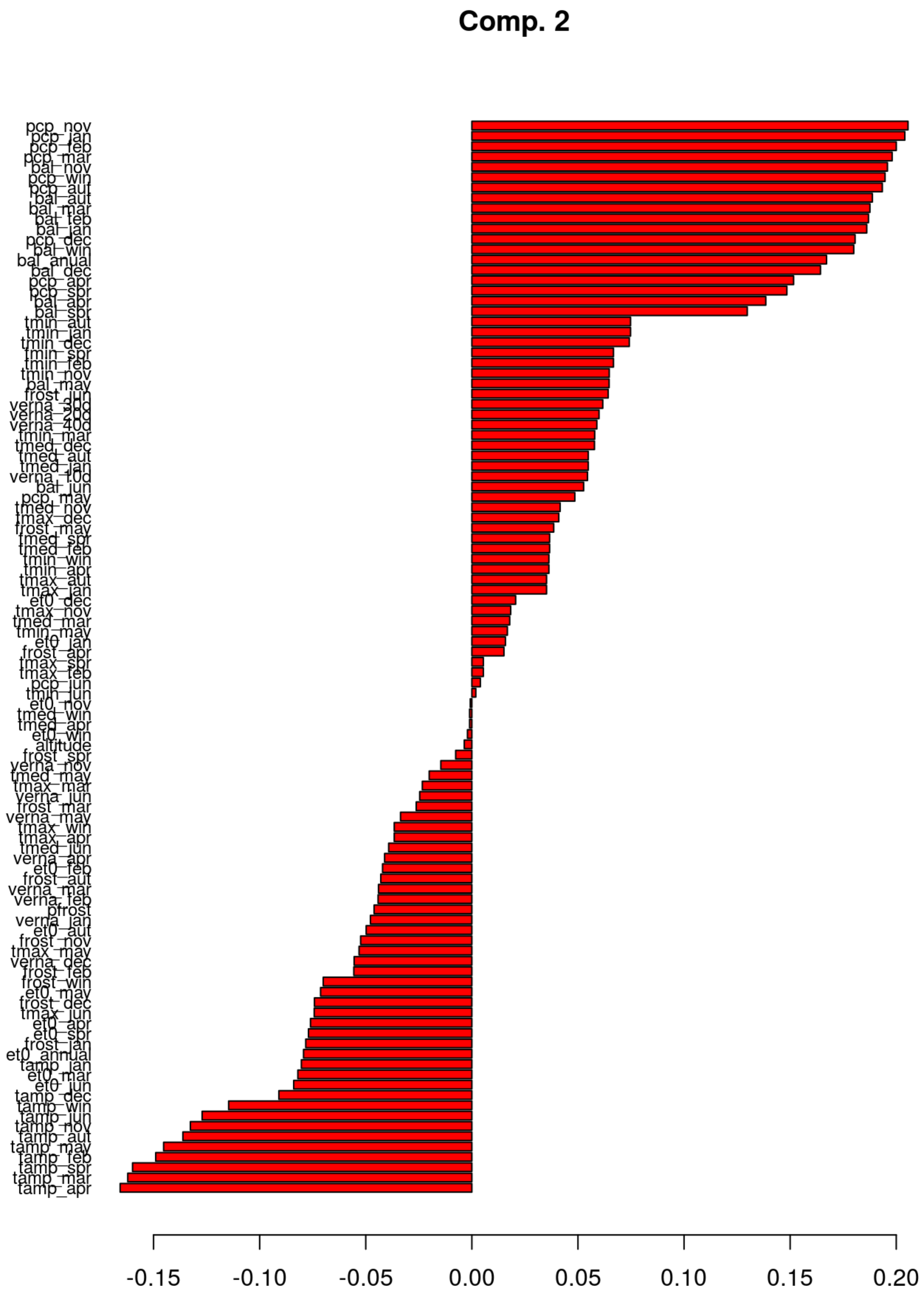

Figure S8. Variables' loadings in the second component of the principal component analysis of the agroclimatic variables.

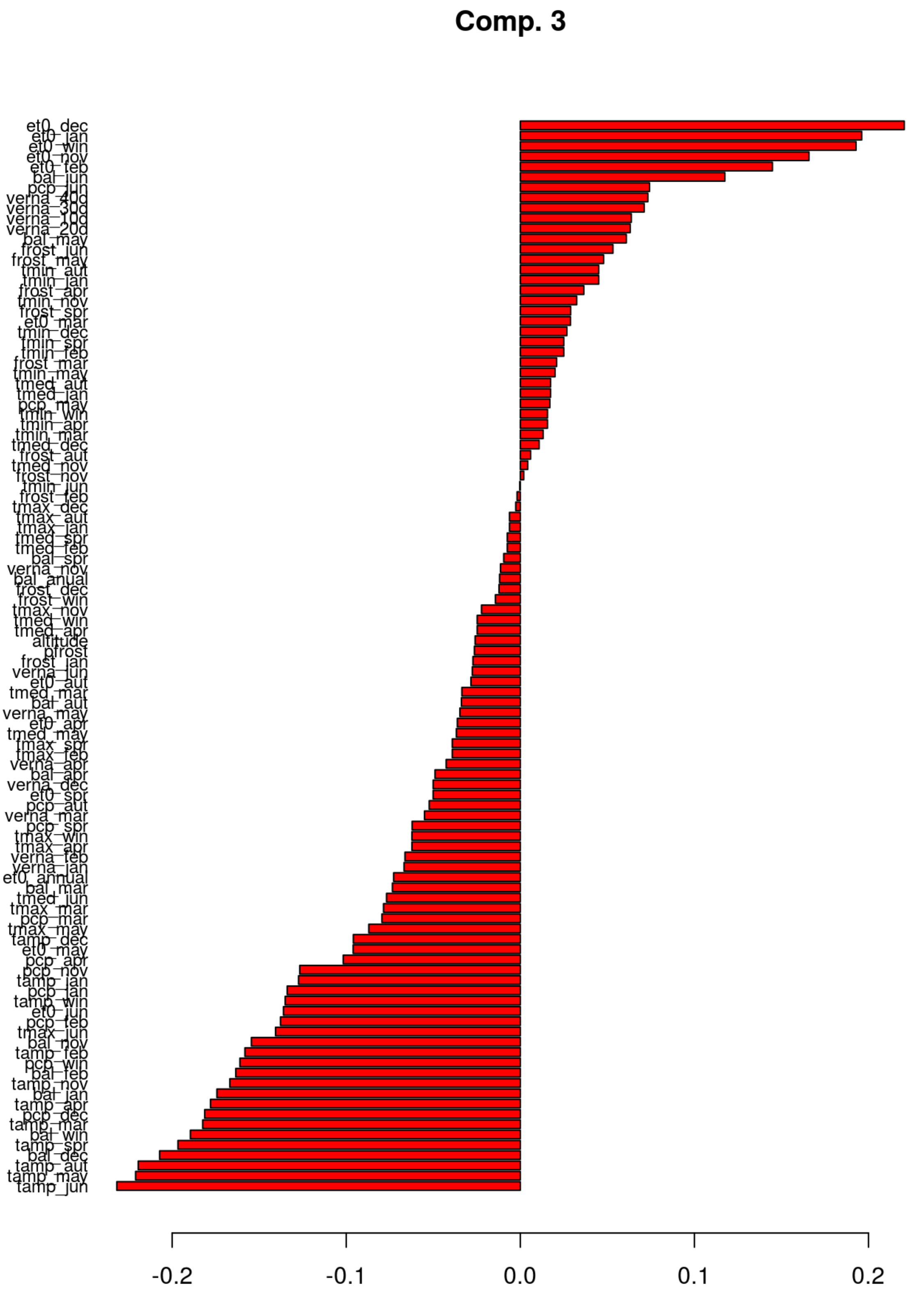

Figure S9. Variables' loadings in the third component of the principal component analysis of the agroclimatic variables.

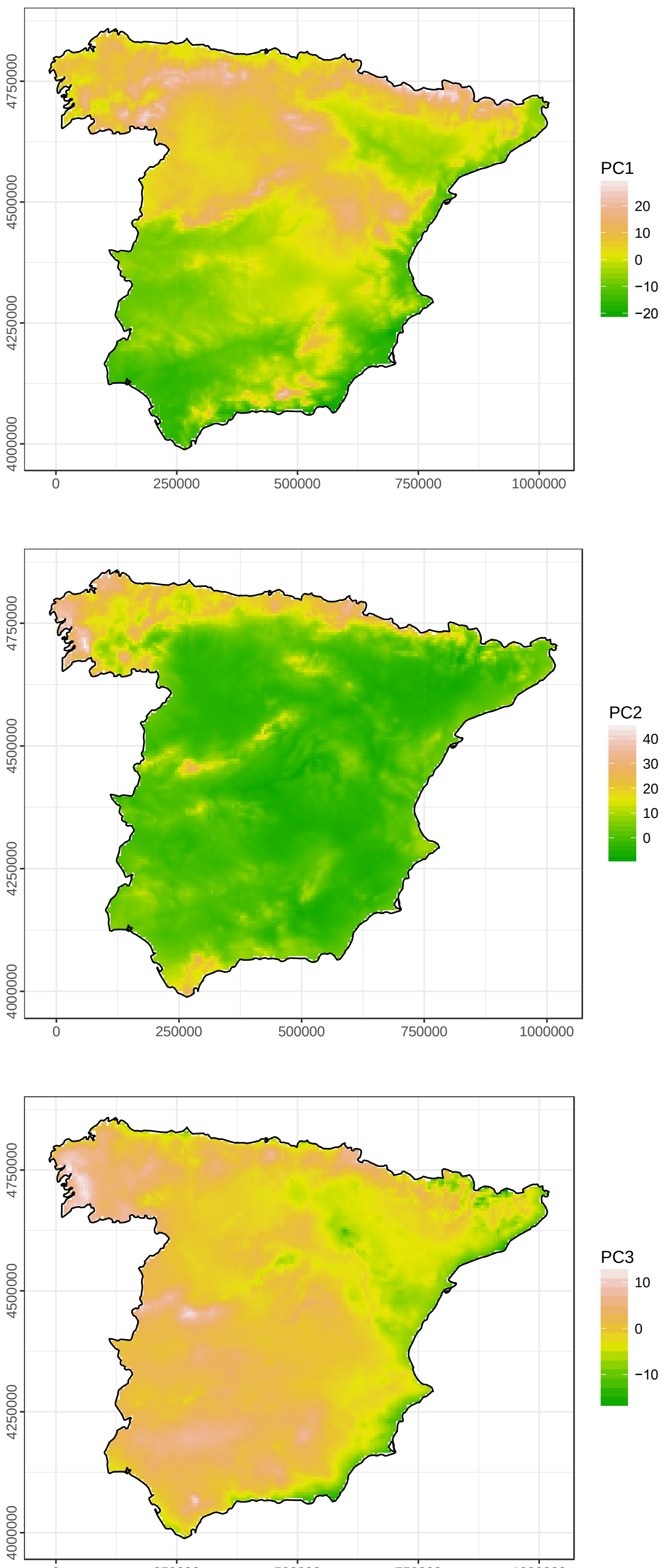

Figure S10. Maps of the first three PCA components of the agroclimatic variables, represented in a UTM-30N projection, axis in m.

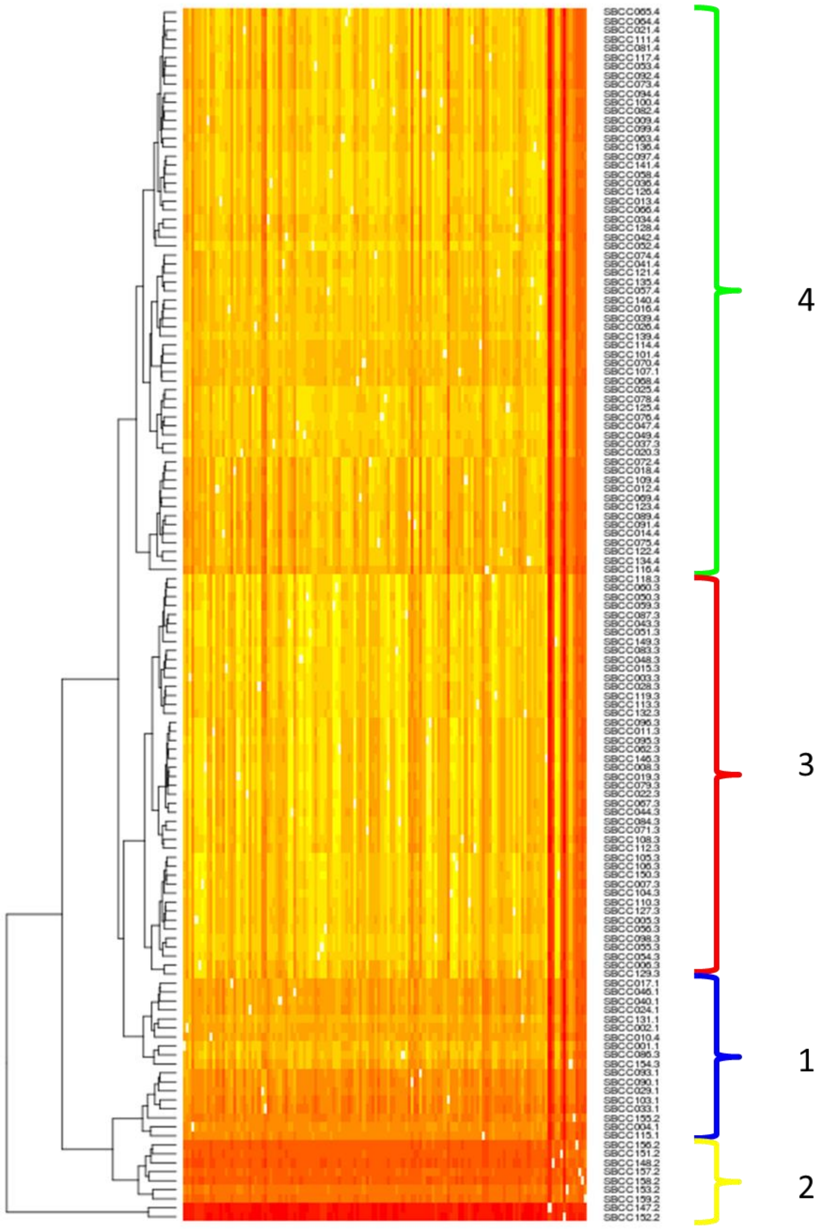

Figure S11. Dendrogram of landraces derived from a median covariance matrix computed by Bayenv2 from 711 SNP markers. Covariances were first converted to correlations with R function `cov2cor` () and then fed to function `heatmap.2` from the `gplots` package (Warnes et al., 2016). Integers concatenated to each accession number indicate the population (K=4) they belong to, according to STRUCTURE analyses (see Supplementary File 1:SNP_landraces). Brackets with the same colours of populations in Figure 2 indicate groups of accessions clustered in terms of covariance.

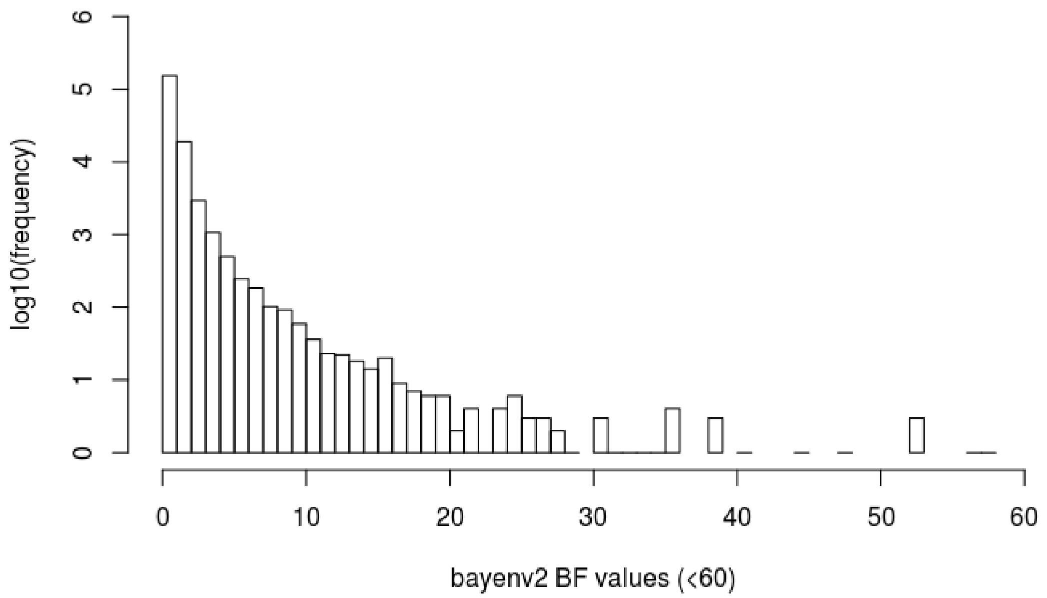

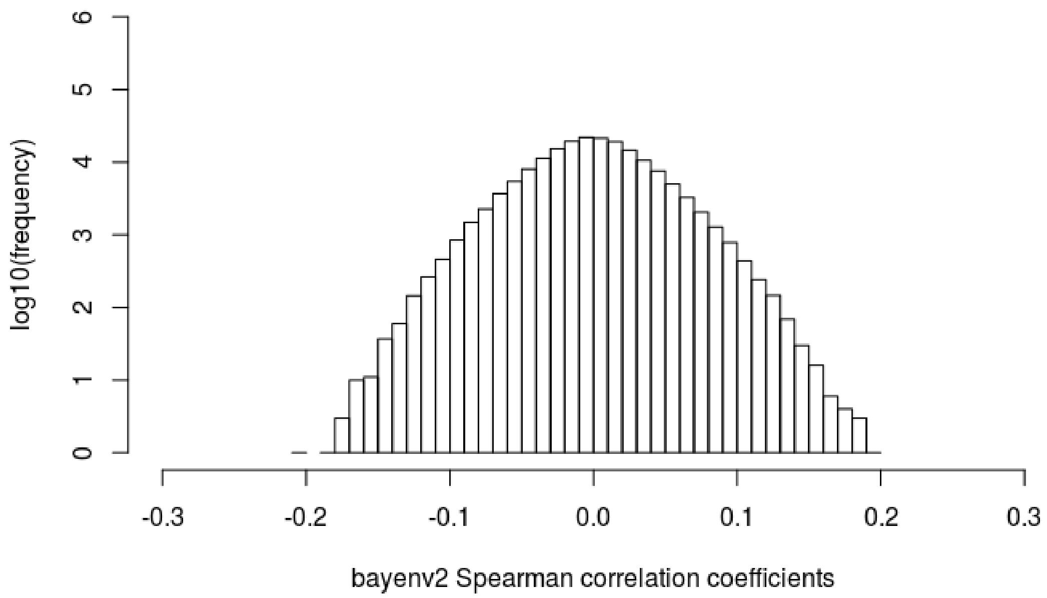

Figure S12. Distribution of median Bayes Factors (BF, top) and Spearman correlation coefficients (bottom) obtained after 5 replicates of association with 8,457 SNPs called in 135 barley landraces and 20 agroclimatic variables.

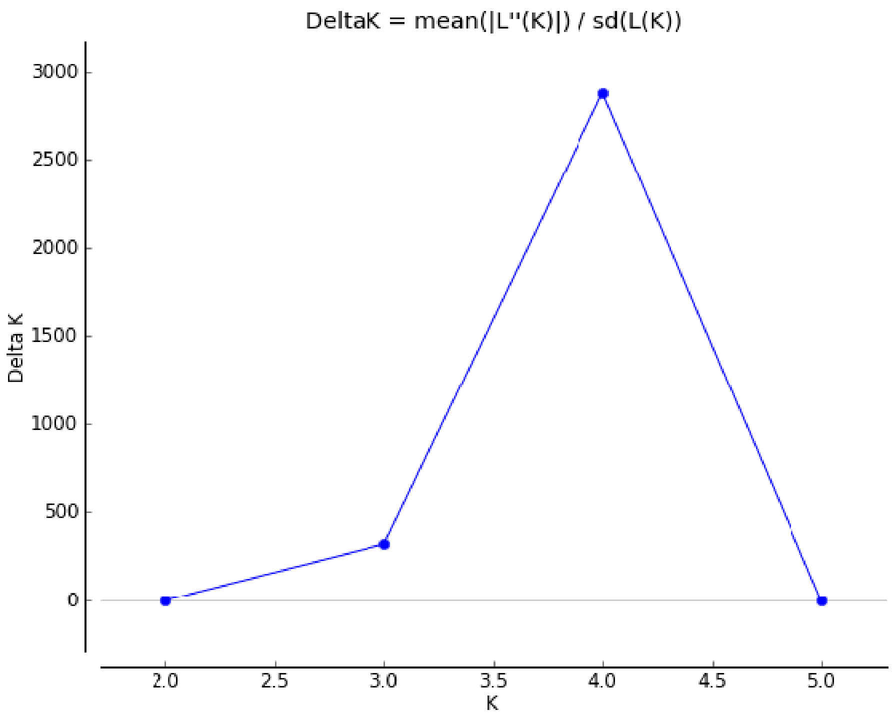

Figure S13. Structure analysis of 135 barley landraces. Graph produced by Structure Harvester software (Earl and vonHoldt, 2012), applying Evanno's ΔK method to estimate the optimal number of subpopulations (K=4).

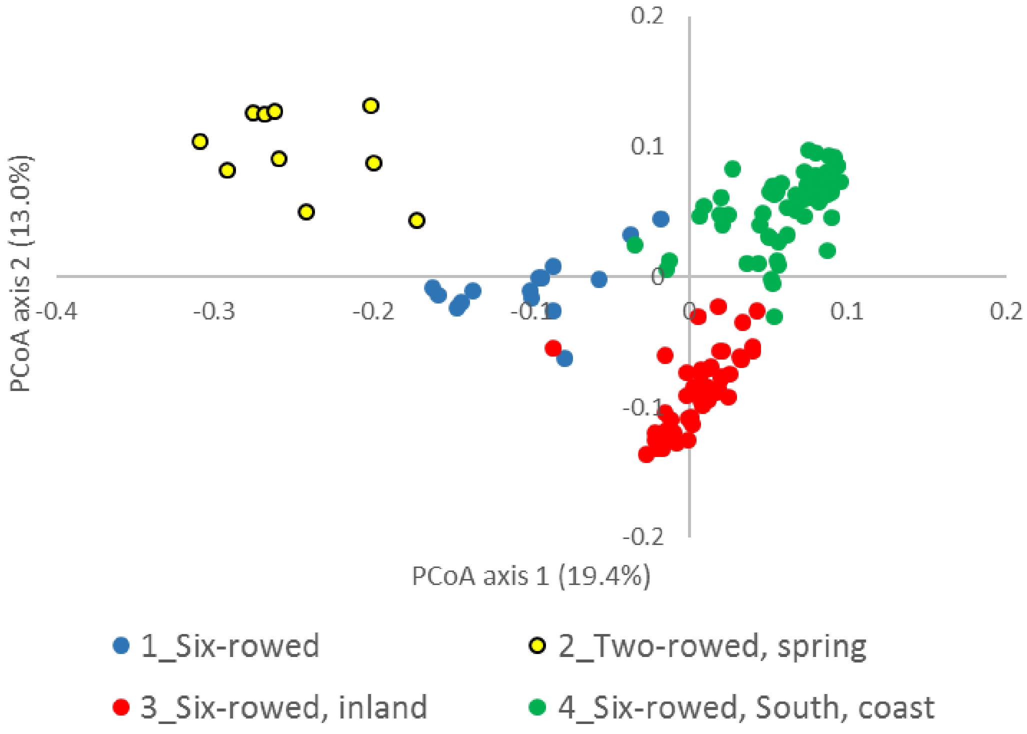

	Longitude	Latitude	Altitude	verna_jan_feb	pfrost
PCoA 1	-0.129	-0.454	-0.307	0.301	-0.451
PCoA 2	0.270	-0.137	-0.293	0.323	-0.340

Figure S14. Top: Plot of the two first coordinates of a factorial analysis run with 8457 markers and 135 barley landraces, with software DARwin 6.0.4 (Perrier and Jacquemoud-Collet, 2006). Genotypes are colour-coded according to the membership to the subpopulations found with Structure (Fig. 3). The first axis differentiates between 2-rowed and 6-rowed accessions and, among these last ones, between groups 3 and 4 vs. group 1. The second axis differentiates group 3 from the rest. Bottom: linear correlation coefficients between genotypic scores at the first and second principal coordinate axes with selected geographic and agro-climatic variables.

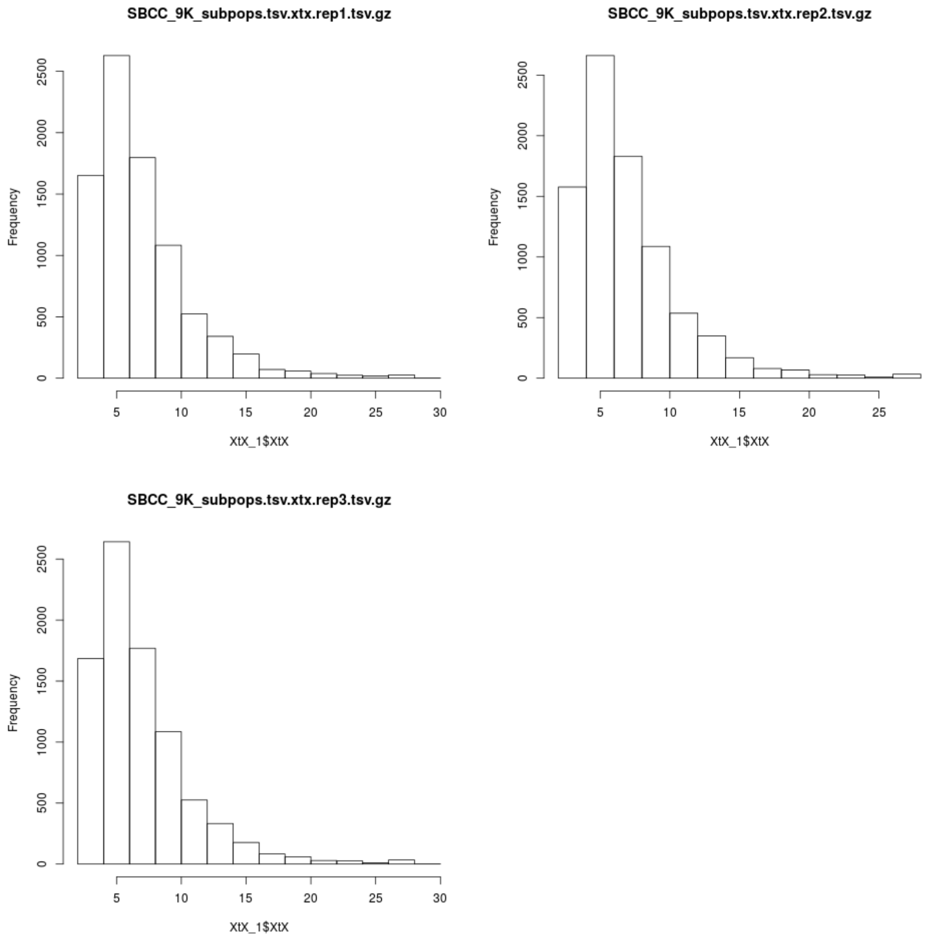

Figure S15. Distribution of XtX estimates produced by 3 replicates of Bayenv2 with 8,457 SNPs called in 135 barley landraces. The expected value for XtX equals the number of populations, k=4 in this work.

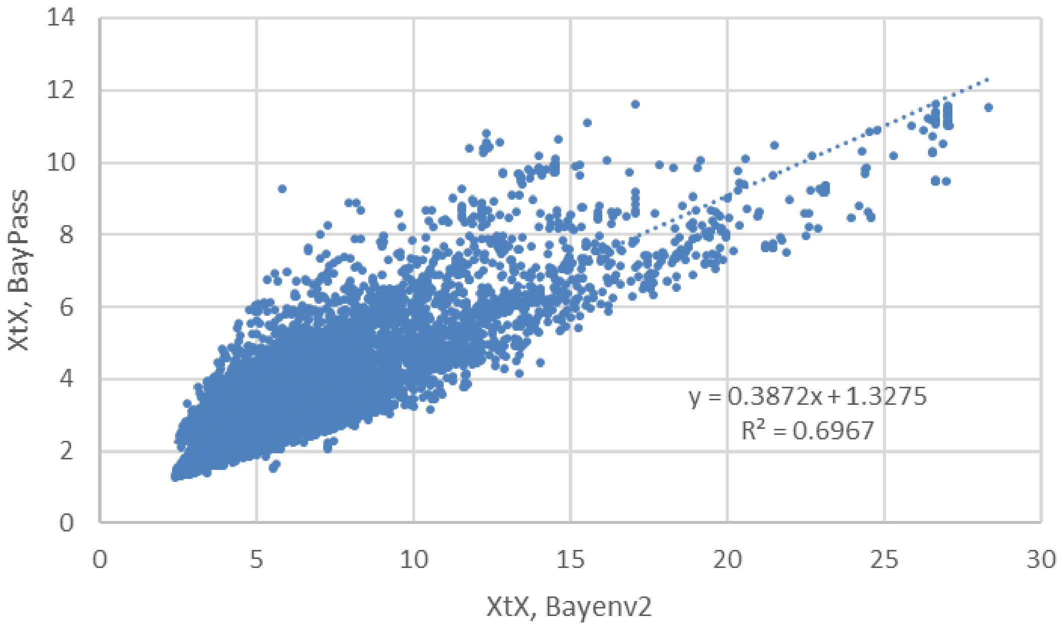

Figure S16. Plot of XtX estimates produced by Bayenv2 and BayPass.

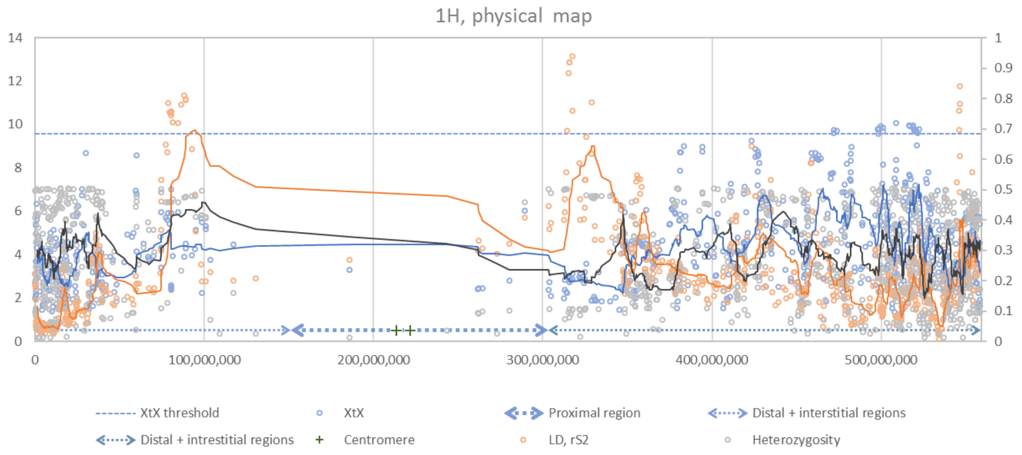
1H, physical map
XtX threshold
XtX
Proximal region
Distal + interstitial regions
Distal + intrestitial regions
Centromere
LD, rS2
Heterozygosity

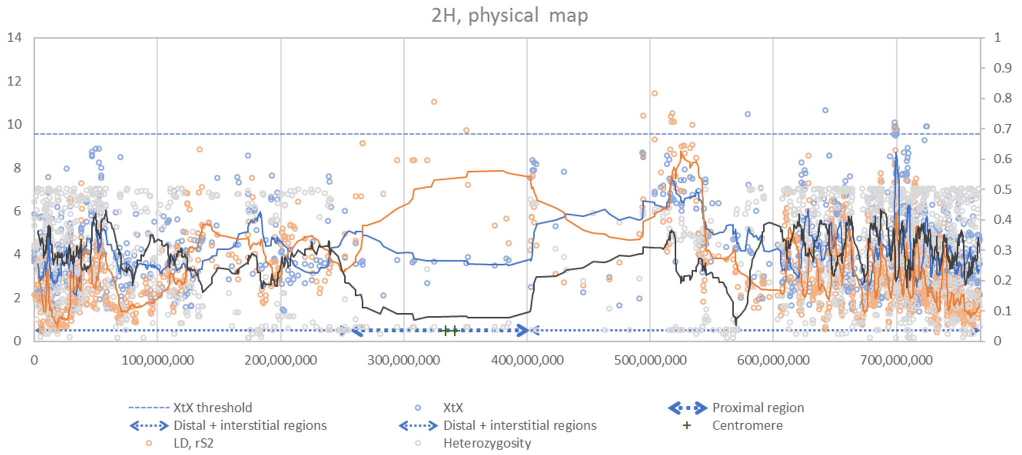
2H, physical map
XtX threshold
XtX
Proximal region
Distal + interstitial regions
Distal + interstitial regions
Centromere
LD, rS2
Heterozygosity

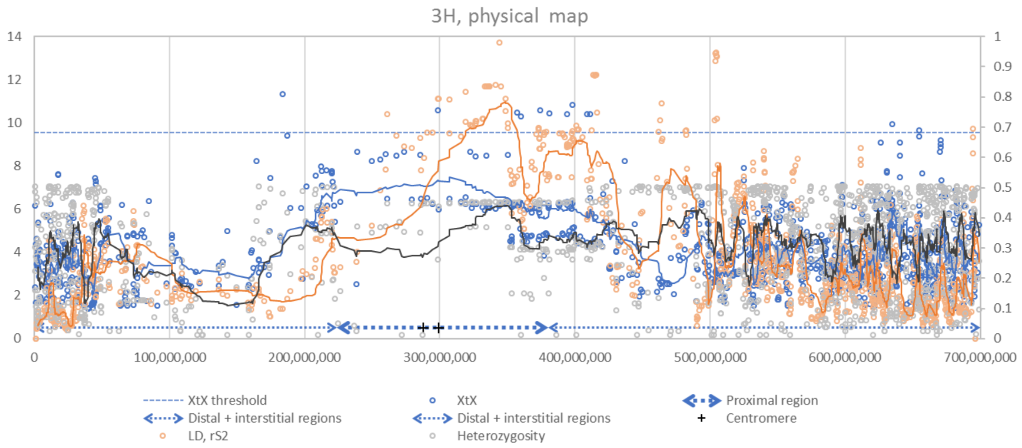
3H, physical map
XtX threshold
XtX
Proximal region
Distal + interstitial regions
Distal + interstitial regions
Centromere
LD, rS2
Heterozygosity

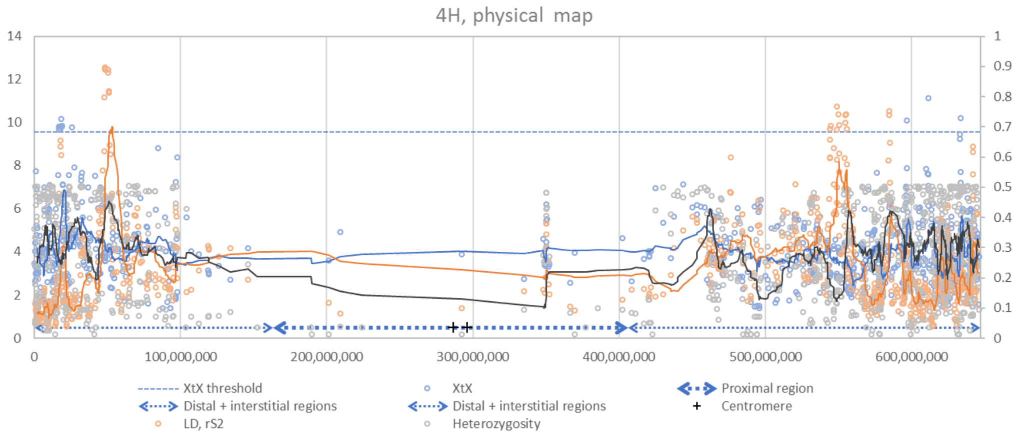
4H, physical map
XtX threshold
XtX
Proximal region
Distal + interstitial regions
Distal + interstitial regions
Centromere
LD, rS2
Heterozygosity

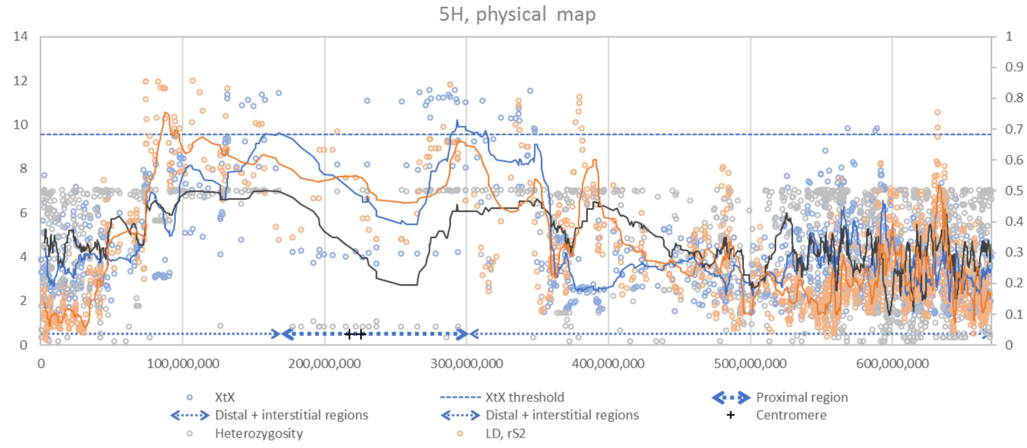

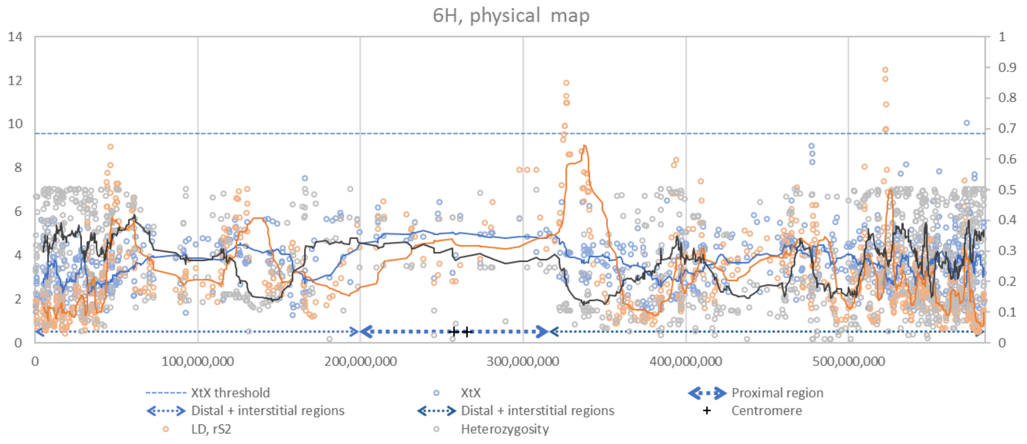

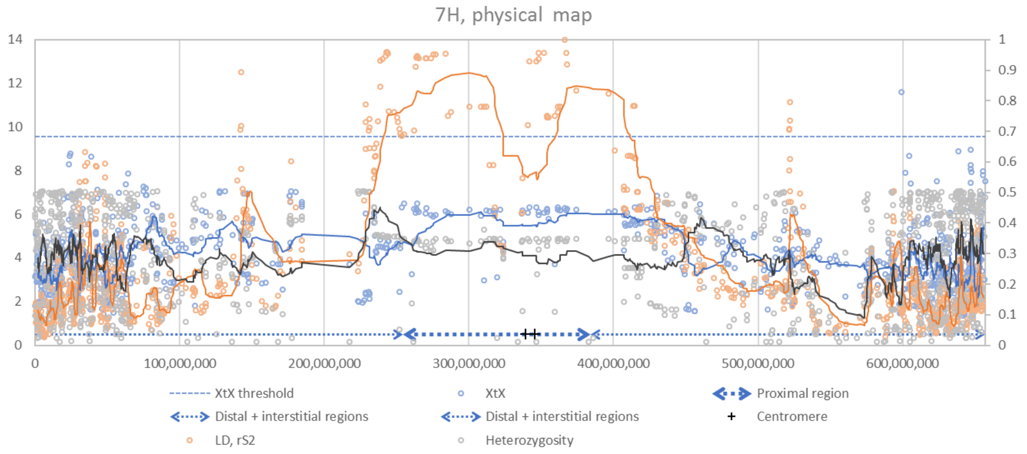

Figure S17. Plots of the seven barley chromosomes, displaying LD (r_s^2 orange circles, referred to the right axis), heterozygosity (grey circles, right axis), population differentiation (XtX, left axis), and 20 SNP moving averages (lines in matching colours). Also displayed, the centromere positions, and the distal, interstitial and proximal zones of each chromosome, as described in Mascher et al. (2017).

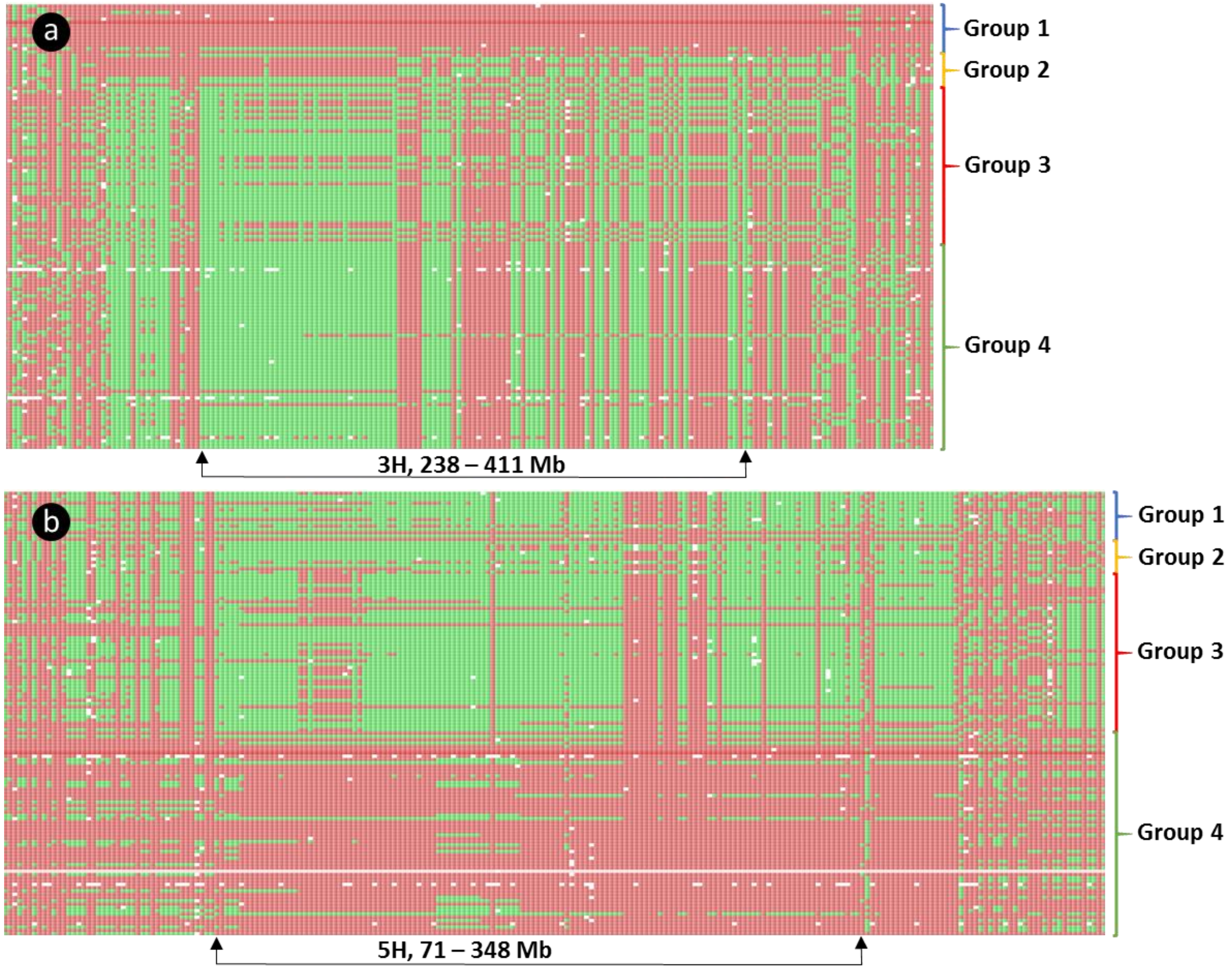

Figure S18. Graphical genotypes (done with Flapjack, Milne et al., 2010) for the regions of a) chromosome 3H and b) chromosome 5H, identified as two of the main possible selection footprints between germplasm groups. Genotypes are in rows, markers in columns. Genotypes ordered according to groups from the Structure analysis (1-4). Alleles are colour coded according to similarity with a chosen genotype, SBCC029 from group 1 in a) and SBCC018 from group 4 in b), to highlight differences among the most relevant groups in each case.

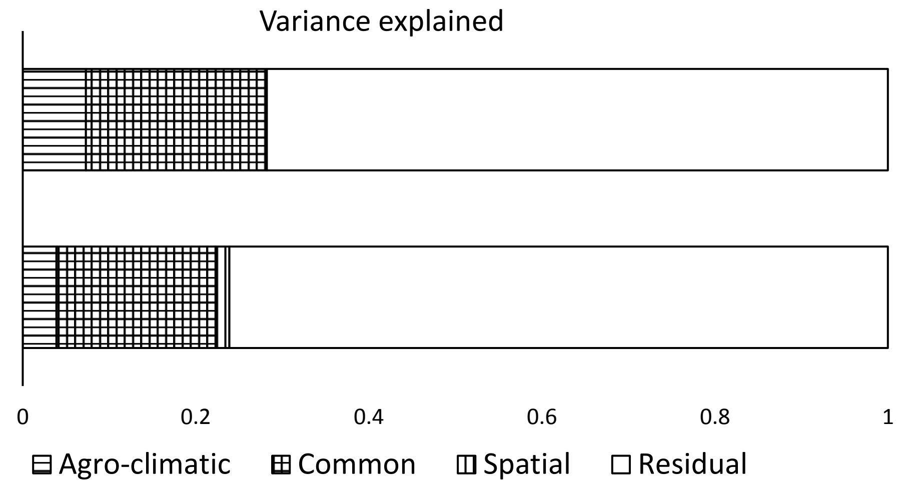

Figure S19. Proportion of total variation (adjusted R^2) among accessions for germplasm group distribution explained in RDA by agro-climatic variables or spatial structure (geographic variables longitude, latitude and altitude). Top, subset of 17 agro-climatic variables; bottom, set of the two most significant agro-climatic variables (pfrost+bal_jun).

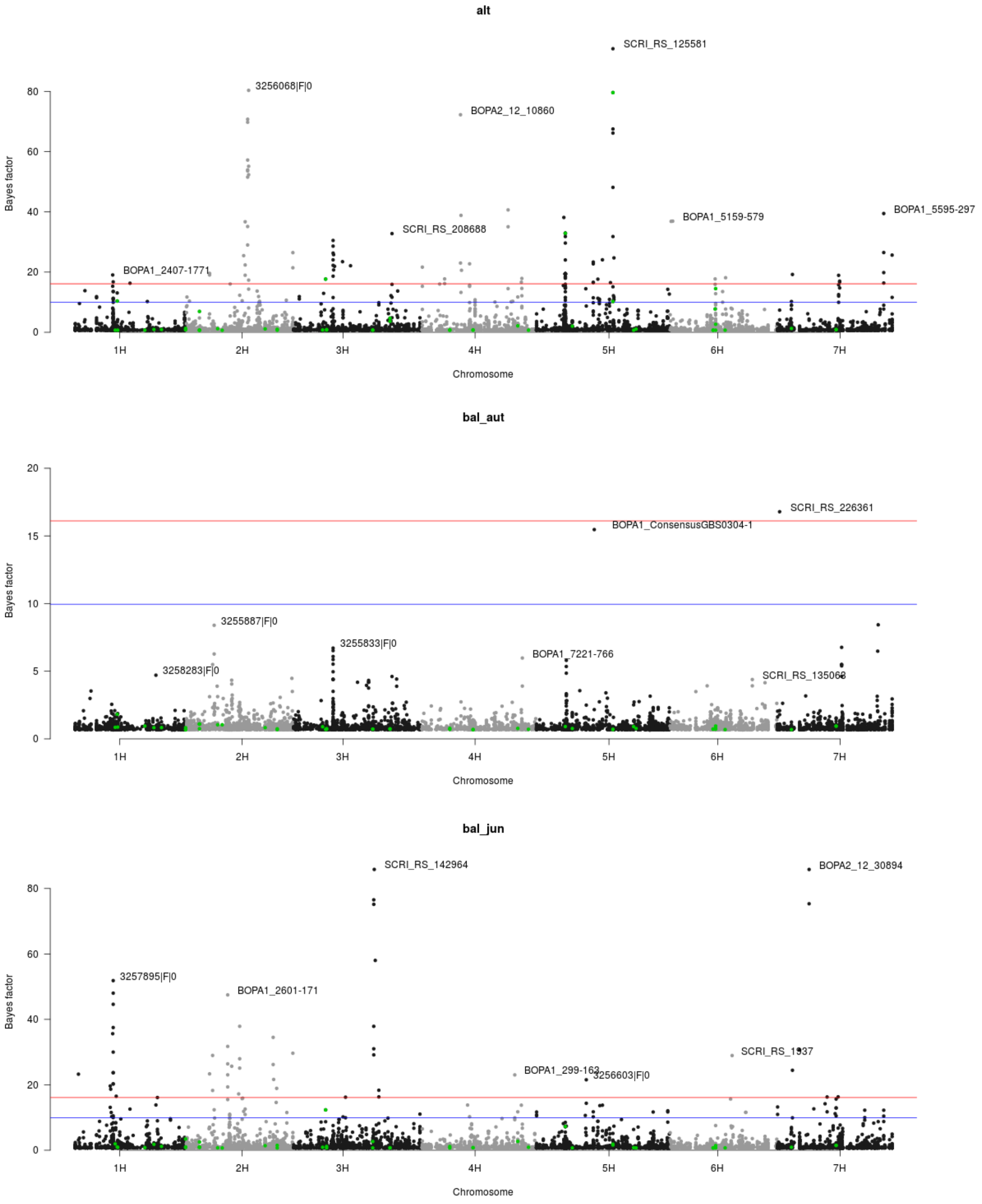
alt
SCRI_RS_125581
3256068|F|0
BOPA2_12_10860
BOPA1_5159-579
BOPA1_5595-297
SCRI_RS_208688
BOPA1_2407-1771
Bayes factor
0
20
40
60
80
1H
2H
3H
4H
5H
6H
7H
Chromosome
bal_aut
SCRI_RS_226361
BOPA1_ConsensusGBS0304-1
3255887|F|0
3255833|F|0
BOPA1_7221-766
3258283|F|0
SCRI_RS_135068
Bayes factor
0
5
10
15
20
1H
2H
3H
4H
5H
6H
7H
Chromosome
bal_jun
SCRI_RS_142964
BOPA2_12_30894
3257895|F|0
BOPA1_2601-171
SCRI_RS_1937
BOPA1_299-163
3256603|F|0
Bayes factor
0
20
40
60
80
1H
2H
3H
4H
5H
6H
7H
Chromosome

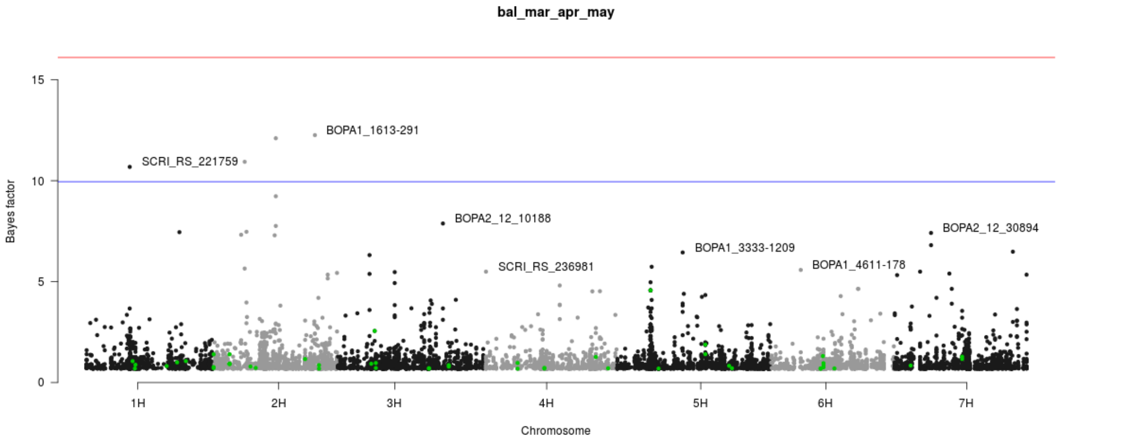
bal_mar_apr_may
Bayes factor
Chromosome
1H
2H
3H
4H
5H
6H
7H
SCRI_RS_221759
BOPA1_1613-291
BOPA2_12_10188
SCRI_RS_236981
BOPA1_3333-1209
BOPA1_4611-178
BOPA2_12_30894

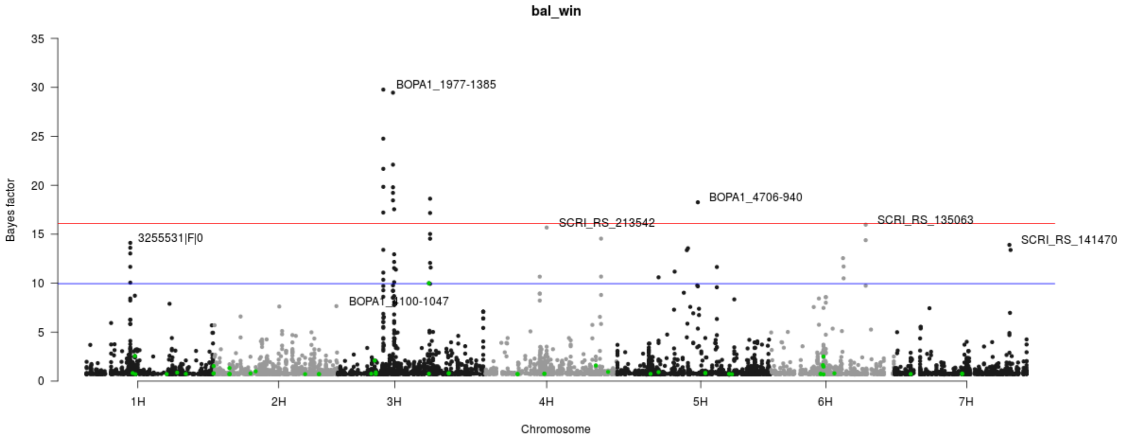
bal_win
Bayes factor
Chromosome
1H
2H
3H
4H
5H
6H
7H
BOPA1_1977-1385
3255531|F|0
SCRI_RS_213542
BOPA1_4706-940
SCRI_RS_135063
SCRI_RS_141470

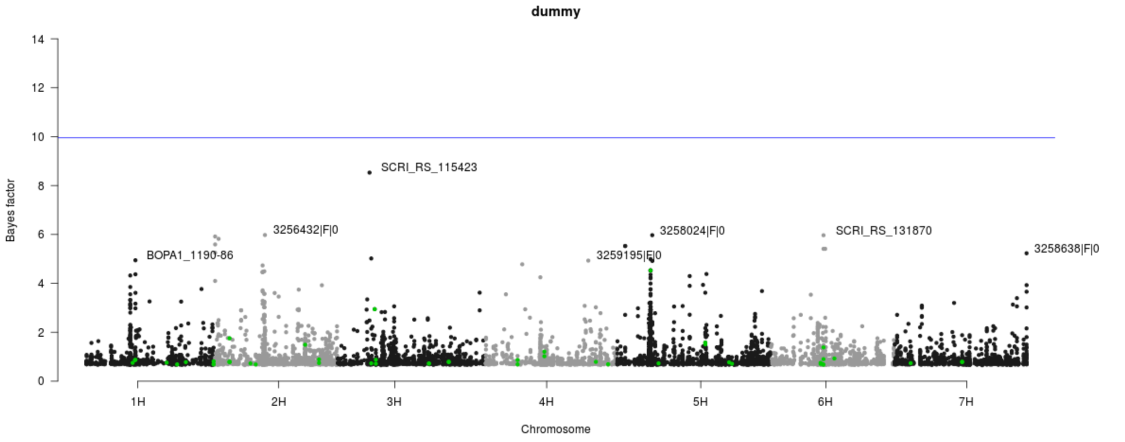
dummy
Bayes factor
Chromosome
1H
2H
3H
4H
5H
6H
7H
SCRI_RS_115423
3256432|F|0
BOPA1_1190-86
3258024|F|0
3259195|F|0
SCRI_RS_131870
3258638|F|0

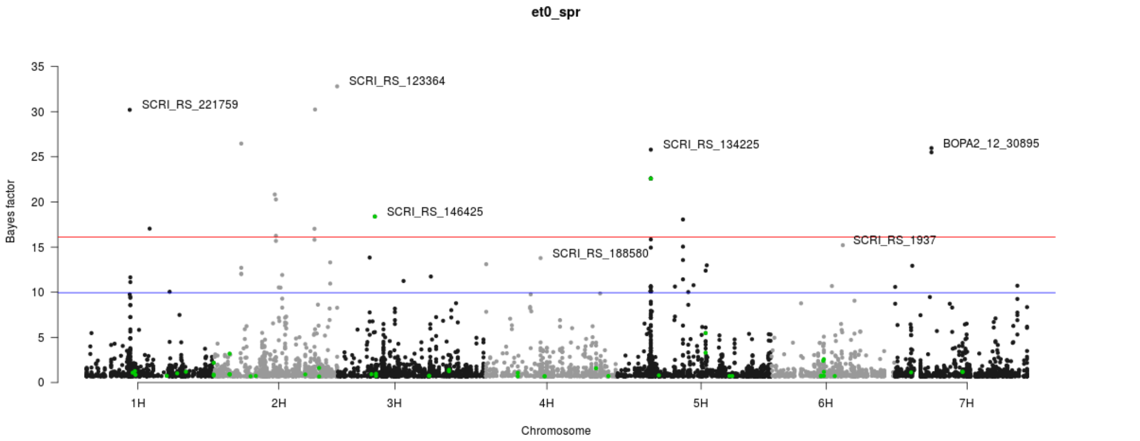

et0_spr
Bayes factor
Chromosome
SCRI_RS_221759
SCRI_RS_123364
SCRI_RS_134225
BOPA2_12_30895
SCRI_RS_146425
SCRI_RS_188580
SCRI_RS_1937

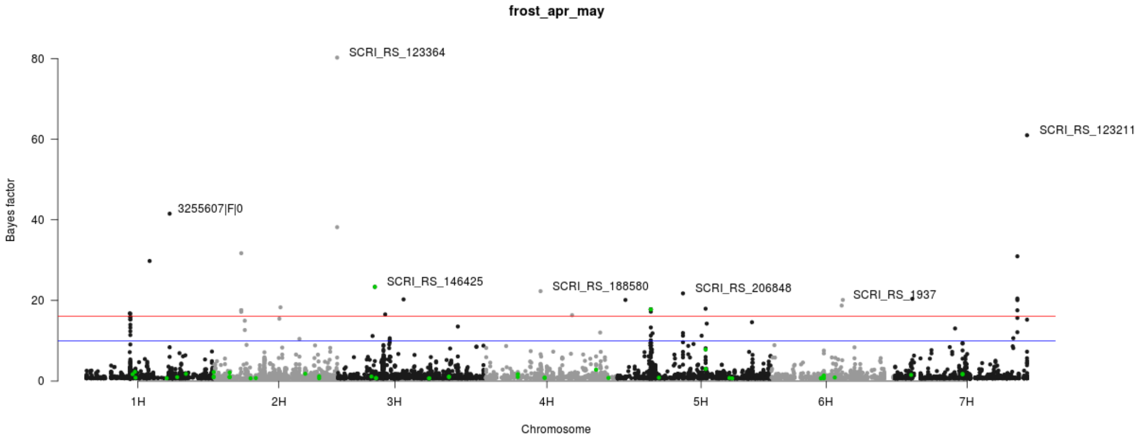

frost_apr_may
Bayes factor
Chromosome
SCRI_RS_123364
SCRI_RS_123211
3255607|F|0
SCRI_RS_146425
SCRI_RS_188580
SCRI_RS_206848
SCRI_RS_1937

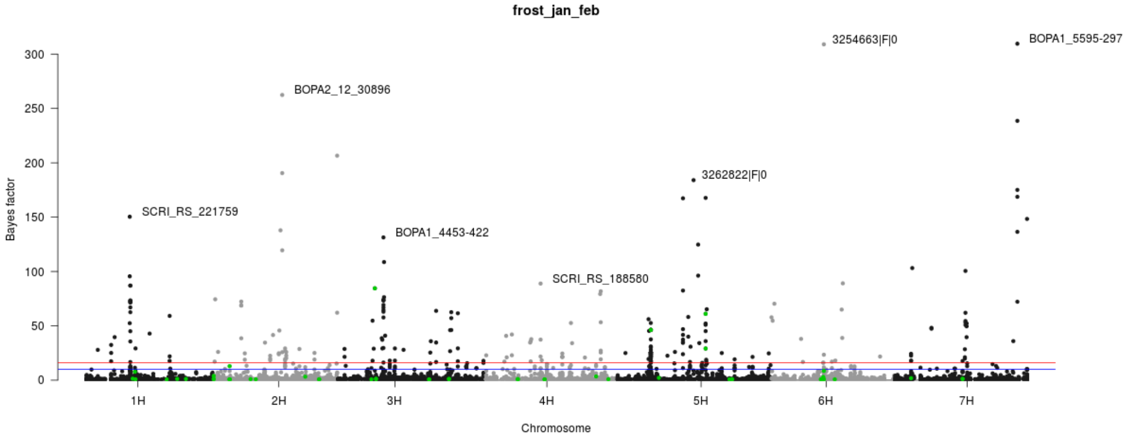

frost_jan_feb
Bayes factor
Chromosome
3254663|F|0
BOPA1_5595-297
BOPA2_12_30896
3262822|F|0
SCRI_RS_221759
BOPA1_4453-422
SCRI_RS_188580

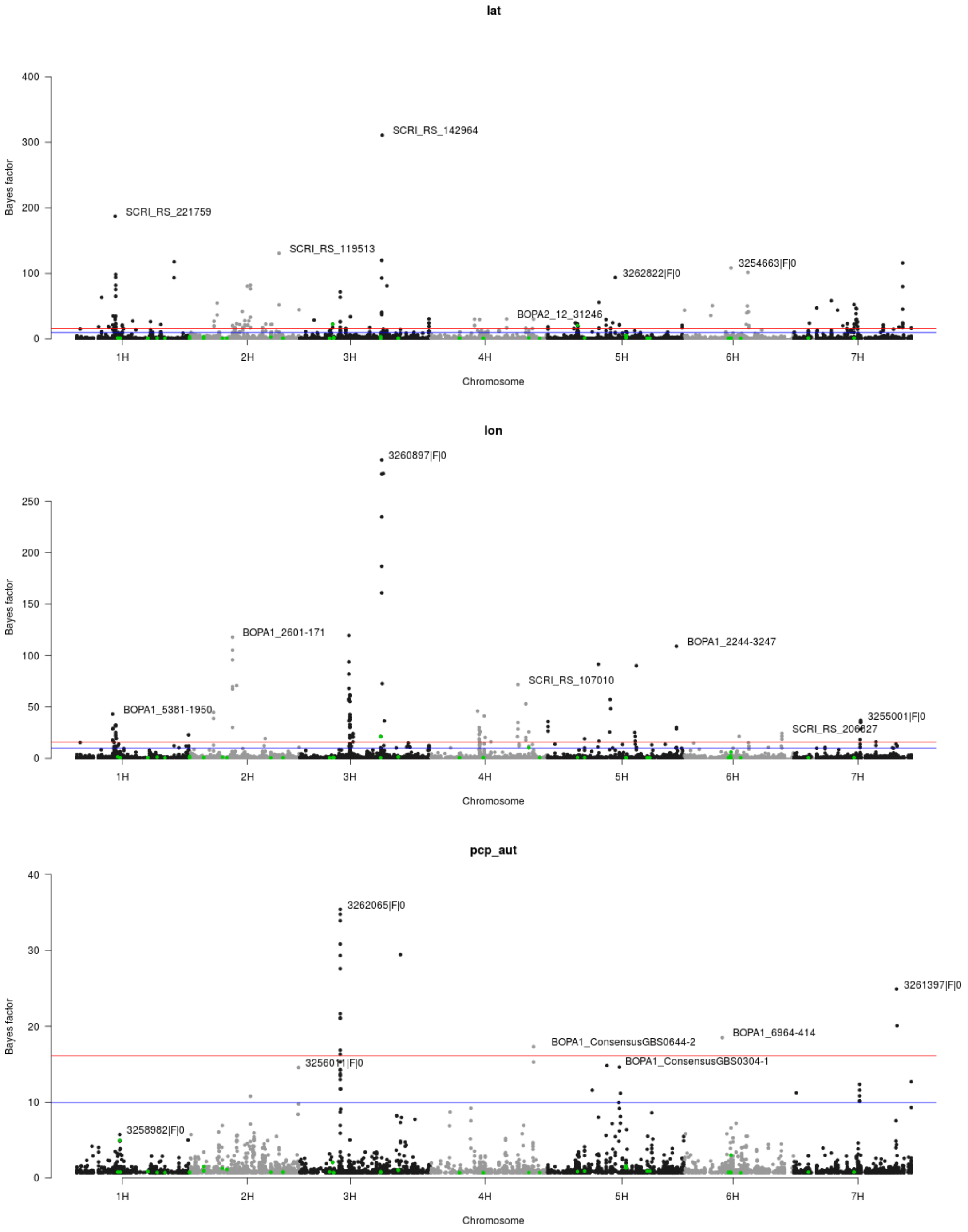
lat
SCRI_RS_142964
SCRI_RS_221759
SCRI_RS_119513
3262822|F|0
3254663|F|0
BOPA2_12_31246
Bayes factor
Chromosome
lon
3260897|F|0
BOPA1_2601-171
BOPA1_2244-3247
SCRI_RS_107010
BOPA1_5381-1950
3255001|F|0
SCRI_RS_206827
Bayes factor
Chromosome
pcp_aut
3262065|F|0
3261397|F|0
BOPA1_ConsensusGBS0644-2
BOPA1_6964-414
BOPA1_ConsensusGBS0304-1
3256011|F|0
3258982|F|0
Bayes factor
Chromosome
1H
2H
3H
4H
5H
6H
7H

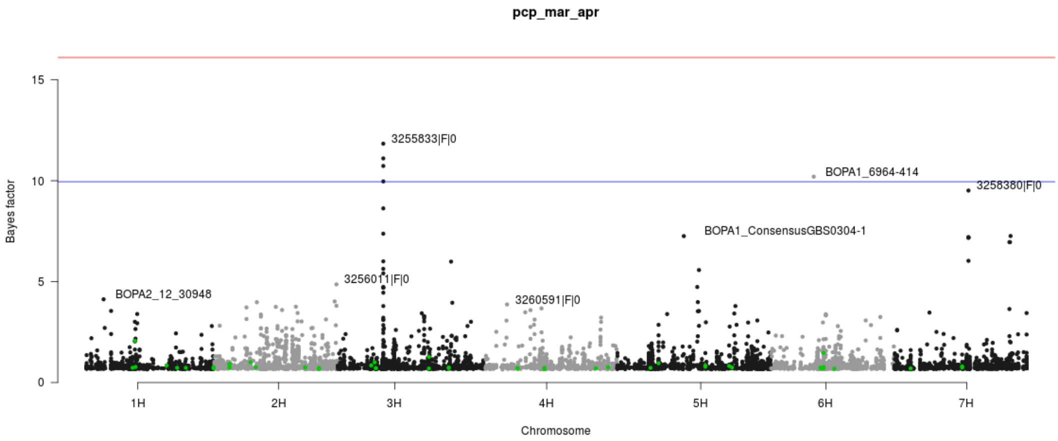
pcp_mar_apr
Bayes factor
Chromosome
1H
2H
3H
4H
5H
6H
7H
3255833|F|0
BOPA1_6964-414
3258380|F|0
BOPA1_ConsensusGBS0304-1
3256011|F|0
3260591|F|0
BOPA2_12_30948

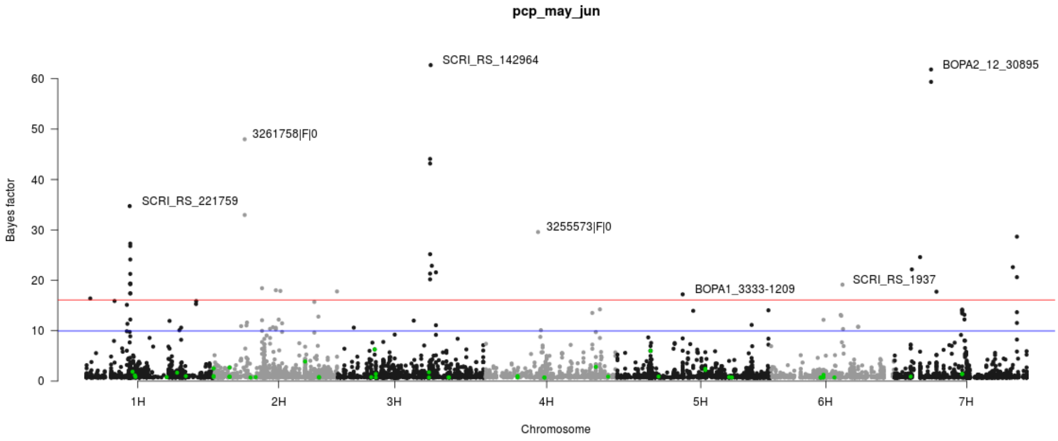
pcp_may_jun
Bayes factor
Chromosome
1H
2H
3H
4H
5H
6H
7H
SCRI_RS_142964
BOPA2_12_30895
3261758|F|0
SCRI_RS_221759
3255573|F|0
BOPA1_3333-1209
SCRI_RS_1937

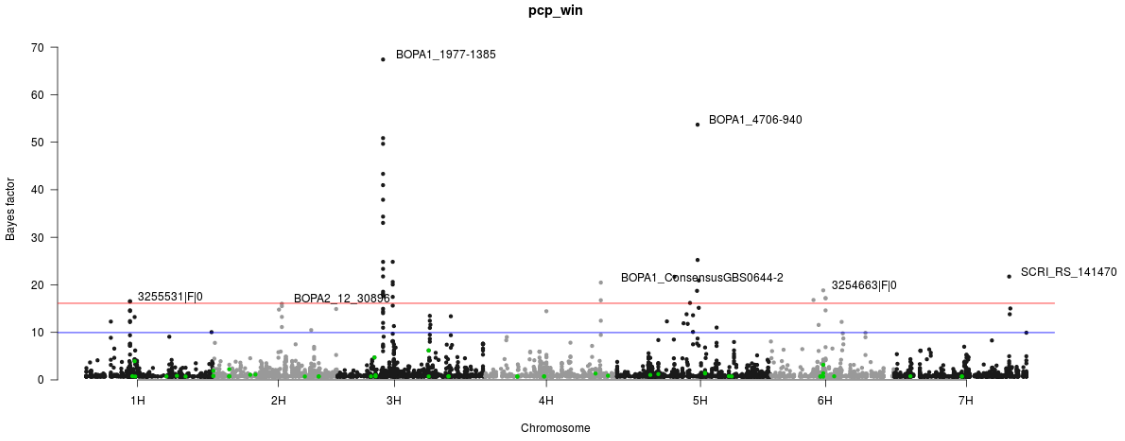
pcp_win
Bayes factor
Chromosome
1H
2H
3H
4H
5H
6H
7H
BOPA1_1977-1385
BOPA1_4706-940
BOPA1_ConsensusGBS0644-2
3254663|F|0
SCRI_RS_141470
3255531|F|0

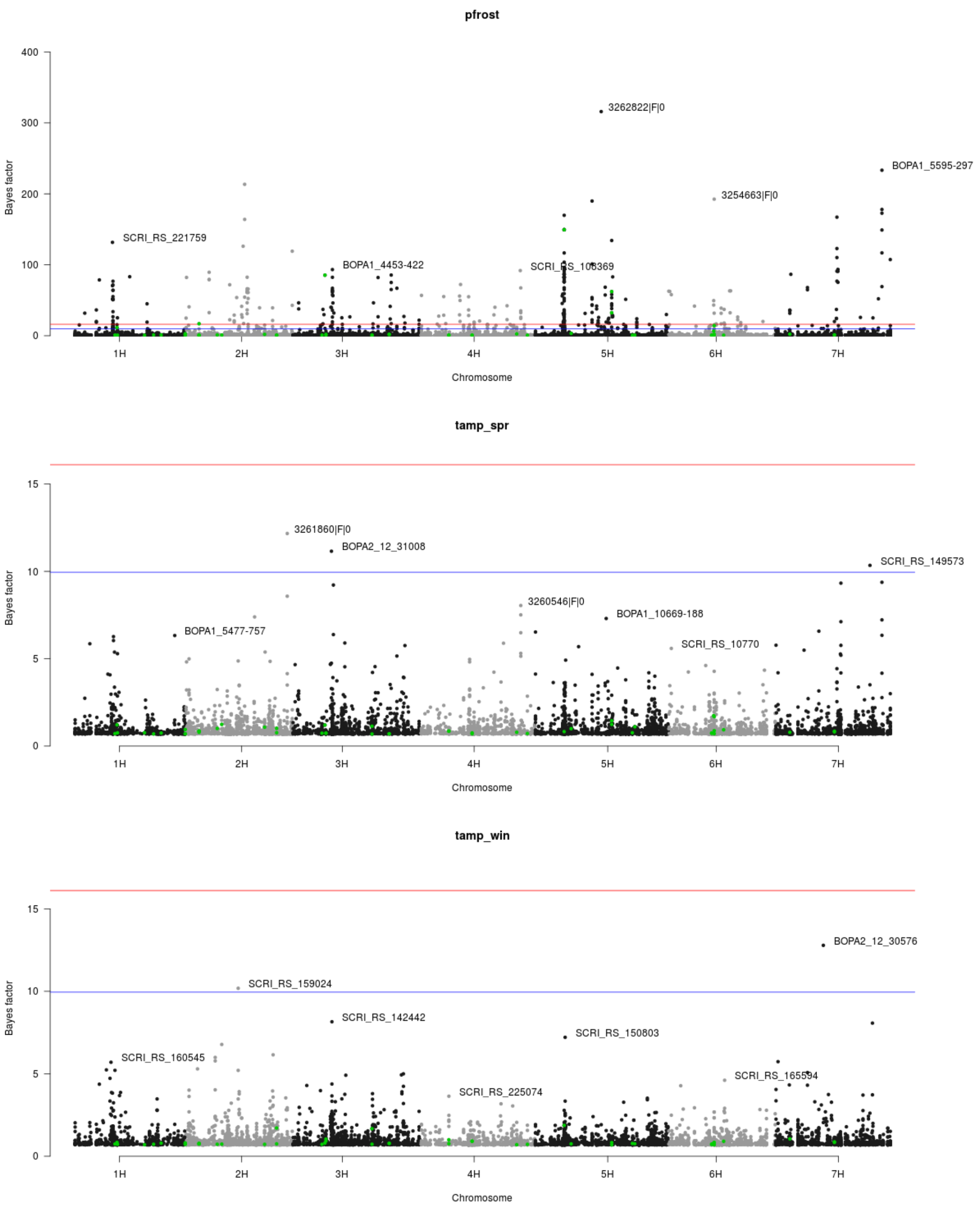

pfrost
Bayes factor
400
300
200
100
0
3262822|F|0
BOPA1_5595-297
3254663|F|0
SCRI_RS_221759
BOPA1_4453-422
SCRI_RS_105369
1H
2H
3H
4H
5H
6H
7H
Chromosome
tamp_spr
Bayes factor
15
10
5
0
3261860|F|0
BOPA2_12_31008
SCRI_RS_149573
3260546|F|0
BOPA1_10669-188
BOPA1_5477-757
SCRI_RS_10770
1H
2H
3H
4H
5H
6H
7H
Chromosome
tamp_win
Bayes factor
15
10
5
0
BOPA2_12_30576
SCRI_RS_159024
SCRI_RS_142442
SCRI_RS_150803
SCRI_RS_160545
SCRI_RS_165594
SCRI_RS_225074
1H
2H
3H
4H
5H
6H
7H
Chromosome

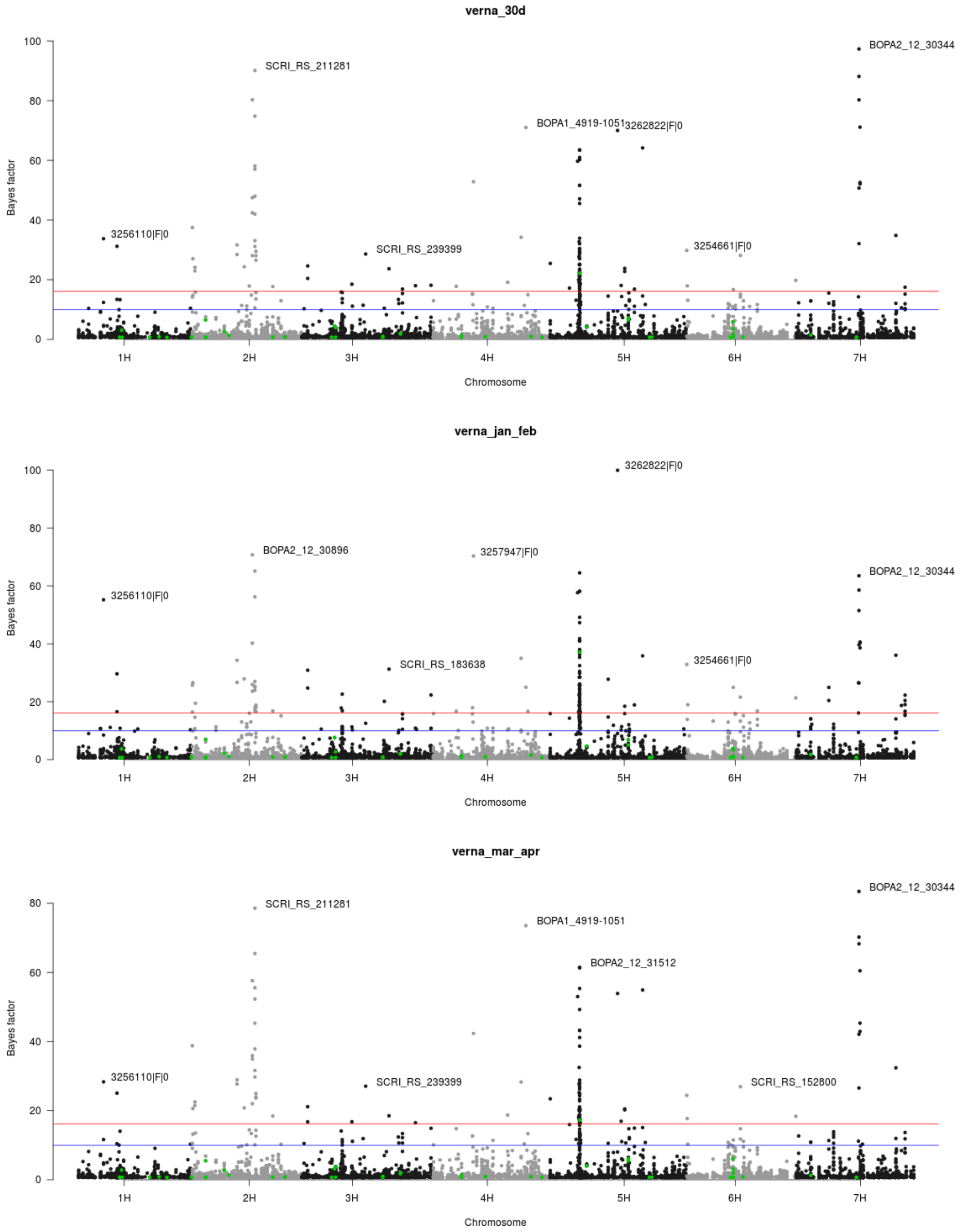

Figure S20. Manhattan plots showing median Bayes Factors (BF) estimated obtained after 5 replicates of association with 7,479 mapped SNPs called in 135 barley landraces and 20 agroclimatic variables. A null (identity) covariance matrix was used during these simulations. A red horizontal line marks the median 99th BF percentile obtained in 5 replicates. A blue horizontal line marks the 99.99th BF percentile obtained in 5 association experiments with 12 dummy variables. Plots produced with a modified function manhattan from R package *qqman* (http://www.biorxiv.org/content/early/2014/05/14/005165).

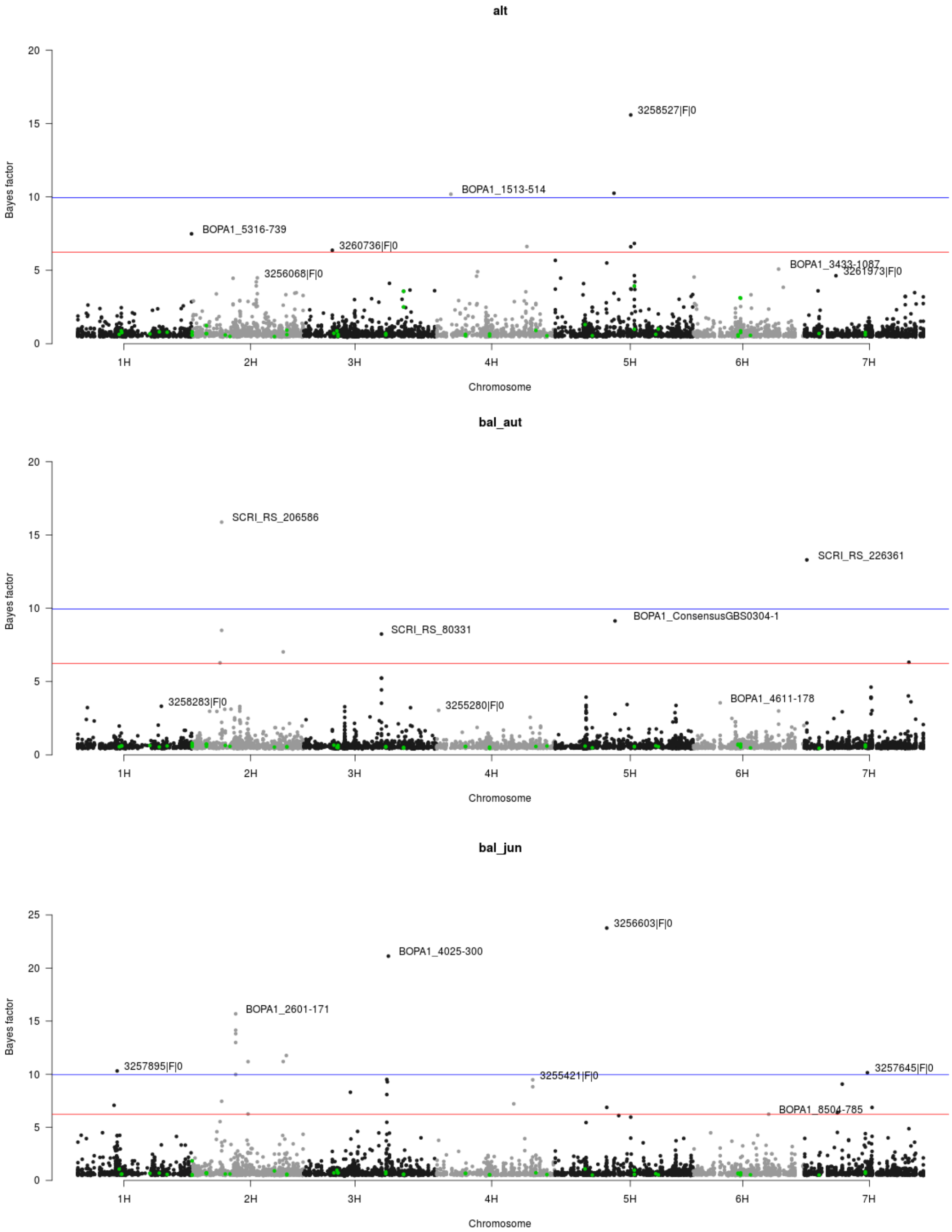

alt
Bayes factor
Chromosome
1H
2H
3H
4H
5H
6H
7H
3258527|F|0
BOPA1_1513-514
BOPA1_5316-739
3260736|F|0
3256068|F|0
BOPA1_3433-1087
3261973|F|0
bal_aut
SCRI_RS_206586
SCRI_RS_226361
BOPA1_ConsensusGBS0304-1
SCRI_RS_80331
3258283|F|0
3255280|F|0
BOPA1_4611-178
bal_jun
3256603|F|0
BOPA1_4025-300
BOPA1_2601-171
3257895|F|0
3255421|F|0
3257645|F|0
BOPA1_8504-785

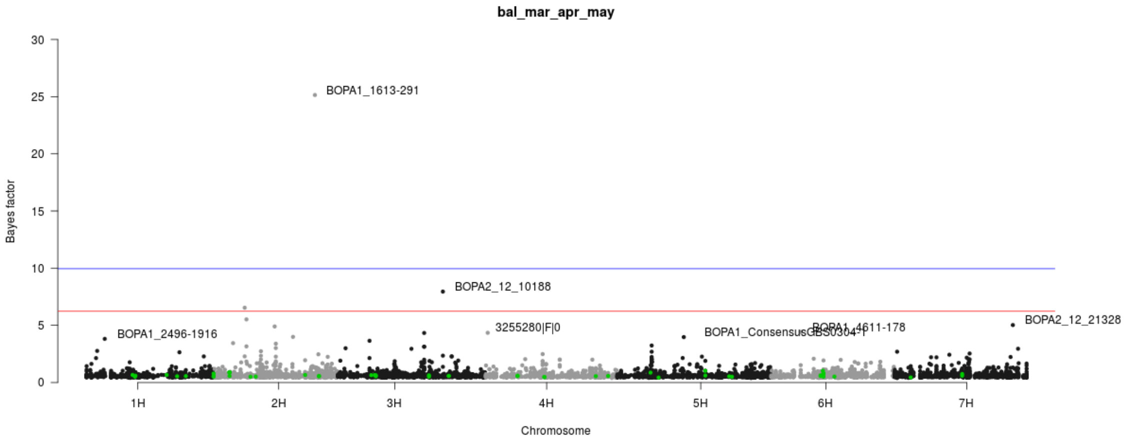
bal_mar_apr_may
Bayes factor
Chromosome
BOPA1_1613-291
BOPA2_12_10188
BOPA1_2496-1916
3255280|F|0
BOPA2_12_21328
1H
2H
3H
4H
5H
6H
7H

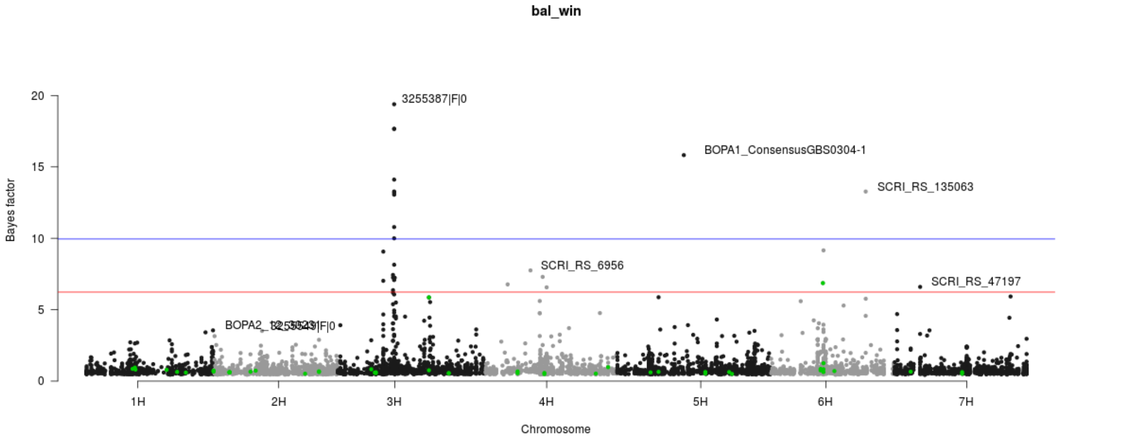
bal_win
Bayes factor
Chromosome
3255387|F|0
BOPA1_ConsensusGBS0304-1
SCRI_RS_135063
SCRI_RS_6956
SCRI_RS_47197
1H
2H
3H
4H
5H
6H
7H

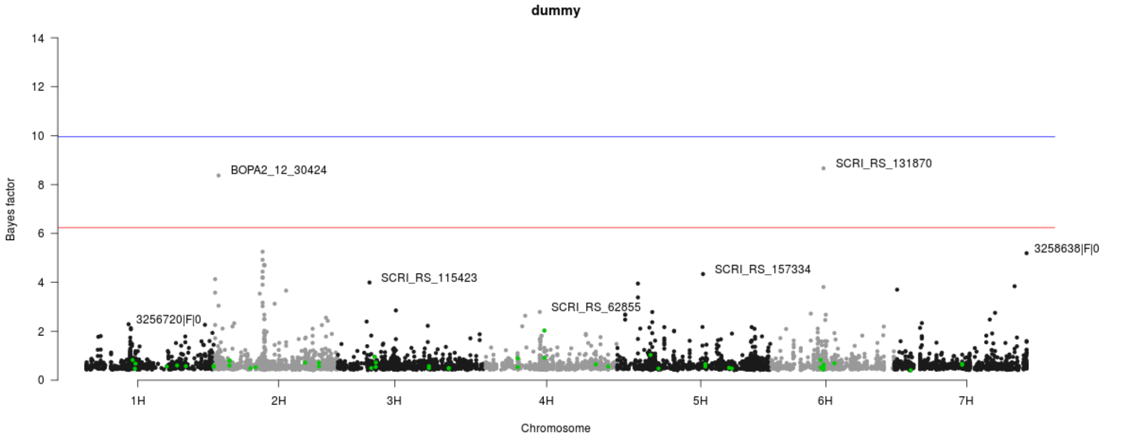
dummy
Bayes factor
Chromosome
BOPA2_12_30424
SCRI_RS_131870
3258638|F|0
SCRI_RS_157334
SCRI_RS_115423
SCRI_RS_62855
3256720|F|0
1H
2H
3H
4H
5H
6H
7H

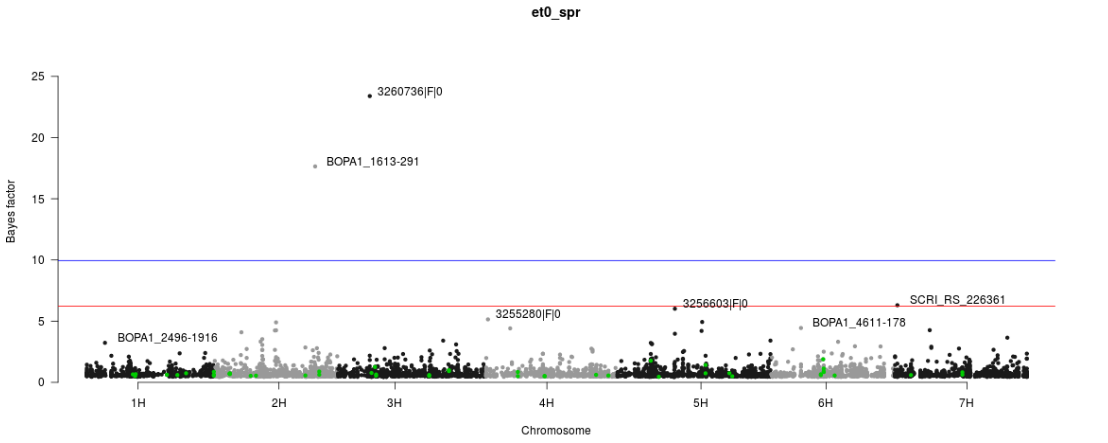
et0_spr
Bayes factor
Chromosome
1H
2H
3H
4H
5H
6H
7H
3260736|F|0
BOPA1_1613-291
3255280|F|0
3256603|F|0
SCRI_RS_226361
BOPA1_4611-178
BOPA1_2496-1916

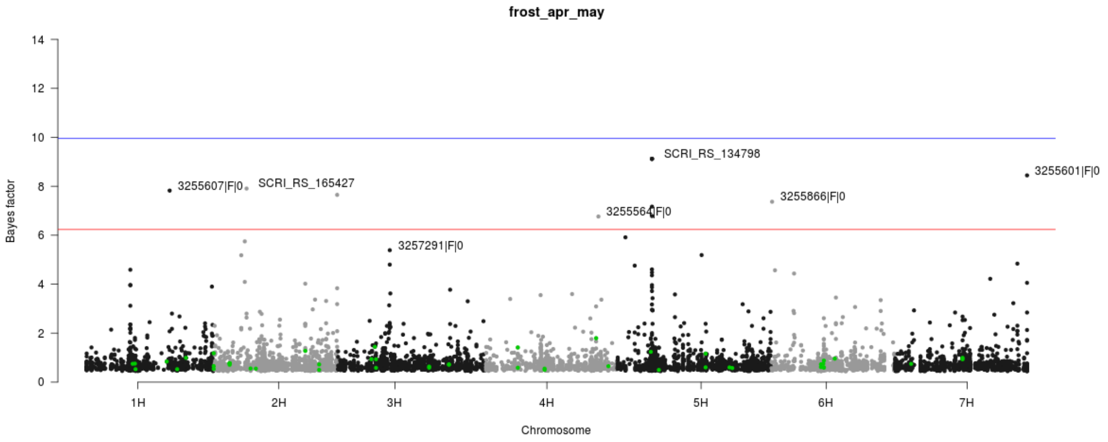
frost_apr_may
Bayes factor
Chromosome
1H
2H
3H
4H
5H
6H
7H
SCRI_RS_134798
3255601|F|0
3255607|F|0
SCRI_RS_165427
3255866|F|0
3255564|F|0
3257291|F|0

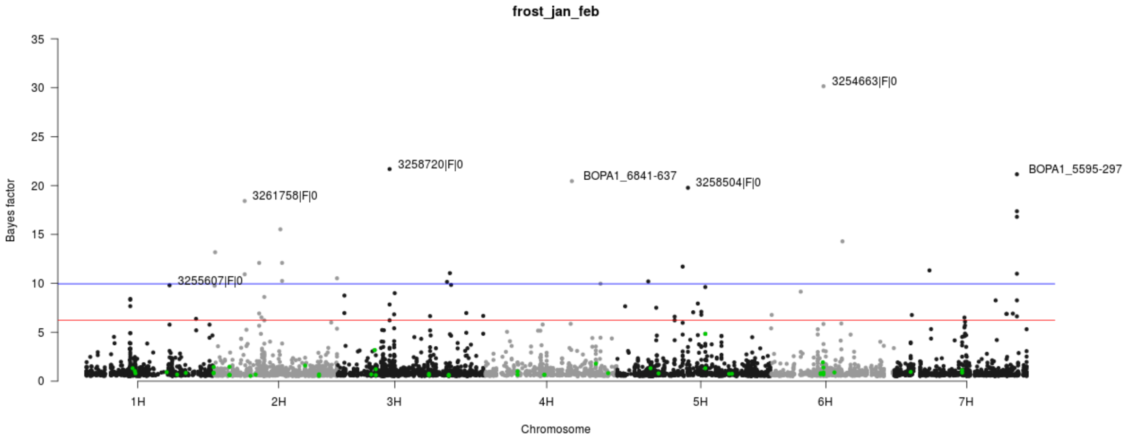
frost_jan_feb
Bayes factor
Chromosome
1H
2H
3H
4H
5H
6H
7H
3254663|F|0
3258720|F|0
BOPA1_6841-637
3258504|F|0
BOPA1_5595-297
3261758|F|0
3255607|F|0

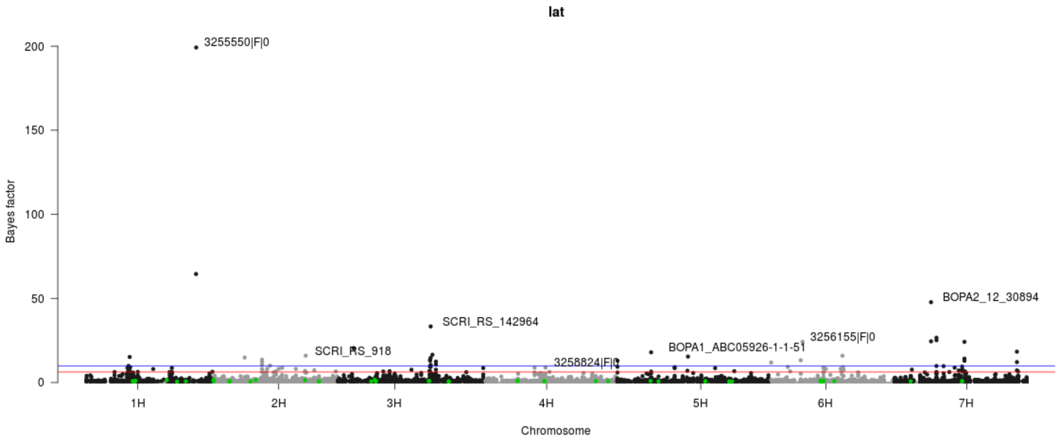
lat
Bayes factor
200
150
100
50
0
3255550|F|0
SCRI_RS_142964
SCRI_RS_918
3258824|F|0
BOPA1_ABC05926-1-1-51
3256155|F|0
BOPA2_12_30894
1H
2H
3H
4H
5H
6H
7H
Chromosome

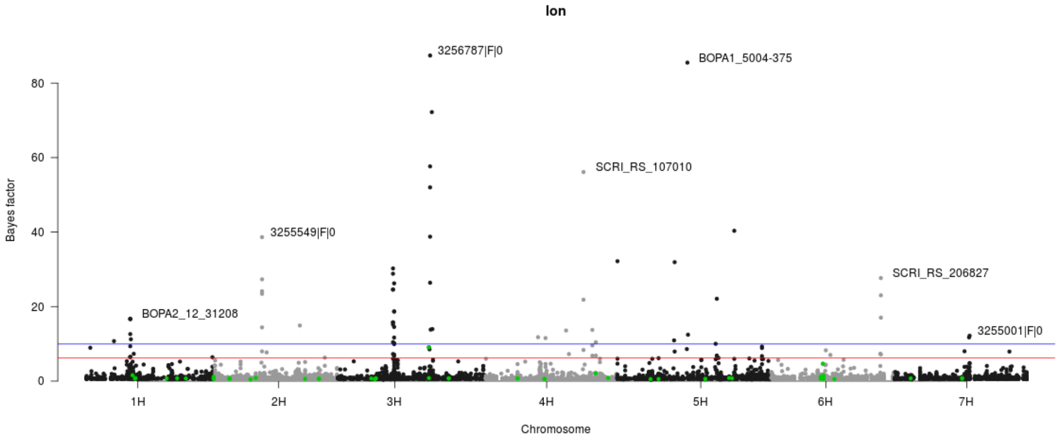
lon
Bayes factor
80
60
40
20
0
3256787|F|0
BOPA1_5004-375
SCRI_RS_107010
3255549|F|0
SCRI_RS_206827
BOPA2_12_31208
3255001|F|0
1H
2H
3H
4H
5H
6H
7H
Chromosome

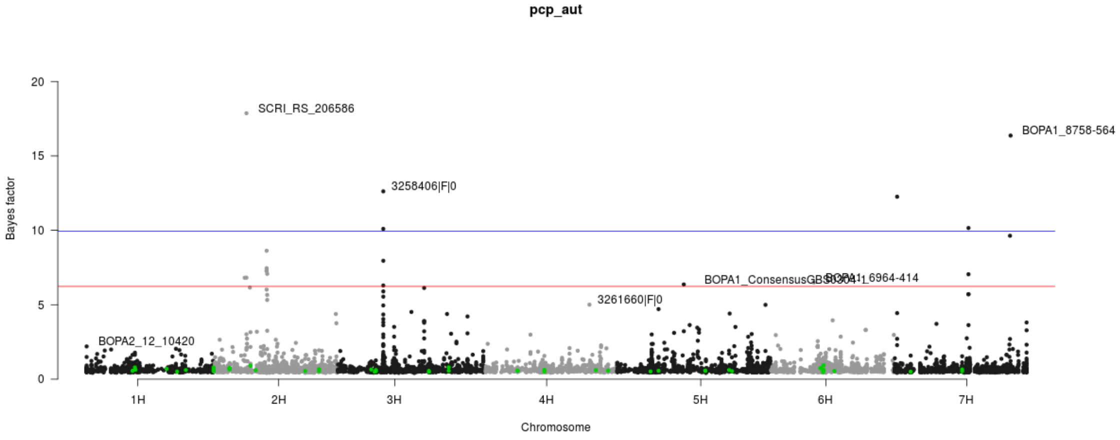
pcp_aut
Bayes factor
20
15
10
5
0
SCRI_RS_206586
BOPA1_8758-564
3258406|F|0
BOPA1_6964-414
3261660|F|0
BOPA2_12_10420
1H
2H
3H
4H
5H
6H
7H
Chromosome

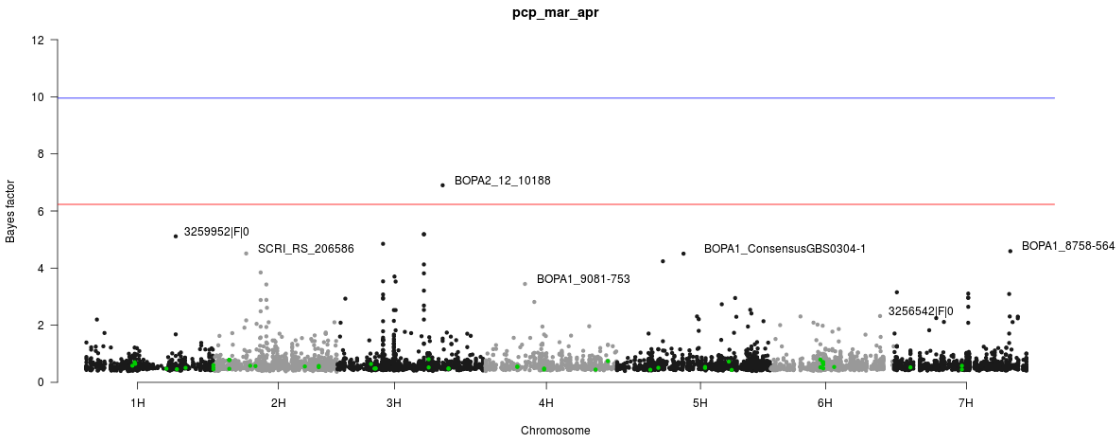
pcp_mar_apr
Bayes factor
Chromosome
1H
2H
3H
4H
5H
6H
7H
BOPA2_12_10188
3259952|F|0
SCRI_RS_206586
BOPA1_ConsensusGBS0304-1
BOPA1_8758-564
BOPA1_9081-753
3256542|F|0

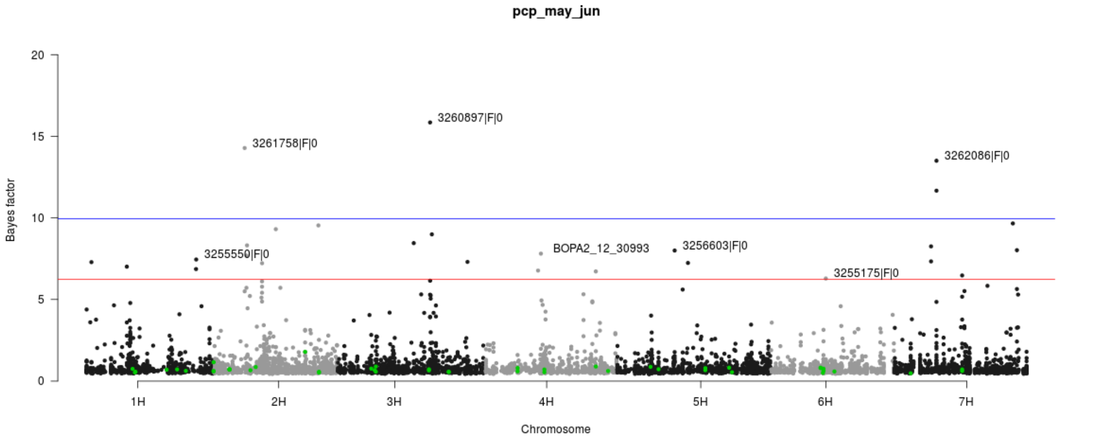
pcp_may_jun
Bayes factor
Chromosome
1H
2H
3H
4H
5H
6H
7H
3260897|F|0
3261758|F|0
3262086|F|0
3255550|F|0
BOPA2_12_30993
3256603|F|0
3255175|F|0

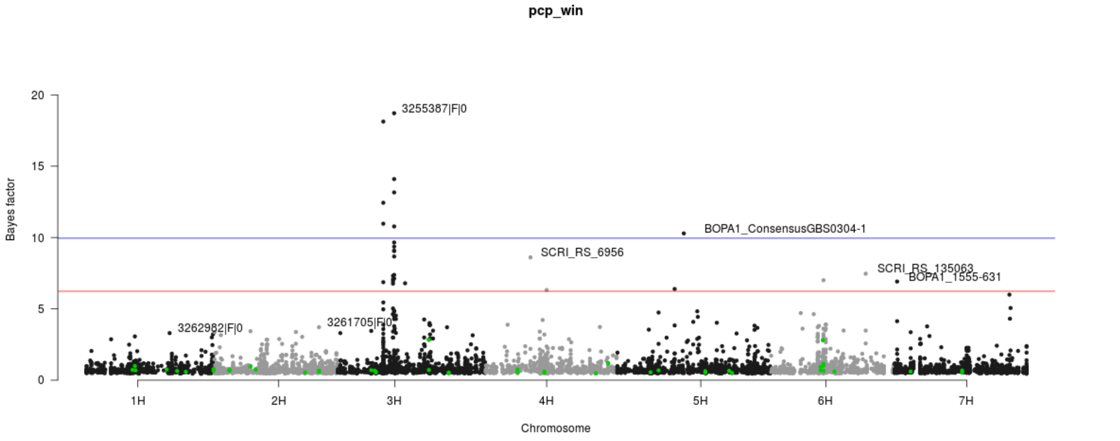
pcp_win
Bayes factor
Chromosome
1H
2H
3H
4H
5H
6H
7H
3255387|F|0
BOPA1_ConsensusGBS0304-1
SCRI_RS_6956
SCRI_RS_135063
BOPA1_1555-631
3262982|F|0
3261705|F|0

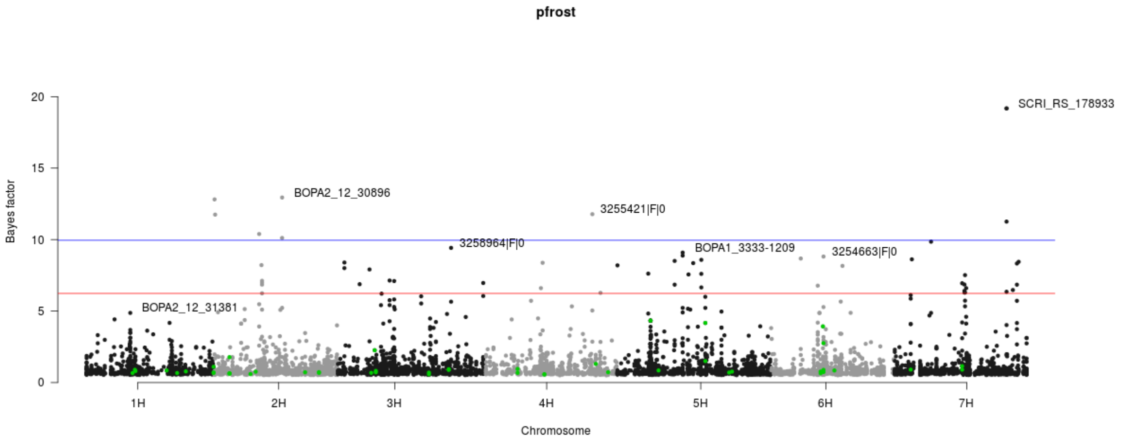
pfrost
Bayes factor
20
15
10
5
0
SCRI_RS_178933
BOPA2_12_30896
3255421|F|0
3258964|F|0
BOPA1_3333-1209
3254663|F|0
BOPA2_12_31381
1H
2H
3H
4H
5H
6H
7H
Chromosome

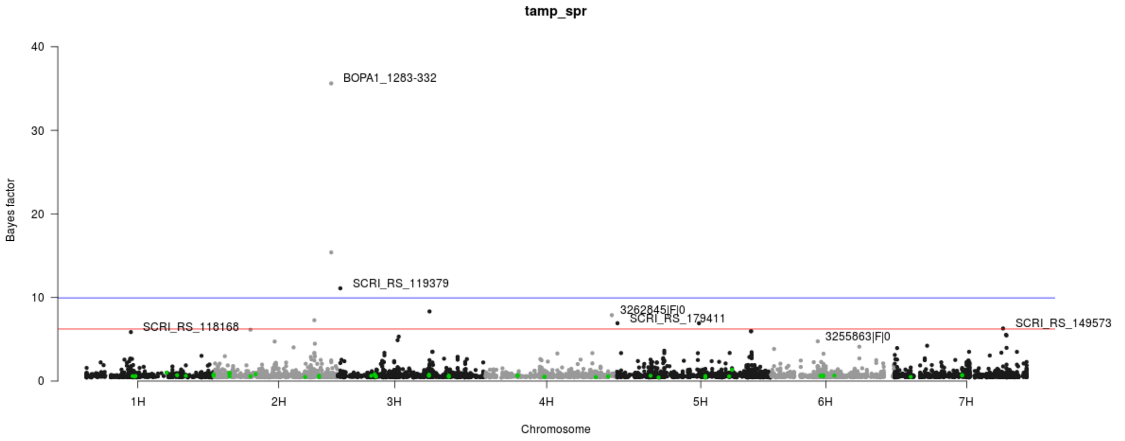
tamp_spr
Bayes factor
40
30
20
10
0
BOPA1_1283-332
SCRI_RS_119379
3262845|F|0
SCRI_RS_179411
SCRI_RS_118168
SCRI_RS_149573
3255863|F|0
1H
2H
3H
4H
5H
6H
7H
Chromosome

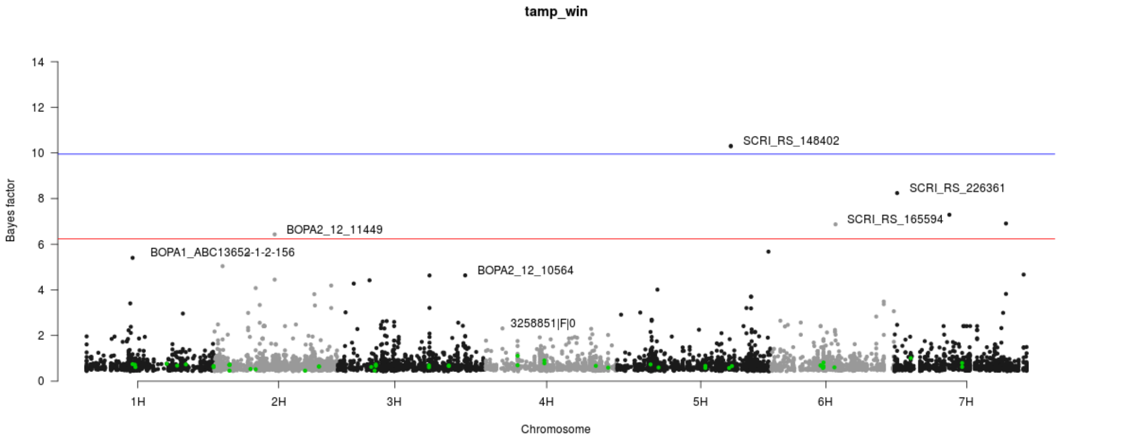
tamp_win
Bayes factor
14
12
10
8
6
4
2
0
SCRI_RS_148402
SCRI_RS_226361
SCRI_RS_165594
BOPA2_12_11449
BOPA1_ABC13652-1-2-156
BOPA2_12_10564
3258851|F|0
1H
2H
3H
4H
5H
6H
7H
Chromosome

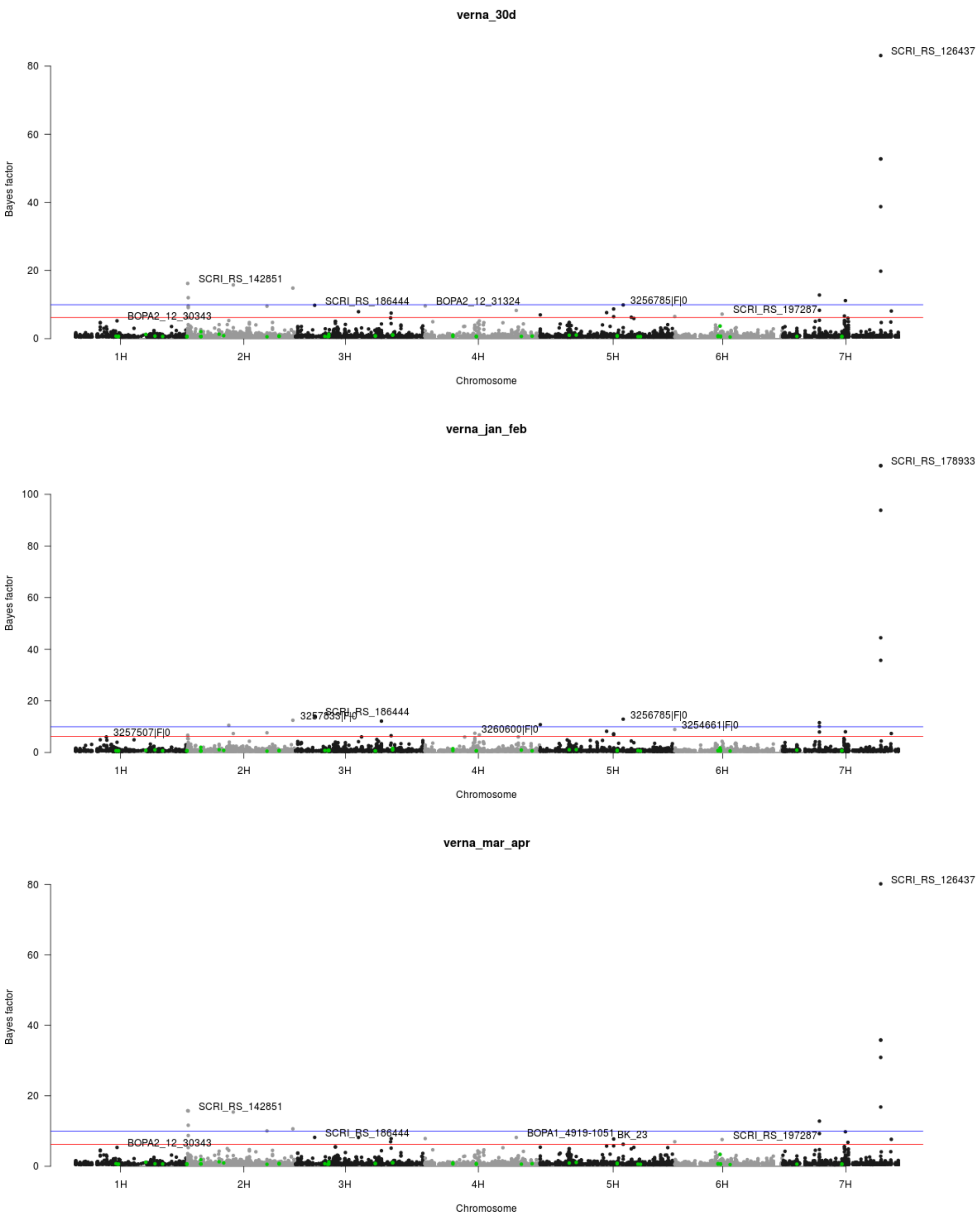

Figure S21. Manhattan plots showing median Bayes Factors (BF) estimated obtained after 5 replicates of association with 7,479 mapped SNPs called in 135 barley landraces and 20 agroclimatic variables. Population structure was captured in a covariance matrix. A red horizontal line marks the median 99th BF percentile obtained in 5 replicates. A blue horizontal line marks the 99.99th BF percentile obtained in 5 association experiments with 12 dummy variables. Plots produced with a modified function manhattan from R package *qqman* (http://www.biorxiv.org/content/early/2014/05/14/005165).

References

Earl, D.E., von Holdt, B.M. (2012). STRUCTURE HARVESTER: a website and program for visualizing STRUCTURE output and implementing the Evanno method. *Conservation Genetics Resources* **4**: 359-361. https://doi.org/10.1007/s12686-011-9548-7

Milne, I., Shaw, P., Stephen, G., Bayer, M., Cardle, L., Thomas, W.T.B., Flavell, A.J., Marshall, D. (2010). Flapjack – graphical genotype visualization. *Bioinformatics* **26**, 3133-3134. https://doi.org/10.1093/bioinformatics/btq580

Perrier, X., Jacquemoud-Collet, J.P. (2006). DARwin software http://darwin.cirad.fr/

Warnes, G.R., Bolker, B., Bonebakker, L., Gentleman, R., Liaw, W.H.A., Lumley, T. … Venables, B. (2016). gplots: Various R Programming Tools for Plotting Data. R package version 3.0.1. https://CRAN.R-project.org/package=gplots

Wei, T., & Simko V. (2016). corrplot: Visualization of a Correlation Matrix. R package version 0.77. https://CRAN.R-project.org/package=corrplot
