# Supplementary material for: Genetic association with high‐resolution climate data reveals selection footprints in the genomes of barley landraces across the Iberian Peninsula
Source: Mol Ecol. 2019 Apr 2;28(8):1994–2012. doi: 10.1111/mec.15009 (PMC6563438; doi:10.1111/mec.15009)
Supplement: Supplementary file 3 [file MEC-28-1994-s003.pdf]

MOLECULAR ECOLOGY

Supplemental Information for:

Genetic association with high-resolution climate data reveals selection footprints in the genomes of barley landraces across the Iberian Peninsula

Bruno Contreras-Moreira, Roberto Serrano-Notivoli, Naheif E. Mohamed, Carlos P. Cantalapiedra, Santiago Beguería, Ana M. Casas, Ernesto Igartua

Supplementary Tables (Excel file with supplementary tables)

- Table S1. Barley accessions studied, including row type and results from the Structure analysis.
- Table S2. Markers used in this study, alleles, missing data and minum allele frequency.
- Table S3. Physical and genetic position of markers used in this study according to Mascher et al. (2017) and Beier et al. (2017), respectively.
- Table S4. Climatic variables corresponding to the coordinates of the collection sites for 135 Spanish barley landraces.
- Table S5. Markers used to calculate the covariance matrix in Bayenv2.
- Table S6. List of genes related to flowering time control and domestication in barley. Physical and genetic positions and closest 9k markers.
- Table S7. List of markers with XtX, heterozygosity, and LD values for each SNP, and in 4 cM sliding windows.
- Table S8. Significant associations between SNP distribution and geographic variables longitude, latitude, altitude, detected with Bayenv2 and LFMM.

Supplementary Figures (PDF file with all supplementary figures)

- Figure S1. Map of observatories of temperature and precipitation that provided daily data from 1981 to 2010.
- Figure S2. Example of the gridded daily dataset of precipitation and temperature…

- Figure S3. Examples of random Gaussian fields used in the analysis as dummy variables…
- Figure S4. Correlation plot of agroclimatic variables...
- Figure S5. Dendrogram corresponding to the hierarchical cluster analysis of agroclimatic variables, with indication of 10 clusters chosen for variable selection.
- Figure S6. Examples of maps of the agroclimatic variables...
- Figure S7. Variables’ loadings in the first component of the principal component analysis of the agroclimatic variables.
- Figure S8. Variables’ loadings in the second component of the principal component analysis of the agroclimatic variables.
- Figure S9. Variables’ loadings in the third component of the principal component analysis of the agroclimatic variables.
- Figure S10. Maps of the first three PCA components of the agroclimatic variables…
- Figure S11. Dendrogram of landraces derived from a median covariance matrix computed by Bayenv2 from 711 SNP markers…
- Figure S12. Distribution of median Bayes Factors (BF, top) and Spearman correlation coefficients (bottom)...
- Figure S13. Structure analysis of 135 barley landraces…
- Figure S14. Plot of the two first coordinates of a factorial analysis run with 8457 markers and 135 barley landraces, with software DARwin 6.0.4…
- Figure S15. Distribution of XtX estimates produced by 3 replicates of Bayenv2...
- Figure S16. Plot of XtX estimates produced by Bayenv2 and BayPass.
- Figure S17. Plots of the seven barley chromosomes, displaying LD, heterozygosity, XtX…

MOLECULAR ECOLOGY

- Figure S18. Graphical genotypes for the regions of chromosomes 3H and 5H, identified as two of the main possible selection footprint between germplasm groups…
- Figure S19. Proportion of total variation (adjusted R2) among accessions for germplasm group distribution explained in RDA by agro-climatic variables or spatial structure…
- Figure S20. Manhattan plots showing median Bayes Factors (BF) estimated…in 135 barley landraces and 20 agroclimatic variables. A null (identity) covariance matrix was used during these simulations…
- Figure S21. Manhattan plots showing median Bayes Factors (BF) estimated...in 135 barley landraces and 20 agroclimatic variables. Population structure was captured in a covariance matrix.
